# Synthesis of side-chain regioregular and main-chain alternating poly(bichalcogenophene)s and an ABC-type periodic poly(terchalcogenophene)†

**DOI:** 10.1039/d0sc00404a

**Published:** 2020-03-10

**Authors:** Huai-Hsuan Liu, Wei-Wei Liang, Yu-Ying Lai, Yen-Chen Su, Hau-Ren Yang, Kuang-Yi Cheng, Sheng-Cih Huang, Yen-Ju Cheng

**Affiliations:** Department of Applied Chemistry, National Chiao Tung University 1001 University Road Hsin-Chu Taiwan yjcheng@nctu.edu.tw; Center for Emergent Functional Matter Science, National Chiao Tung University 1001 University Road Hsinchu 30010 Taiwan; Institute of Polymer Science and Engineering, National Taiwan University Taipei 10617 Taiwan

## Abstract

Three unsymmetrical diiodobichalcogenophenes **SSeI2**, **STeI2**, and **SeTeI2** and a diiodoterchalcogenophene **SSeTeI2** were prepared. Grignard metathesis of **SSeI2**, **STeI2**, **SeTeI2**, and **SSeTeI2** occurred regioselectively at the lighter chalcogenophene site because of its relatively lower electron density and less steric bulk. Nickel-catalyzed Kumada catalyst-transfer polycondensation of these Mg species provided a new class of side-chain regioregular and main-chain AB-type alternating poly(bichalcogenophene)s—**PSSe**, **PSTe**, and **PSeTe**—through a chain-growth mechanism. The ring-walking of the Ni catalyst from the lighter to the heavier chalcogenophene facilitated subsequent oxidative addition, thereby suppressing the possibility of chain-transfer or chain-termination. More significantly, the Ni catalyst could walk over the distance of three rings (*ca.* 1 nm)—from a thiophene unit *via* a selenophene unit to a tellurophene unit—to form **PSSeTe**, the first ABC-type regioregular and periodic poly(terchalcogenophene) comprising three different types of 3-hexylchalcogenophenes.

## Abstract 2

Three unsymmetrical diiodobichalcogenophenes **SSeI2**, **STeI2**, and **SeTeI2** and a diiodoterchalcogenophene **SSeTeI2** were prepared to synthesize a new class of polychalcogenophenes with precisely controlled sequences by catalyst-transfer polycondensation.

## Graphical abstract

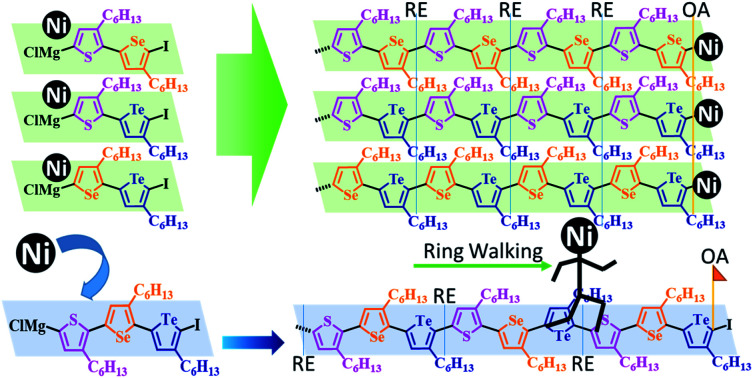

## Introduction

**Fig. 1:**
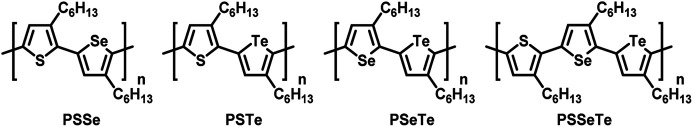
Chemical structures of the alternating poly(bichalcogenophene)s **PSSe**, **PSTe**, and **PSeTe** and the periodic poly(terchalcogenophene) **PSSeTe**.

Conjugated polymers featuring continuous sp^2^/sp-hybridized backbones for extensive delocalization of electrons have been explored widely for several decades.^1–5^ Regioregular poly(3-hexylthiophene) (**P3HT**) exhibiting high charge mobility has been widely applied in organic light-emitting diodes,^6,7^ transistors,^8–14^ and polymer solar cells^15–18^ due to its high crystallinity and well-ordered packing structure in the thin film.^19,20^ As a result, extensive research has been focused on Kumada catalyst-transfer polycondensation (KCTP) for the synthesis of highly regioregular **P3HT** having a low polydispersity index (PDI).^21–36^ However, an intrinsic drawback of **P3HT** and other thiophene-based polymers is the limited absorption window from approximately 300 to 550 nm, thereby hindering their various optoelectronic applications. Structurally similar to thiophene, two other five-membered heterocycles in the chalcogenophene family—selenophene and tellurophene, featuring group-16 Se and Te elements, respectively—have attracted considerable interest because (1) Se and Te atoms are larger and have d-orbitals of higher polarizability (relative to S) to induce strong Se⋯Se and Te⋯Te attractions, potentially strengthening their interpolymer interactions,^37–41^ and (2) as the chalcogen becomes heavier, the π-electrons in selenophene and tellurophene tend to adopt a more quinoidal character with higher coplanarity, giving rise to narrower band gaps and bathochromic shifts in their absorption.^42–50^ The homopolymers 3-alkylselenophene (**P3AS**)^51,52^ and 3-alkyltellurophene (**P3ATe**)^53,54^ have also been prepared using the KCTP. We were interested in studying the effects of incorporating two different chalcogenophenes into a single polymer to generate a new class of poly(bichalcogenophene)s. By integrating thiophene, selenophene, and tellurophene units in a single polymer in a controlled sequence, the properties of the polymer could presumably be tailored specifically. In particular, we envisaged that positioning selenophene and tellurophene units, having five-membered ring structures similar to thiophene, into the polymers would allow fine-tuning of the optical and electronic properties without substantially affecting the conformation of the main chain, thereby maintaining high crystallinity. To date, the synthesis of random^55–59^ and block poly(bichalcogenophene)s^60–63^ [*e.g.*, poly(thiophene-*block*-selenophene)] have been realized simply by controlling the sequence of addition of the monomer. Nevertheless, the synthesis of main-chain alternating and side-chain regioregular AB-type poly(bichalcogenophene)s remains a challenge, and these materials have not been well explored.^64–66^ In particular, tellurophene-incorporated alternating poly(bichalcogenophene)s have never been described previously. In this present study, we developed a new class of AB-type alternating poly(bichalcogenophene)s constructed from 3-hexylthiophene, 3-hexylselenophene, and 3-hexyltellurophene units (Fig. 1). After careful design of the unsymmetrical bichalcogenophene monomers, we synthesized poly(3-hexylthiophene-*alt*-3-hexylselenophene) (**PSSe**), poly(3-hexylthiophene-*alt*-3-hexyltellurophene) (**PSTe**), and poly(3-hexylselenophene-*alt*-3-hexyltellurophene) (**PSeTe**) through KCTP operated in a chain-growth manner. Furthermore, we report the first example of a regioregular ABC-type periodic poly(terchalcogenophene), poly(3-hexylthiophene-*per*-3-hexylselenophene-*per*-3-hexyltellurophene) (**PSSeTe**).

## Results and discussion

### Molecular design

**Fig. 2:**
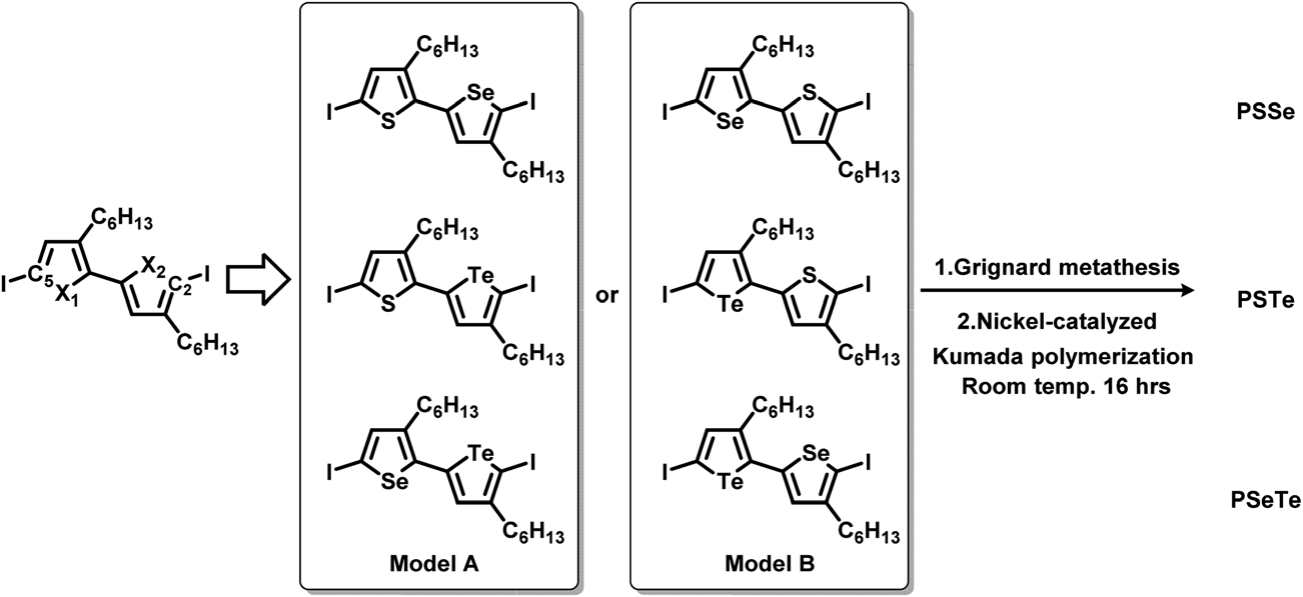
Design of six unsymmetrical monomers, in models A and B, for the synthesis of **PSSe**, **PSTe**, and **PSeTe**.

To synthesize the three alternating polymers **PSSe**, **PSTe**, and **PSeTe** through KCTP, we designed three corresponding unsymmetrical diiodobichalcogenophene monomers having a head-to-tail arrangement of their hexyl side chains. There are two possible isomeric arrangements for the unsymmetrical monomers (Fig. 2): one with the heavier chalcogen placed at X_2_ (*i.e.*, model A: X_1_ = S, X_2_ = Se; X_1_ = S, X_2_ = Te; X_1_ = Se, X_2_ = Te) and the other with the heavier chalcogen placed at X_1_ (*i.e.*, model B: X_1_ = Se, X_2_ = S; X_1_ = Te, X_2_ = S; X_1_ = Te, X_2_ = Se). Achieving high selectivity between the two reactive carbon atoms (C_2_ and C_5_) during Grignard metathesis would be an important criterion toward obtaining polymers of high head-to-tail regioregularity after the nickel-catalyzed KCTP.^27,67^ We used Hirshfeld charge analysis to calculate the charge distributions at C_2_ and C_5_ of the diiodobichalcogenophene monomers in models A and B (Table 1). Because of the proximity of the electron-donating aliphatic substituent and the heavier chalcogen, C_2_ bore a more negative charge than C_5_ in model A; in other words, a pronounced charge difference existed between C_5_ and C_2_ in model A and, furthermore, C_5_ is less sterically hindered than C_2_. Considering both the electronic and steric effects, we envisioned that C_5_ in model A would be more susceptible to Mg/I exchange (Grignard metathesis) than C_2_, potentially leading to highly regioregular polymers. In contrast, the C_2_ and C_5_ atoms in model B had very similar charge densities, suggesting that Grignard metathesis might occur with no selectivity. Consequently, we chose the three monomers in model A for polymerization.

**Table 1:** Hirshfeld charge analysis of the charge distributions at atoms C_2_ and C_5_ of six unsymmetrical diiodonated bichalcogenophenes, calculated at the cam-B3LYP/6-311G(d,P) and LanL2DZ(d,p) levels of theory

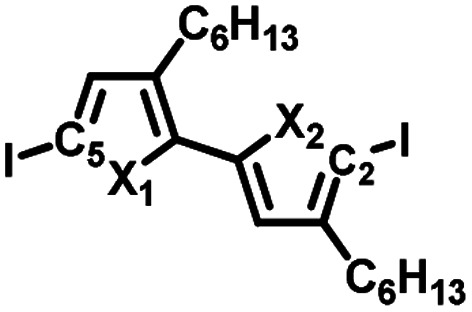	C_2_	C_5_
X_1_ = S; X_2_ = Se	−0.036	−0.025
X_1_ = S; X_2_ = Te	−0.042	−0.026
X_1_ = Se; X_2_ = Te	−0.043	−0.032
X_1_ = Se; X_2_ = S	−0.030	−0.030
X_1_ = Te; X_2_ = S	−0.031	−0.036
X_1_ = Te; X_2_ = Se	−0.038	−0.036

### Synthetic procedures

**Scheme 1:**
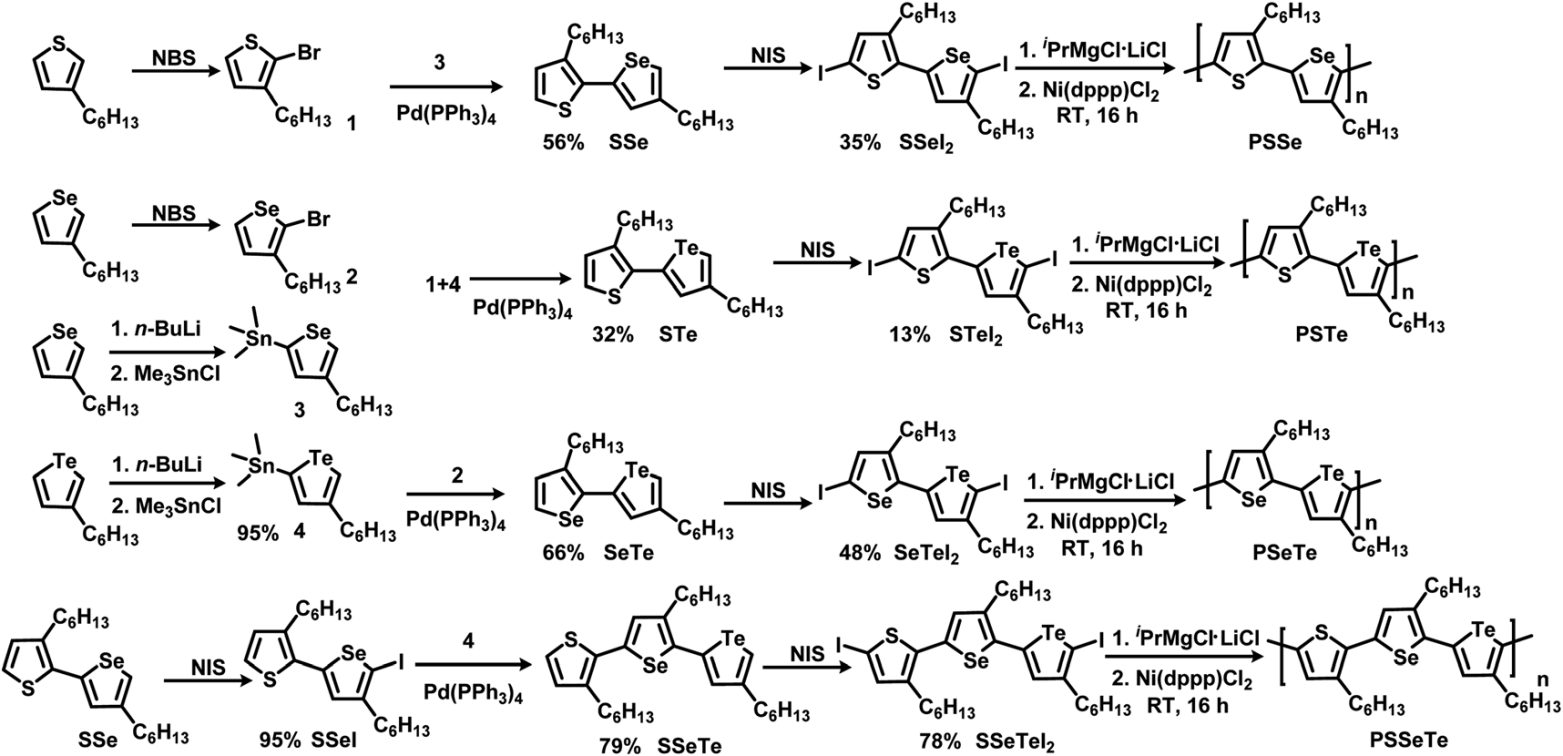
Synthesis of the monomers **SSeI2**, **STeI2**, **SeTeI2**, and **SSeTeI2** and their polymerization.

Scheme 1 illustrates the synthesis of the three monomers **SSeI2**, **STeI2**, and **SeTeI2**. Bromination of 3-hexylthiophene and 3-hexylselenophene in the presence of *N*-bromosuccinimide (NBS) afforded compounds **1** and **2**, respectively. Stannylation of 3-hexylselenophene and 3-hexyltellurophene using *n*-BuLi and Me_3_SnCl furnished compounds **3** and **4**, respectively. The three unsymmetrical bichalcogenophenes **SSe**, **STe**, and **SeTe** were obtained through Stille coupling of **1** and **3**, **1** and **4**, and **2** and **4**, respectively. The three **SSeI2**, **STeI2**, and **SeTeI2** monomers in model A were prepared through iodination of **SSe**, **STe**, and **SeTe**, respectively, with *N*-iodosuccinimide (NIS). Scheme 1 also presents the synthesis of the terchalcogenophene monomer **SSeTeI2**. Iodination of **SSe** with one equivalent of NIS afforded **SSeI** selectively; it was Stille coupled with compound **4** to yield **SSeTe**. Iodination of **SSeTe** with NIS provided **SSeTeI2** in a yield of 78%.

### Selectivity of Grignard metathesis

To confirm the regioselectivity of Grignard metathesis, we conducted model studies prior to performing the polymerizations. After treatment of **SSeI2**, **STeI2**, and **SeTeI2** with one equivalent of isopropylmagnesium chloride lithium chloride complex (^*i*^PrMgCl·LiCl) to induce Grignard metathesis, the reactions were quenched with aqueous NH_4_Cl to replace the reaction site with a proton. We used ^1^H NMR spectroscopy to examine the crude products (Fig. S1a†). If the Grignard metathesis had occurred at the C_2_ position, three singlets should have appeared in the aromatic region of the ^1^H NMR spectrum; if it had occurred at the C_5_ position, one singlet and two doublets should have been expected. We observed one singlet and two doublets in the spectra of each of the model monomers, revealing that the Grignard metathesis had taken place predominantly at the C_5_ atom. This phenomenon also occurred for the **SSeTeI2** monomer, the ^1^H NMR spectrum of which featured two singlets and two doublets (Fig. S1b†), implying that its Grignard metathesis also occurred at the C_5_ position, rather than at the C_2_ position. For reference, we synthesized another isomeric **SSeI2** monomer, of the model B type, and performed the same experiment. Fig. S2† reveals two major products, resulting from protonation at both the C_2_ and C_5_ positions, after quenching with NH_4_Cl_(aq)_, confirming that the unsymmetrical bichalcogenophenes in model B did not exhibit selectivity toward Grignard metathesis. Thus, we were correct in choosing model A to achieve high selectivity in Grignard metathesis.

### Polymer synthesis and structure identification

We treated the monomers **SSeI2**, **STeI2**, **SeTeI2**, and **SSeTeI2** with 1.0 equivalent of ^*i*^PrMgCl·LiCl and then performed Ni(dppp)Cl_2_-catalyzed KCTP at room temperature to yield the desired polymers **PSSe**, **PSTe**, **PSeTe**, and **PSSeTe**, respectively (Scheme 1). All the polymers were precipitated using 6 M HCl/MeOH solution and then they were washed with MeOH. Because of the limited solubility of the polymers, we used high-temperature gel permeation chromatography (GPC), with trichlorobenzene as the eluent, to determine their molecular weights. The GPC data confirmed the excellent control over the molecular weights with the relatively low polydispersity (Table 2). The molecular weight of **PSTe** increased linearly (6800, 14 400, and 30 800 g mol^−1^) upon increasing the monomer/catalyst molar ratio (25, 50 and 100, respectively), confirming that the reaction had the characteristics of catalyst-transfer polycondensation.^27,35^ The polydispersity (PDI) of **PSSe** is a little bit larger than the previous report.^66^ It should be noted that we used diiodobichalcogenophene monomers for polymerization. Compared to bromine or chlorine, the larger atomic radius of iodine may increase the activation energy for transmetalation during the polymerization, resulting in the higher PDI.^68^

**Table 2:** Molecular weights, PDIs, and regioregularities of the polymers

Polymer	*M*/cat.	*M*_n_ a	PDI	RR
**PSSe**	50	16 500	1.38	92%
**PSeS**	20	4700	2.59	85%
**PSTe**	25	6800	1.34	94%
	50	14 400	1.33	
	100	30 800	1.49	
**PSeTe**	50	23 900	1.47	91%
**PSSeTe**	50	20 600	1.57	94%

a Measured through high-temperature GPC at 160 °C, using polystyrene standards and 1,2,4-trichlorobenzene as the eluent. All the concentrations of polymeric solutions are 1 mg mL^−1^.

We used ^1^H and ^13^C NMR spectroscopy to investigate the chemical structures and regioregularities of the new poly(bichalcogenophene)s and poly(terchalcogenophene). The ^1^H NMR spectra of the polymers **PSSe**, **PSTe**, and **PSeTe** all featured two well-defined aromatic singlets representing the two kinds of protons on the two different 3-hexylchalcogenophene rings (Fig. S3†). Similarly, the spectrum of **PSSeTe** featured three singlets representing the three different 3-hexylchalcogenophene rings. The protons on the first carbon atoms of the hexyl groups appeared at 2.6–2.8 ppm; those on the terminal carbon atoms of the hexyl groups appeared at 0.91–0.93 ppm. Through integration of the signals of the first sets of protons, we estimated the head-to-tail regioregularity to be greater than 90%.^67^ The ^13^C NMR spectra (Fig. S4†) featured eight well-defined aromatic peaks for **PSSe**, **PSTe**, and **PSeTe** and 12 for **PSSeTe**, suggesting that these new polychalcogenophenes featured alternating and periodic main chains characterized by regioregular head-to-tail hexyl groups. Dependence of the number average molecular weight (*M*_n_) and PDI on monomer conversion for **PSTe** is shown in Table S1 and Fig. S5† where the *M*_n_ increased linearly with the conversion, indicating the controlled polymerization process. MALDI-TOF mass spectrometry was used to characterize the end-groups of **PSSeTe** (Fig. S6†). The major peaks of **PSSeTe** matched those expected for the (**SSeTe**)_*n*_ structure (where *n* is the number of repeat units) terminated with an iodine and a hydrogen (I/H) atom. Thus, the MALDI-TOF-MS was also in good agreement with a mechanism involving catalyst-transfer polycondensation.

### Plausible mechanism

**Scheme 2:**
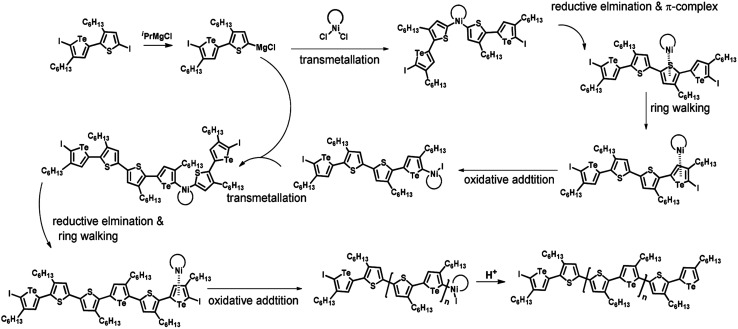
Proposed mechanism for catalyst-transfer polycondensation of **PSTe***via* ring-walking.

According to the mechanism of catalyst-transfer polycondensation proposed by Yokozawa *et al.*^27^ and research through theoretical calculations, Scheme 2 presents our suggested mechanism for the polymerization, taking **STeI2** as an example. Initially, **STeI2** undergoes regioselective Grignard metathesis to form a nucleophilic **ITeS**-MgCl intermediate, which doubly attacks the Ni(dppp)Cl_2_ catalyst for ligand transfer. Following reductive elimination, the Ni species is coordinated to and stabilized by the π-electron bichalcogenophenes.^69^ We suspect that the stronger chelation ability of the heavier Te atom (relative to a S atom) drives the Ni atom to shift from thiophene to tellurophene through a selective “ring-walking” process. The chain grows through a repetitive sequence of transmetalation, reductive elimination, Ni π-complexation, ring-walking, and oxidative addition. Eventually, the polymerization is terminated by a proton to give the final polymer chain. Based on this mechanism, the polymer would contain repeating biaryl or triaryl units with I and H atoms at the chain ends [I(biaryl)_*n*_H or I(triaryl)_*n*_H, respectively]. Notably, the successful synthesis of **PSSeTe** suggests that the Ni catalyst could walk smoothly over three rings: from the thiophene unit, *via* the selenophene unit, to the tellurophene unit. To support this hypothesis, we performed theoretical calculations to estimate the complexation energies of the Ni catalyst with the independent chalcogenophenes. Table 3 reveals that the complexation energies of a Ni(dppp) species with 2-ethylthiophene (complex 1), 2-ethylselenophene (complex 2), and 2-ethyltellurophene (complex 3) were approximately −15.1, −17.3, and −19.3 kcal mol^−1^, respectively. With increased complexation energy, the migration of the Ni complex would be thermodynamically favored from the thiophene unit *via* the selenophene unit to the tellurophene unit, in turn facilitating the subsequent oxidative addition at the iodotellurophene chain end. Thus, our rational design of the four unsymmetrical monomers in model A not only allowed regioselective Grignard metathesis but also favored selective ring-walking, leading to highly regioregular polychalcogenophenes through the KCTP mechanism. In contrast, the polymer **PSeS** prepared from the isomeric model-B monomer **SSeI2** featured (Table 2) much higher polydispersity (2.59) and lower regioregularity (85%), suggesting that the Ni catalyst was more likely to dissociate from the polymer, leading to chain-transfer and chain-termination reactions.

**Table 3:** Association energies (Δ*G*) for the interactions of Ni(dppp) with 2-ethylthiophene, 2-ethylselenophene, and 2-ethyltellurophene

	Complex 1	Complex 2	Complex 3
	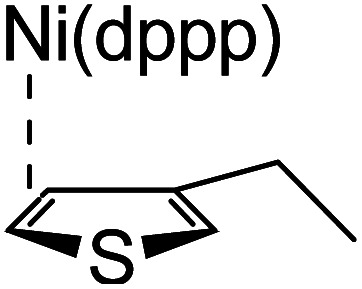	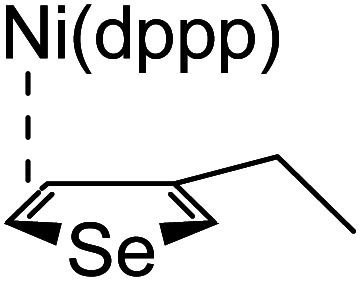	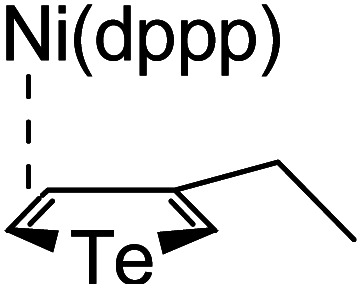
	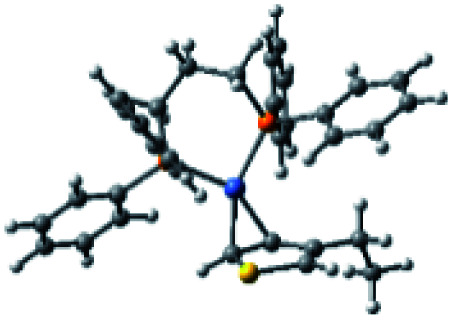	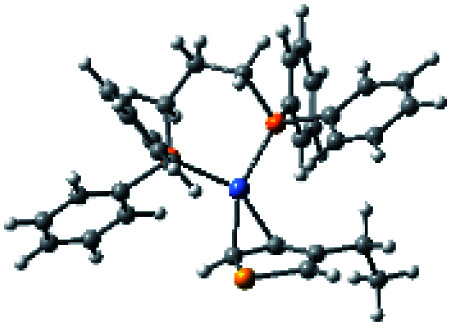	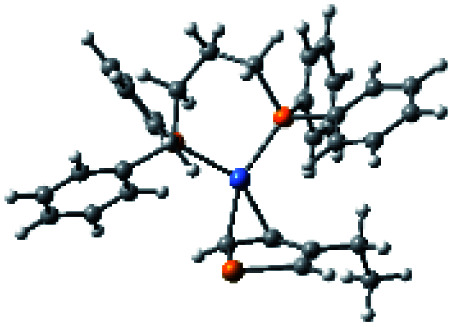
Δ*G*	−15.1 kcal mol^−1^	−17.3 kcal mol^−1^	−19.3 kcal mol^−1^

### Optical properties

**Fig. 3:**
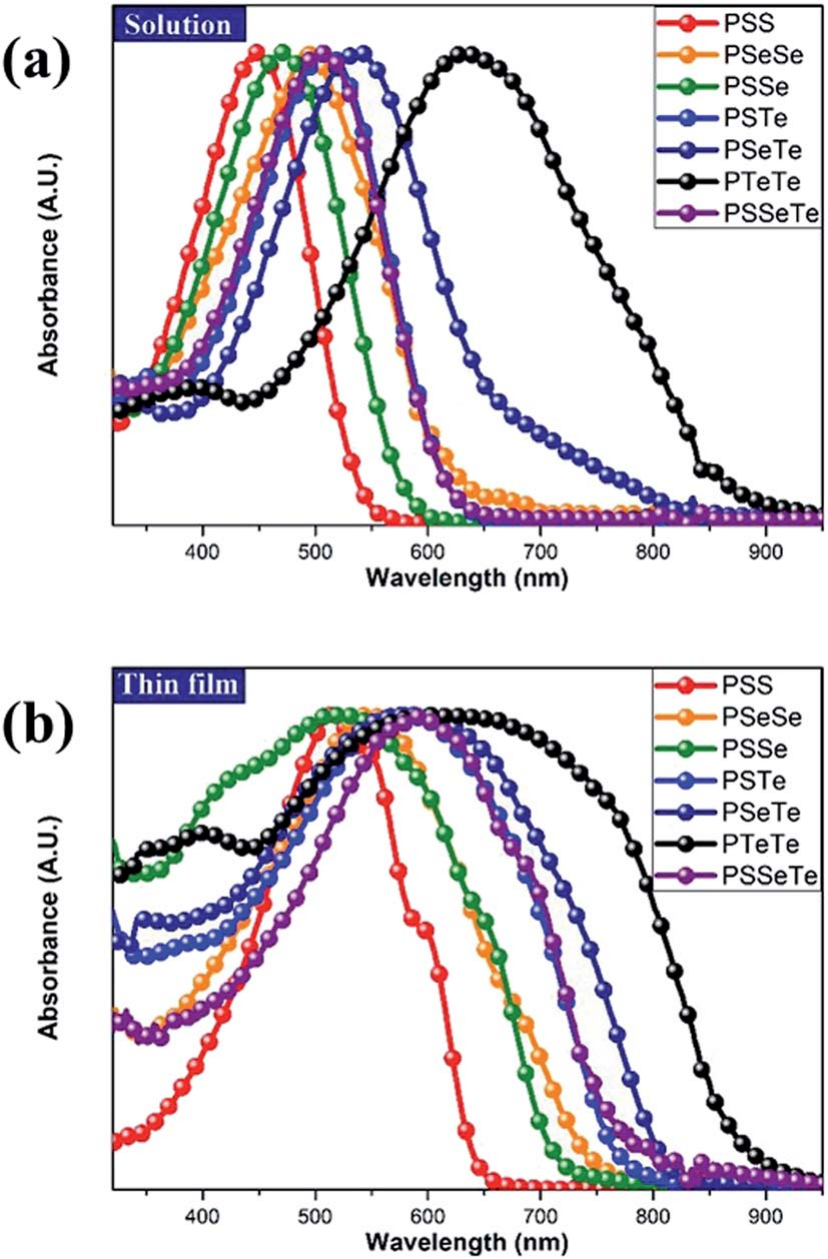
Absorption spectra of **PTeTe**, **PSeTe**, **PSSeTe**, **PSTe**, **PSeSe**, **PSSe**, and **PSS** (a) in *o*DCB solution and (b) as thin films.

To investigate the effect of the chalcogen on the optical absorption behavior of the polychalcogenophenes, we recorded the UV-Vis spectra of our four new polymers **PSSe**, **PSTe**, **PSeTe**, and **PSSeTe** as well as those of the three homopolymers **PSS** (**P3HT**), **PSeSe** (**P3HS**), and **PTeTe** (**P3HTe**). Fig. 3 presents the UV-Vis spectra of all of these polymers in solution (*o*-dichlorobenzene) and in the solid state; Table 4 summarizes the absorption parameters and the band gaps. In solution, the values of *λ*_max_ of the polymers were highly dependent on the chemical composition: **PTeTe** (631 nm) > **PSeTe** (541 nm) > **PSTe** (511 nm) > **PSeSe** (494 nm) > **PSSe** (471 nm) > **PSS** (448 nm). Upon incorporating heavier chalcogenophenes (selenophene and tellurophene) into the polymers, the absorption red-shifted with concomitant broadening of the full width at half maximum (FWHM) of the band. The ability of the chalcogenophene to red-shift the absorption of the polymer followed the order tellurophene > selenophene > thiophene. Thus, the absorption of the polychalcogenophene in the range from 300 to 800 nm could be tuned subtly by carefully selecting the combination of chalcogenophenes in a single polymer. Furthermore, these polymers exhibited significant red-shifting and broadening of their absorptions upon proceeding from solution to the solid state, indicating that strong intermolecular interactions existed between the chalcogenophene moieties. Notably, the tellurophene-containing polymer **PSeTe**, without featuring any electron-deficient units, possessed a relatively small band gap and had an onset wavelength of 800 nm.

**Table 4:** UV-Vis absorption spectral properties of the polymers

	*λ*_max_ (nm)		*λ*_onset_ (nm)	*E* ^opt^_g_ (eV)
	*o*DCB^a^ (FWHM)	Film		
**PSS**	448 (119)	514	643	1.93
**PSSe**	471 (140)	523	711	1.74
**PSeSe**	494 (167)	547	746	1.66
**PSTe**	511 (143)	576	770	1.61
**PSSeTe**	497 (147)	593	777	1.60
**PSeTe**	541 (167)	589	811	1.53
**PTeTe**	631 (246)	632	877	1.41

a *o*-Dichlorobenzene.

### Electrochemical properties

**Fig. 4:**
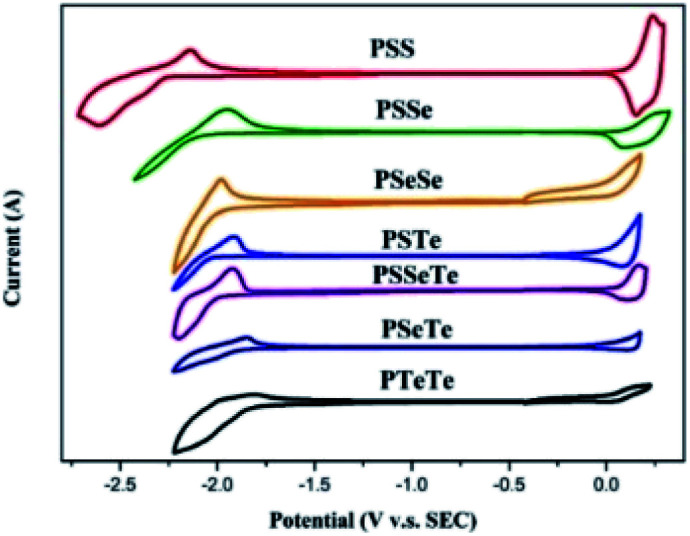
CV plots of the polymer films, relative to ferrocene, revealing their oxidation and reduction onsets.

We used cyclic voltammetry to evaluate the electrochemical properties of the polymers and determine their HOMO and LUMO energy levels (Fig. 4, Table 5). When a heavier chalcogenophene was incorporated, the HOMO was slightly elevated (higher-lying) while the LUMO had descended (lower-lying) more significantly. Thus, the HOMO and LUMO energy levels, along with the band gaps, could be controlled by combining different chalcogenophenes into a single polymer.

**Table 5:** CV data of the polymers

	LUMO (eV)	HOMO (eV)	*E* ^ele^_g_ (eV)
**PSS**	−2.46	−4.89	2.43
**PSSe**	−2.70	−4.88	2.18
**PSTe**	−2.76	−4.82	2.06
**PSeSe**	−2.78	−4.81	2.03
**PSSeTe**	−2.80	−4.86	2.06
**PSeTe**	−2.92	−4.81	1.89
**PTeTe**	−2.96	−4.77	1.81

### Thin film morphologies

**Fig. 5:**
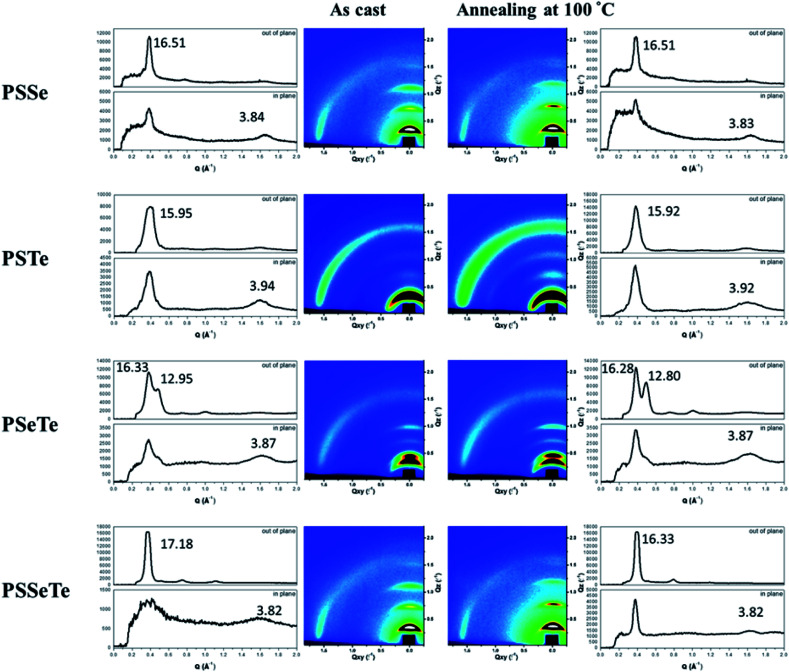
2D GIWAXS images of the polymers and their corresponding 1D in-plane and out-of-plane patterns, recorded before and after thermal annealing at 100 °C.

We used grazing-incidence wide-angle X-ray scattering (GIWAXS) to investigate the morphologies of the polychalcogenophenes. Because of the relatively poor solubility of the polychalcogenophenes, we selected *o*-dichlorobenzene as the processing solvent. Fig. 5 presents the two-dimensional (2D) images and corresponding one-dimensional (1D) patterns. All of the polymer films exhibited out-of-plane (h00) signals corresponding to side-chain interdigitation. We also observed obvious (010) in-plane peaks corresponding to periodic π-stacking between the two facing conjugated backbones. These in-plane (010) peaks revealed that the polymers adopted predominately edge-on orientations with the backbone plane approximately perpendicular to the substrate. The out-of-plane lamellar spacing (*d*_l_) and the in-plane π-stacking spacing (*d*_π_) of the polymers were estimated using the Bragg equation (Table 6). The π-stacking spacing of the poly(bichalcogenophene)s increased upon increasing the tellurophene content (*d*_π_: 3.84 Å for **PSSe**, 3.87 Å for **PSeTe**, and 3.94 Å for **PSTe**), presumably because of the larger size of the Te atom. In contrast, the lamellar spacing decreased upon increasing the tellurophene content (*d*_l_: 15.95 Å for **PSTe**, 16.33 Å for **PSeTe**, and 16.51 Å for **PSSe**). It is likely that the increased π-stacking distance provided more volume for the hexyl groups to interdigitate, thereby contracting the lamellar spacing.^70^ Nevertheless, it is interesting that the poly(terchalcogenophene) **PSSeTe**, featuring the lowest tellurophene content (one third), possessed the shortest π-stacking spacing (3.82 Å) and, thus, the largest lamellar spacing (17.18 Å). After thermal annealing at 100 °C for 30 min, the diffraction patterns were essentially unchanged, but with enhanced signal intensities and slightly lower values of *d*_π_ and *d*_l_. Interestingly, **PSeTe** exhibited two lamellar interlayer spacings (16.33 and 12.95 Å), indicating the formation of two crystal phases with different types of side-chain interdigitation.^52,71^

**Table 6:** Lamellar spacings and π-stacking spacings of the polymers

	As-cast		Thermal annealing at 100 °C	
	Lamellar spacing (Å)	π-stacking spacing (Å)	Lamellar spacing (Å)	π-stacking spacing (Å)
**PSSe**	16.51	3.84	16.51	3.83
**PSTe**	15.95	3.94	15.92	3.92
**PSeTe**	16.33, 12.95	3.87	16.28, 12.80	3.87
**PSSeTe**	17.18	3.82	16.55	3.82

### OFET devices

To evaluate the p-type mobilities of **PSSe**, **PSTe**, **PSeTe**, and **PSSeTe**, we fabricated OFET devices incorporating ODTS-treated SiO_2_/Si dielectric substrates in a bottom-gate/top-contact configuration. The spin-cast polymer thin films were thermally annealed at 100 °C for 10 min. When the applied gate-to-source voltage (*V*_GS_) was ramped from 0 to −75 V in steps of −15 V, the OFETs exhibited typical p-type behavior (Fig. S7†). Table 7 summarizes the saturation organic field-effect mobilities (*μ*_sat_), threshold voltages (*V*_th_), and current on/off ratios (*I*_on_/*I*_off_) calculated from the transfer characteristics (Fig. S8†) of these devices. The edge-on π-stacking orientations of the polymers were favorable for horizontal carrier mobility, because the direction of π-stacking was the same as the direction of current flow. The mobility of the poly(bichalcogenophene)s decreased when incorporating the two heavier chalcogenophenes: 7.2 × 10^−3^ cm^2^ V^−1^ s^−1^ for **PSSe** > 2.7 × 10^−3^ cm^2^ V^−1^ s^−1^ for **PSTe** > 5.2 × 10^−4^ cm^2^ V^−1^ s^−1^ for **PSeTe**. Because of the larger radius of the Te atom, **PSTe** and **PSeTe** possessed larger π-stacking spacings, which were presumably related to their lower mobilities. Notably, however, the tellurophene-containing polymers had relatively poorer solubilities, which might also have influenced their film quality and, thus, mobility. Interestingly, the poly(terchalcogenophene) **PSSeTe** exhibited the highest mobility (1.2 × 10^−2^ cm^2^ V^−1^ s^−1^), consistent with it having the shortest π-stacking distance. This mobility is one of the highest ever reported for a tellurophene-chalcogenophene-based polymer.^70^

**Table 7:** Charge carrier mobilities, threshold voltages, and on/off ratios for OFETs incorporating spin-coated **PSSe**, **PSTe**, **PSeTe**, and **PSSeTe** films

Polymer	*I*_on_/I_off_	*V*_th_ (V)	Mobility (cm^2^ V^−1^ s^−1^)
**PSSe**	8.5 × 10^3^	−7.5	7.2 × 10^−3^
**PSTe**	8.1 × 10^2^	−3.6	2.7 × 10^−3^
**PSeTe**	4.9 × 10^3^	−19.6	5.2 × 10^−4^
**PSSeTe**	8.0 × 10^3^	−23.1	1.2 × 10^−2^

## Conclusions

We have developed a new class of side-chain regioregular and main-chain alternating polychalcogenophenes having precisely controlled sequences. Our success in obtaining these highly regular polychalcogenophenes relied on the synthesis of corresponding unsymmetrical monomers: the diiodobichalcogenophenes **SSeI2**, **STeI2**, and **SeTeI2** and the diiodoterchalcogenophene **SSeTeI2** (in model A). Because of greater electron-deficiency and less steric hindrance, Grignard metathesis of the monomers **SSeI2**, **STeI2**, and **SeTeI2** occurred regioselectively at the lighter chalcogenophene unit (*i.e.*, the thiophene units for **SSeI2**, **STeI2**, and **SSeTeI2**; the selenophene unit for **SeTeI2**). All of the regioselectively prepared Mg species underwent Ni(dppp)Cl_2_-catalyzed KCTP. The key step during the KCTP was the Ni complex undergoing thermodynamically favorable ring-walking from the lighter to the heavier chalcogenophene, with corresponding higher complexation energy with the Ni catalyst. The ring-walking facilitated subsequent oxidative addition, suppressing the possibility of chain-transfer or chain-termination. Our first synthesis of an ABC-type periodic poly(terchalcogenophene) **PSSeTe** also reveals that the Ni catalyst could walk efficiently over a distance of three rings (*ca.* 1 nm) from a thiophene unit *via* a selenophene unit to a tellurophene unit, all with a gradual increase in complexation energy, thereby preserving the mechanism of catalyst-transfer polycondensation. The thin-film morphologies and the optical, electrochemical, conformational, and OFET properties of the polymers could be tailored systematically by combining the different chalcogenophenes at various ratios—a promising feature for a wide range of potential applications. This paper provides a design concept for the use of single unsymmetrical monomers to create new AB- and ABC-type alternating and periodic conjugated polymers with high regioregularity.

## Conflicts of interest

There are no conflicts to declare.

## Supplementary Material

SC-011-D0SC00404A-s001
