# Supplementary material for: Synthesis of side-chain regioregular and main-chain alternating poly(bichalcogenophene)s and an ABC-type periodic poly(terchalcogenophene)
Source: Chem Sci. 2020 Mar 10;11(15):3836–44. doi: 10.1039/d0sc00404a (PMC8152668; doi:10.1039/d0sc00404a)
Supplement: SC-011-D0SC00404A-s001 [file SC-011-D0SC00404A-s001.pdf]

Electronic Supplementary Material (ESI) for Chemical Science.
This journal is © The Royal Society of Chemistry 2020

Supporting Information

Synthesis of Side-Chain Regioregular and Main-Chain Alternating Poly(bichalcogenophenes) and an ABC-Type Periodic Poly(terchalcogenophene)

Huai-Hsuan Liu[a], Wei-Wei Liang[a], Yu-Ying Lai[c], Yen-Chen Su[a], Hau-Ren Yang[c], Kuang-Yi Cheng[a], Sheng-Cih Huang[a] and Yen-Ju Cheng[ab]*

[a]Department of Applied Chemistry, National Chiao Tung University, 1001 University Road, Hsinchu, 30010, Taiwan

[b]Center for Emergent Functional Matter Science, National Chiao Tung University, 1001 University Road, Hsinchu, 30010, Taiwan

[c]Institute of Polymer Science and Engineering, National Taiwan University, No.1, Sec. 4, Roosevelt Rd., Taipei 10617, Taiwan

Email: yjcheng@nctu.edu.tw

Table of Contents

Experimental procedures 2

Synthetic Procedures....4

NMR Spectra of Intermediates and Polymers 14

Procedure for M_n versus Monomer Conversion Plot 16

MALDI-TOF Mass Spectrometry of PSSeTe 17

Characteristics of OFETs....17

NMR Spectra 19

GPC Traces....47

DFT Computation and Analysis 49

References....56

Experimental Procedures

General measurement and characterization. UV data were collected by the HITACHI U-4100 spectrophotometer. The electrochemical cyclic voltammetry (CV) was conducted on a CH Instruments Model 611D. A carbon glass coated with a thin polymer film was used as the working electrode and Ag/Ag^{+} electrode as the reference electrode, while 0.1 M tetrabutylammonium hexafluorophosphate (Bu_4NPF_6) in acetonitrile was the electrolyte. CV curves were calibrated using ferrocence as the standard, whose HOMO is set at −4.8 eV with respect to zero vacuum level. The HOMO energy levels were obtained from the equation HOMO = $-(E_{ox}^{onset} - E_{(ferrocene)}^{onset} + 4.8)$ eV. The LUMO levels were obtained from the equation LUMO = $-(E_{red}^{onset} - E_{(ferrocene)}^{onset} + 4.8)$ eV. High resolution mass spectra (HRMS) were obtained on a JEOL AccuTOF GCX (EI). MALDI spectra were recorded on an Bruker Autoflex III system using trans-2-[3-(4-tert-butylphenyl)-2-methyl-2-propenylidene]malononitrile (DCTB) as a matrix in THF (5 mg/mL). GIXRD measurements were performed at the BL23A station of the National Synchrotron Radiation Research Center (NSRRC), Taiwan. Polymer molecular weights were determined with a HLC-8321GPC/HT (1,2,4-trichlorobenzene, 140 °C, 1 mL/min flow rate) using Tosoh Bioscience LLC TSKgel GMHHR-HHT2 mixed-bed columns and narrow molecular weight distribution polystyrene standards.

Film preparation for GIWAXRD. The wafers with thermal grown 300 nm SiO_2 were ultrasonically cleaned in sequential detergent, water, acetone, isopropyl alcohol and dried under nitrogen purging, followed by UV/Ozone treatment for 20 min. The polychalcogenophene thin films were prepared from 20 mg/mL solutions in *o*DCB on 1.4 × 1.4 cm silicon wafers. The post-annealing of the thin films was carried out in the glovebox to prevent oxidation.

OFET fabrication. A n-type heavily doped Si wafer with a SiO_2 layer of 300 nm and a capacitance of 11.5 nF cm^{-2} as the gate electrode and dielectric layer was ultrasonically cleaned sequentially in detergent, water and isopropyl alcohol. Octadecyltrichlorosilane (ODTS) was used as a self-assembled monolayer. The polychalcogenophene samples were

prepared form 20 mg/mL solutions in chloroform and were spin-coated on the ODTS-treated silicon wafers. The gold source and drain electrodes (40 nm in thickness) were then deposited on the organic layer by vacuum evaporation through a shadow mask, affording a bottom-gate, top-contact device configuration. OFET measurement was carried out at room temperature under a nitrogen atmosphere using an Agilent Technologies 4156C instrument. The mobility calculation was based on the equation I_{ds} (W/2L)μCi(Vg - Vt)^2 in the saturation regime, where I_{ds} is the drain–source current, W is the channel width (1 mm), L is the channel length (100 mm), μ is the field-effect mobility, Ci is the capacitance per unit area of the dielectric layer, V_g is the gate voltage, and V_t is the threshold voltage.

Synthetic Procedures

All chemicals were purchased from Aldrich, Acros or TCI and used as received unless specified. ^{1}H and ^{13}C NMR spectra were obtained in deuterium-substituted chloroform by Varian 400 MHz spectrometers and 0.5 wt% TMS also used as reference. 3-hexyltellurophene[1], compound 1[2], compound 2[3], compound 3[4] and compound SeS[5] were synthesized as reported.

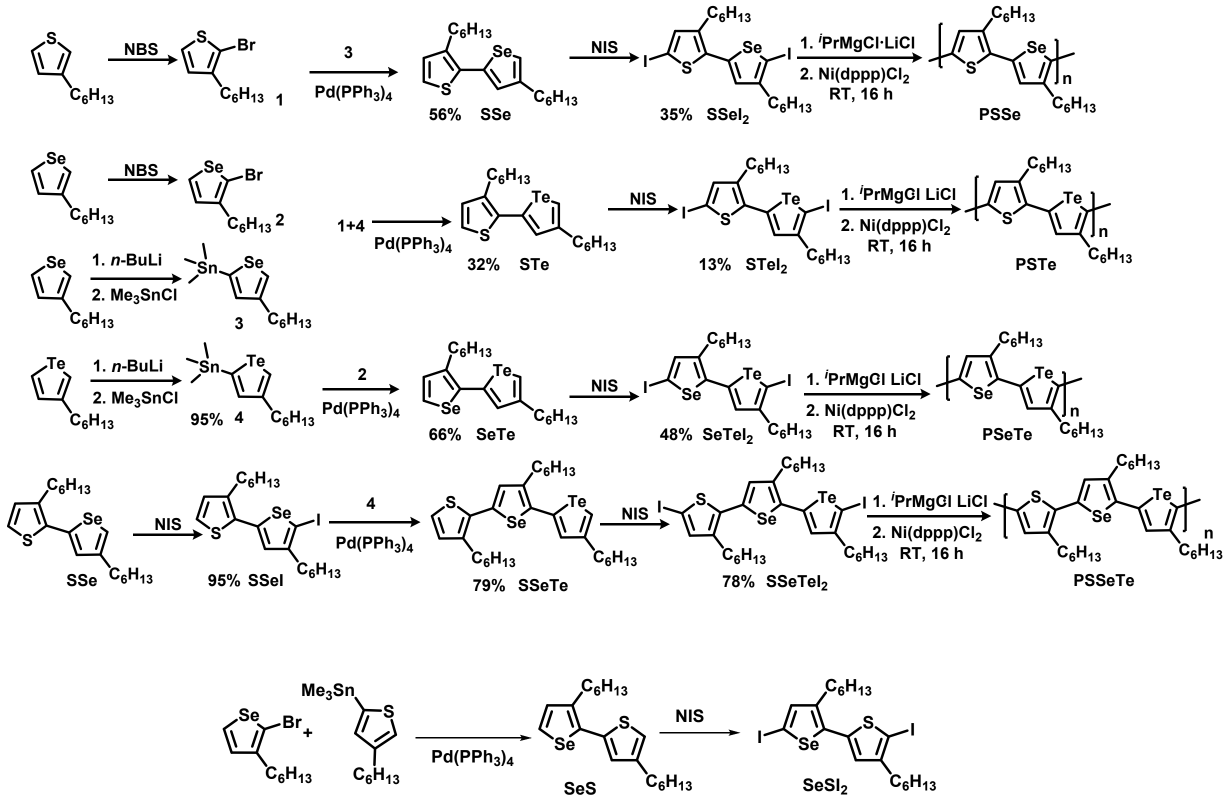

Scheme S1. Synthetic routes for **SSeI₂**, **SeSI₂**, **STeI₂**, **SeTeI₂**, and **SSeTeI₂** monomers and their polymers.

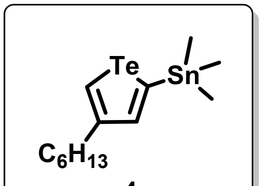

4

Synthesis of 2-bromo-3-hexylselenophene (4). To a solution of 3-hexyltellurophene (2.5

g, 9.40 mmol) in anhydrous THF (40 mL) was added a solution of 2.5 M *n*-BuLi (3.95 mL, 9.87 mmol) dropwisely under 0 °C for 1 h. Then a solution of 1 M trimethyltin chloride (9.4 mL, 9.40 mmol) was added. The reaction mixture was stirred at room temperature for 16 h then extracted with dichloromethane and water. The organic layer was collected and dried with $MgSO_4$. After removal of the solvent under reduced pressure, a crude brown oil was obtain (3.84 g, 95 %), the crude compound was used for further reaction without purification.

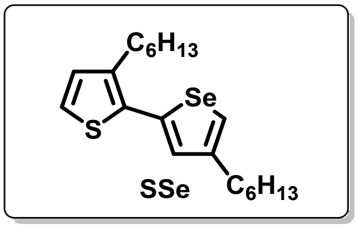

Synthesis of 3-hexyl-2-(4-hexylselenophen-2-yl)thiophene (SSe). A mixture of (4-hexylselenophen-2-yl)trimethylstannane **3** (2 g, 5.26 mmol), 2-bromo-3-hexylthiophene **1** (1.29 g, 5.26 mmol), tetrakis(triphenylphosphine)palladium (121 mg, 0.11 mmol) in toluene (50 mL) under N_2 was stirred at 120 °C for 16 h. After removal of the solvent under reduced pressure, the residue was purified by silica gel chromatography with hexane as the eluent to give a yellow oil (1.12 g, 56 %). ^{1}H NMR (400 MHz, $CDCl_3$, δ ppm): 0.87-0.92 (m, 6H), 1.28-1.40 (m, 12H), 1.59-1.67 (m, 4H), 2.58 (t, 2H, *J* = 7.6 Hz), 2.73 (t, 2H, *J* = 8.0 Hz), 6.92 (d, 1H, *J* = 5.2 Hz), 7.13 (d, 1H, *J* = 5.6 Hz), 7.14 (s, 1H), 7.50 (d, 1H, *J* = 1.2 Hz). ^{13}C NMR ($CDCl_3$, 100 MHz, δ ppm): 14.0, 14.1, 22.6, 29.0, 29.1, 29.2, 30.2, 30.7, 31.6, 31.7, 32.2, 123.3, 124.3, 129.9, 130.0, 133.3, 139.0, 140.1, 145.4. HRMS ($C_{20}H_{30}SSe$): calcd, 382.1228; found (EI^+), 382.1228.

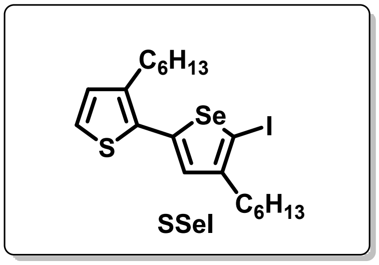

Synthesis of 3-hexyl-2-(4-hexyl-5-iodoselenophen-2-yl) thiophene (SSeI). *N*-iodosuccinimide (0.89 g, 3.9 mmol) was added in portions to a solution of **SSe** (1 g, 2.6 mmol) in DMF (50 mL). The mixture was stirred at room temperature for 3 h. The mixture was quenched in $Na_2S_2O_3$ aqueous solution, followed by the extraction with dichloromethane and water. The organic layer was collected and dried with $MgSO_4$. After removal of the solvent under reduced pressure, the residue was purified by silica gel chromatography with hexane as the eluent to give a brown oil (1.27 g, 95%). ^{1}H NMR (400 MHz, $CDCl_3$, δ ppm): 0.87-0.90 (m, 6H), 1.29-1.37 (m, 12H), 1.54-1.59 (m, 4H), 2.50 (t, 2H, J = 7.8 Hz), 2.68 (t, 2H, J = 8.0 Hz), 6.903 (d, 1H, J = 5.2 Hz), 6.905 (s, 1H), 7.15 (d, 1H, J = 5.2 Hz). ^{13}C NMR ($CDCl_3$, 100 MHz, δ ppm): 14.0, 14.1, 22.6, 28.9, 29.2, 29.8, 30.7, 31.6, 34.0, 75.5,123.9, 129.0, 130.0, 132.6, 139.7, 145.0, 149.5. HRMS ($C_{20}H_{29}ISSe$): calcd, 508.0194; found (EI^+), 508.0171.

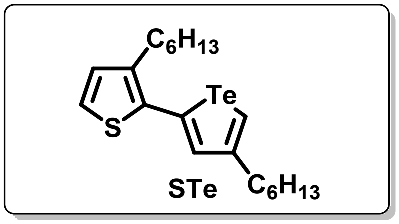

Synthesis of 3-hexyl-2-(4-hexyltellurophen-2-yl)thiophene (STe). A mixture of (4-hexyltellurophen-2-yl)trimethylstannane **4** (2.5 g, 5.81 mmol), 2-bromo-3-hexylthiophene **1** (1.43 g, 5.81 mmol), tetrakis(triphenylphosphine)palladium (134 mg, 0.12 mmol) in toluene (55 mL) under N_2 was stirred at 120 ^oC for 16 h. After removal of the solvent under reduced pressure, the residue was purified by silica gel chromatography with hexane as the eluent to give a yellow oil (0.8 g, 32 %). ^{1}H NMR (400 MHz, $CDCl_3$, δ ppm): 0.90-0.94 (m, 6H), 1.29-1.42 (m, 12H), 1.61-1.67 (m, 4H), 2.61 (t, 2H, J = 7.6 Hz), 2.71 (t, 2H, J = 8.0 Hz), 6.92 (d, 1H, J = 5.2 Hz), 7.09 (d, 1H, J = 5.2 Hz), 7.59 (d, 1H, J = 1.2 Hz), 8.31 (d, 1H, J = 0.8 Hz). ^{13}C NMR ($CDCl_3$, 100 MHz, δ ppm): 14.1, 14.1, 22.6, 29.0, 29.3, 29.4, 30.3, 30.6, 31.6, 31.7, 35.2, 118.9, 122.9, 130.1, 134.9, 137.6, 137.9, 138.1, 152.4. HRMS ($C_{20}H_{30}STe$): calcd, 432.1125; found (EI^+), 432.1122.

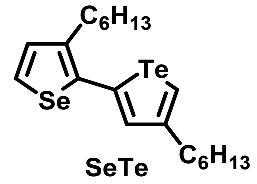

Synthesis of 3-hexyl-2-(4-hexyltellurophen-2-yl)selenophene (SeTe). A mixture of (4-hexyltellurophen-2-yl)trimethylstannane **4** (2.5 g, 5.81 mmol), 2-bromo-3-hexylselenophene **2** (1.71g, 5.81 mmol), 2-bromo-3-hexylthiophene (1.43 g, 5.81 mmol), tetrakis(triphenylphosphine)palladium (134 mg, 0.12 mmol) in toluene (55 mL) under N_2 was stirred at 120 °C for 16 h. After removal of the solvent under reduced pressure, the residue was purified by silica gel chromatography with hexane as the eluent to give a yellow oil (1.84 g, 66 %). ^{1}H NMR (400 MHz, $CDCl_3$, δ ppm): 0.86-0.90 (m, 6H), 1.29-1.37 (m, 12H), 1.59-1.63 (m, 4H), 2.58 (m, 2H), 2.67 (t, 2H, *J* = 8.0 Hz), 7.19 (d, 1H, *J* = 6.0 Hz), 7.51 (d, 1H, *J* = 1.6 Hz), 7.74 (d, 1H, *J* = 5.6 Hz), 8.31 (t, 1H, *J* = 0.8 Hz). ^{13}C NMR ($CDCl_3$, 100 MHz, δ ppm): 14.0, 14.1, 22.6, 29.0, 29.3, 30.2, 30.4, 30.6, 31.6, 31.7, 35.2, 119.2, 127.8, 133.6, 137.0, 138.6, 139.8, 142.4, 152.2. HRMS ($C_{20}H_{30}SeTe$): calcd, 480.0569; found (EI$^+$), 480.0559.

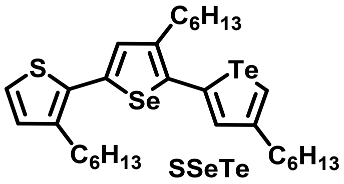

Synthesis of 3-hexyl-2-(4-hexyl-5-(4-hexyltellurophen-2-yl)selenophen-2-yl)thiophene (SSeTe). A mixture of (4-hexyltellurophen-2-yl)trimethylstannane (92.4 g, 0.217 mmol), SSeI (100 mg, 0.197 mmol), and tetrakis(triphenylphosphine)palladium(0) (11.4 mg, 0.01 mmol) in toluene (1.9 mL) under N_2 was stirred at 120 °C for 16 h. After removal of the solvent under reduced pressure, the residue was purified by silica gel chromatography with hexane as the eluent to give a yellow oil (127 mg, 79%). ^{1}H NMR (400 MHz, $CDCl_3$, *δ* ppm): 0.86-0.90 (m, 9H), 1.29-1.40 (m, 18H), 1.60-1.66 (m, 6H), 2.58 (t, 2H, *J* = 7.6 Hz), 2.66 (t, 2H, *J* = 7.8 Hz), 2.75 (t, 2H, *J* = 7.8 Hz), 6.91 (d, 1H, *J* = 5.2 Hz),7.130 (s, 1H),

7.131 (d, 1H, J = 5.2 Hz), 7.52 (s, 1H), 8.32 (s, 1H), ^{13}C NMR ($CDCl_3$, 100 MHz, δ ppm): 14.1, 22.6,29.0, 29.3, 29.4, 29.7, 30.3, 30.5, 30.6, 30.7, 31.7, 35.2, 119.4, 123.4, 130.2, 131.8, 133.0, 136.3, 137.7, 138.3, 139.3, 140.1, 142.5, 152.3. HRMS ($C_{30}H_{44}SSeTe$): calcd, 646.1386; found (EI^+), 646.1411.

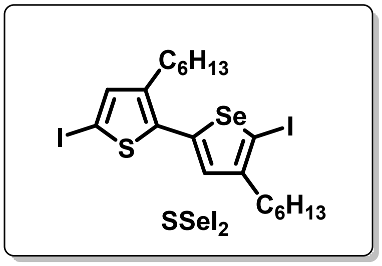

Synthesis of 3-hexyl-2-(4-hexyl-5-iodoselenophen-2-yl)-5-iodothiophene ($SSeI_2$). To a solution of **SSe** (1 g, 2.62 mmol) and *p*-toluenesulfonic acid (1g, 5.24 mmol) in CH_2Cl_2 (30 mL) was added *N*-iodosuccinimide (1.3 g, 5.76 mmol) in portions. The reaction was stirred at room temperature for 3 h. The mixture was then quenched in $Na_2S_2O_3$ aqueous solution, and followed by the extraction with dichloromethane and water. The organic layer was collected and dried with $MgSO_4$. After removal of the solvent under reduced pressure, the residue was purified by silica gel chromatography with hexane as the eluent to give a brown oil (0.58 g, 35 %). ^{1}H NMR (400 MHz, $CDCl_3$, δ ppm): 0.87-0.90 (m, 6H), 1.29-1.37 (m, 12H), 1.54-1.59 (m, 4H), 2.50 (t, 2H, J = 7.8 Hz), 2.63 (t, 2H, J = 7.8 Hz), 6.85 (s, 1H), 7.04 (s, 1H). ^{13}C NMR ($CDCl_3$, 100 MHz, δ ppm): 14.0, 14.1, 22.6, 28.9, 29.1, 29.8, 30.6, 31.5, 31.6, 33.9, 72.0, 76.4, 129.5, 138.6, 139.7, 141.5, 143.3, 149.6. HRMS ($C_{20}H_{28}I_2SSe$): calcd, 633.9161; found (EI^+), 633.9187.

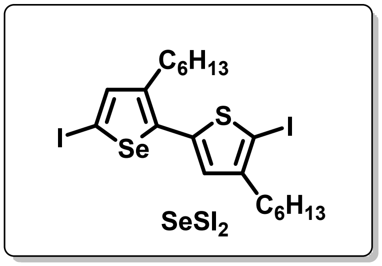

Synthesis of 3-hexyl-2-(4-hexyl-5-iodothiophen-2-yl)-5-iodoselenoophene ($SeSI_2$). To a solution of **SeS** (300 mg, 0.787 mmol) and *p*-toluenesulfonic acid (299 mg, 1.57 mmol) in

CH_2Cl_2 (15 mL), *N*-iodosuccinimide (265 mg, 1.18 mmol) was added in portions, the reaction was stirred at room temperature for 3 h. The mixture was then quenched in $Na_2S_2O_3$ aqueous solution, and followed by the extraction with dichloromethane and water. The organic layer was collected and dried with $MgSO_4$. After removal of the solvent under reduced pressure, the residue was purified by silica gel chromatography with hexane as the eluent to give a brown oil (230 mg, 46 %). ^{1}H NMR (400 MHz, $CDCl_3$, δ ppm): 0.87-0.90 (m, 6H), 1.29-1.37 (m, 12H), 1.54-1.59 (m, 4H), 2.51 (t, 2H, *J* = 7.8 Hz), 2.62 (t, 2H, *J* = 7.8 Hz), 6.63 (s, 1H), 7.37 (s, 1H). ^{13}C NMR ($CDCl_3$, 100 MHz, δ ppm): 14.1, 22.5, 22.6, 28.9, 29.1, 29.7, 29.8, 29.9, 30.7, 31.5, 31.6, 32.3, 73.8, 74.8, 127.3, 140.5, 141.5, 143.4, 143.5, 147.6. HRMS ($C_{20}H_{28}I_2SSe$): calcd, 633.9161; found (EI^+), 633.9159.

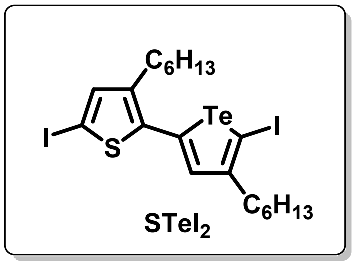

Synthesis of 3-hexyl-2-(4-hexyl-5-iodotellurophen-2-yl)-5-iodothiophene ($STeI_2$). To a solution of **STe** (1 g, 2.31 mmol) and *p*-toluenesulfonic acid (0.88g, 4.62 mmol) in CH_2Cl_2 (25 mL) was added *N*-iodosuccinimide (1.3 g, 5.08 mmol) in portions. The reaction was stirred at room temperature for 3 h. The mixture was then quenched in $Na_2S_2O_3$ aqueous solution, and followed by the extraction with dichloromethane and water. The organic layer was collected and dried with $MgSO_4$. After removal of the solvent under reduced pressure, the residue was purified by silica gel chromatography with hexane as the eluent to give a brown oil (0.21 g, 13%). ^{1}H NMR (400 MHz, $CDCl_3$, δ ppm): 0.87-0.90 (m, 6H), 1.26-1.36 (m, 12H), 1.55-1.59 (m, 4H), 2.51-2.59 (m, 2H), 7.03 (s, 1H), 7.20 (s, 1H). ^{13}C NMR ($CDCl_3$, 100 MHz, δ ppm): 14.0, 14.1, 22.5, 22.6, 28.9, 29.0, 29.2, 29.8, 30.6, 31.5, 31.6, 36.5, 67.8, 71.6, 136.5, 138.0, 139.8, 140.6, 143.0, 155.4. HRMS ($C_{20}H_{28}I_2STe$): calcd, 683.9058; found (EI^+), 683.9062.

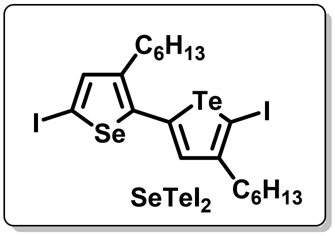

Synthesis of 3-hexyl-2-(4-hexyl-5-iodotellurophen-2-yl)-5-iodoselenophene ($SeTeI_2$). *N*-iodosuccinimide (1.3 g, 4.58 mmol) was added to a solution of **SeTe** (1 g, 2.08 mmol) and *p*-toluenesulfonic acid (0.79g, 4.16 mmol) in CH_2Cl_2 (25 mL) in portions. The reaction was stirred at room temperature for 3 h. The mixture was then quenched in $Na_2S_2O_3$ aqueous solution, and followed by the extraction with dichloromethane and water. The organic layer was collected and dried with $MgSO_4$. After removal of the solvent under reduced pressure, the residue was purified by silica gel chromatography with hexane as the eluent to give a brown oil (0.73 g, 48 %). ^1H NMR (400 MHz, $CDCl_3$, δ ppm): 0.87-0.91 (m, 6H), 1.30-1.35 (m, 12H), 1.53-1.57 (m, 4H), 2.52 (t, 2H, J = 8.0 Hz), 2.55 (t, 2H, J = 8.0 Hz), 7.13 (s, 1H), 7.36 (s, 1H). ^{13}C NMR ($CDCl_3$, 100 MHz, δ ppm): 14.0, 14.1, 22.5, 22.6, 28.9, 29.2, 29.8, 30.0, 30.1, 31.5, 31.6, 36.5, 68.1, 73.3, 137.1, 140.2, 142.2, 143.8, 147.8, 155.3. HRMS ($C_{20}H_{28}I_2TeSe$): calcd, 731.8502; found (EI^+), 731.8507.

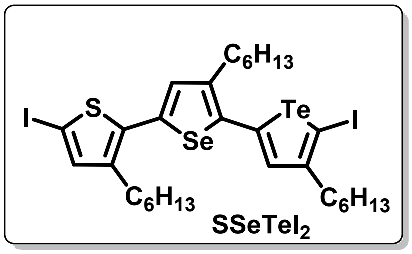

Synthesis of 3-hexyl-2-(4-hexyl-5-(4-hexyl-5-iodohexyltellurophen-2-yl)selenophen-2-yl)-5-iodothiophene ($SSeTeI_2$). To a solution of **SSeTe** (0.5 g, 0.78 mmol), *p*-toluenesulfonic acid (0.29 g, 1.56 mmol) in CH_2Cl_2 (25 mL) was added *N*-iodosuccinimide (0.44 g, 1.94 mmol) in portions. The mixture was stirred at room temperature for 3 h. The mixture was then quenched in $Na_2S_2O_3$ aqueous solution, followed by the extraction with dichloromethane and water. The organic layer was collected and dried with $MgSO_4$. After removal of the solvent under reduced pressure, the residue was purified by silica gel chromatography with hexane as the eluent to give a yellow solid (0.54 g, 78%). ^1H NMR

(400 MHz, $CDCl_3$, δ ppm): 0.87-0.91 (m, 9H), 1.30-1.36 (m, 18H), 1.55-1.60 (m, 6H), 2.53 (t, 2H, J = 8.0 Hz), 2.57 (t, 2H, J = 8.0 Hz), 2.68 (t, 2H, J = 8.0 Hz), 7.04 (s, 1H), 7.05 (s, 1H), 7.21 (s, 1H). ^{13}C NMR ($CDCl_3$, 100 MHz, δ ppm): 14.1, 22.61, 22.64, 29.0, 29.1, 29.2, 29.3, 29.7, 29.9, 30.59, 30.63, 31.63, 31.66, 31.68, 36.5, 67.3, 71.7, 132.1, 136.5, 136.6, 138.8, 139.9, 140.76, 140.79, 141.2, 142.5, 155.3. HRMS ($C_{30}H_{42}I_2STeSe$): calcd, 897.9319; found (EI^+), 897.9306.

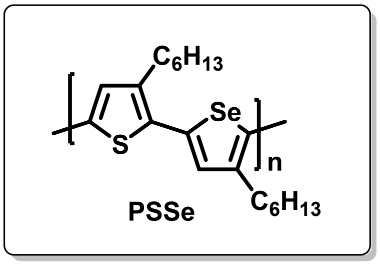

Synthesis of poly(3-hexylselenophene-*alt*-3-hexylthiophene) (PSSe). To a solution of 1.3 M isopropylmagnesium chloride lithium chloride complex (0.25 mL, 0.32 mmol) diluted by dried THF (8 mL) was added dropwisely a solution of **$SSeI_2$** (200 mg, 0.32 mmol) in dried THF (8 mL) in a glovebox. After Grignard metathesis was completed (monitored by thin layer chromatography), [1,3-bis(diphenylphosphino)propane]dichloronickel(II) (1.7 mg, 0.0032 mmol) was added. The reaction was stirred at room temperature for 16 h. After **PSSe** was precipitated with 6 M HCl/MeOH solution and washed with methanol, a deep purple solid was yielded (43 mg, 36 %). ^1H NMR (400 MHz, $CDCl_3$, δ ppm): 0.91 (br, 6H), 1.25-1.43 (br, 12H), 1.68-1.70 (br, 4H), 2.76 (br, 4H), 6.92 (s, 1H), 7.19 (s, 1H). ^{13}C NMR ($CDCl_3$, 100 MHz, δ ppm): 13.90, 13.92, 22.5, 29.1, 29.2, 29.5, 30.39, 30.44, 30.5, 30.6, 31.6, 129.1, 131.6, 133.2, 135.2, 135.8, 138.0, 139.7, 141.6.

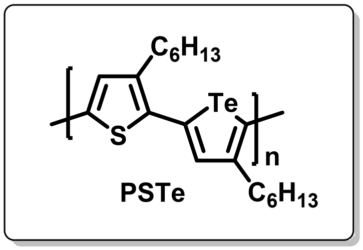

Synthesis of poly(3-hexyltellurophene-*alt*-3-hexylthiophene) (PSTe). To a solution of 1.3 M isopropylmagnesium chloride lithium chloride complex (0.22 mL, 0.29 mmol) diluted by dried THF (8 mL) was added dropwisely a solution of **STeI$_2$** (200 mg, 0.29 mmol) in dried THF (8 mL) in a glovebox. After Grignard metathesis was completed (monitored by thin layer chromatography), [1,3-bis(diphenylphosphino)propane]dichloronickel(II) (1.6 mg, 0.0029 mmol) was added. The reaction was stirred at room temperature for 16 h. After **PSTe** was precipitated with 6 M HCl/MeOH solution and washed with methanol, a deep purple solid was yielded (56 mg, 44 %). ^{1}H NMR (400 MHz, CDCl$_3$, δ ppm): 0.90-0.93 (br, 6H), 1.34-1.42 (br, 12H), 1.66-1.68 (br, 4H), 2.65-2.67 (br, 2H), 2.69-2.78 (br, 2H), 6.82 (s, 1H), 7.65 (s, 1H). ^{13}C NMR (CDCl$_3$, 100 MHz, *δ* ppm): 14.0, 14.1, 22.7, 29.3, 29.4, 29.8, 30.6, 30.8, 31.75, 31.77, 32.5, 129.5, 131.4, 133.0, 137.7, 139.2, 139.7, 139.8, 148.2.

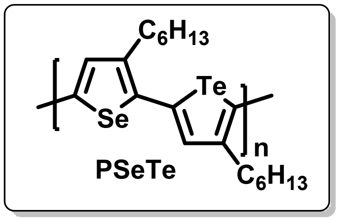

Synthesis of poly(3-hexyltellurophene-*alt*-3-hexylselenophene) (PSeTe). To a solution of 1.3 M isopropylmagnesium chloride lithium chloride complex (0.21 mL, 0.27 mmol) diluted by dried THF (8 mL) was added dropwisely a solution of **SeTeI$_2$** (200 mg, 0.27 mmol) in dried THF (8 mL) in a glovebox. After Grignard metathesis was completed (monitored by thin layer chromatography), [1,3-bis(diphenylphosphino)propane]dichloronickel(II) (1.4 mg, 0.0027 mmol) was added. The reaction was stirred at room temperature for 16 h. After **PSeTe** was precipitated with 6 M HCl/MeOH solution and washed with methanol, a deep purple solid was yielded (70 mg, 53 %). ^{1}H NMR (400 MHz, CDCl$_3$, δ ppm): 0.92-0.95 (br, 6H), 1.27-1.44 (br, 12H), 1.67 (br, 4H), 2.63 (br, 2H), 2.74 (br, 2H), 6.97 (s, 1H), 7.54 (s, 1H). ^{13}C NMR (CDCl$_3$, 100 MHz, *δ* ppm): 14.05, 14.06, 22.7, 29.0, 29.3, 29.4, 30.3, 30.61, 30.63, 30.7, 30.87, 30.91, 31.76, 31.77, 32.5, 35.3, 132.9, 134.6, 134.8, 140.5, 140.8, 143.0, 143.9, 147.7.

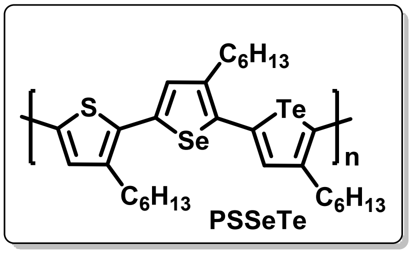

Synthesis of ABC-type periodic terpolymer, poly(3-hexyltellurophene-*per*-3-hexylselenophene-*per*-3-hexylthiophene) (PSSeTe). To a solution of 1.3 M isopropylmagnesium chloride lithium chloride complex (0.17 mL, 0.22 mmol) diluted by dried THF (8 mL) was added dropwisely a solution of **SSeTeI$_2$** (200 mg, 0.22 mmol) in dried THF (8 mL) in a glovebox. After Grignard metathesis was completed (monitored by thin layer chromatography), [1,3-bis(diphenylphosphino)propane]dichloronickel(II) (1.2 mg, 0.0022 mmol) was added. The reaction was stirred at room temperature for 16 h. After **PSSeTe** was precipitated with 6 M HCl/MeOH solution and washed with methanol, a deep purple solid was yielded (110 mg, 77 %). ^{1}H NMR (400 MHz, CDCl$_3$, δ ppm): 0.92-0.95 (br, 9H), 1.27-1.44 (br, 18H), 1.67 (br, 6H), 2.69 (br, 2H), 2.77 (br, 4H), 6.83 (s, 1H), 7.17 (s, 1H), 7.58 (s, 1H). ^{13}C NMR (CDCl$_3$, 100 MHz, δ ppm): 14.05, 14.06, 22.68, 22.69, 29.0, 29.31, 29.34, 29.4, 29.7, 30.3, 30.6, 30.7, 30.8, 31.76, 31.77, 31.78, 32.5, 35.3, 129.5, 131.8, 131.9, 133.3, 135.0, 137.6, 140.0, 140.1, 140.5, 140.8, 142.5, 148.1.

NMR Spectra of Intermediates and Polymers

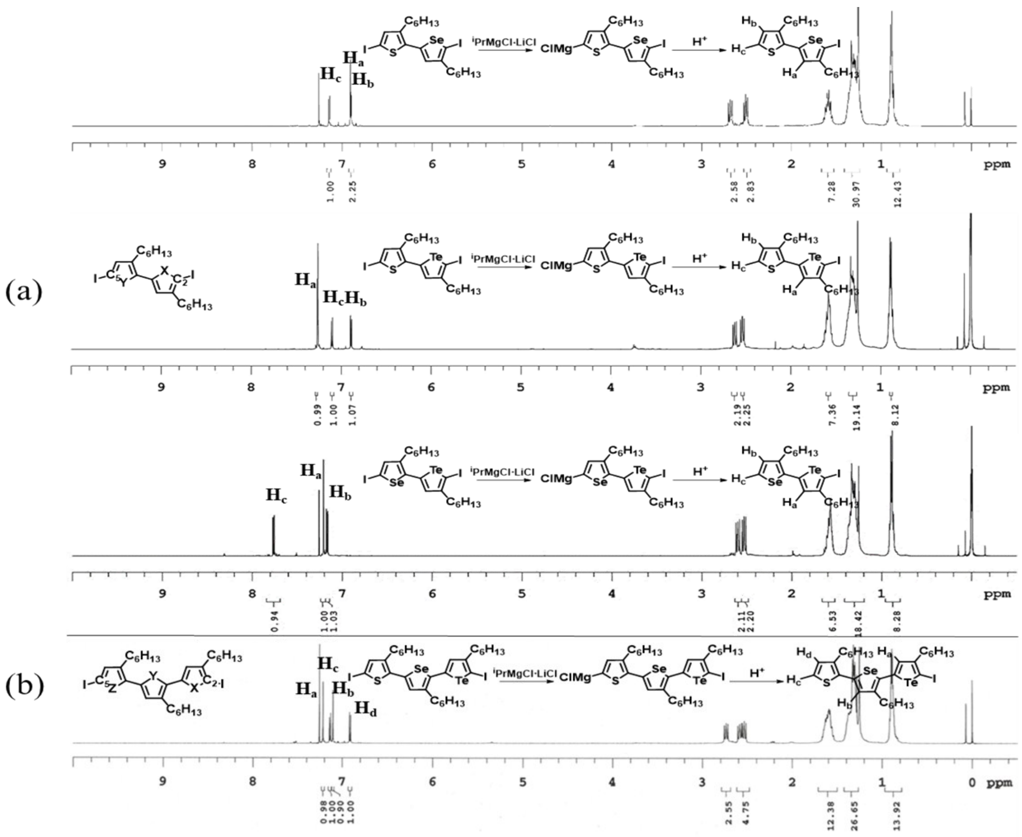

Figure S1. ^{1}H NMR spectra of the crude products from Grignard metathesis/acidic quenching of (a) **SSeI$_2$**, **STeI$_2$**, and **SeTeI$_2$** and (b) **SSeTeI$_2$**.

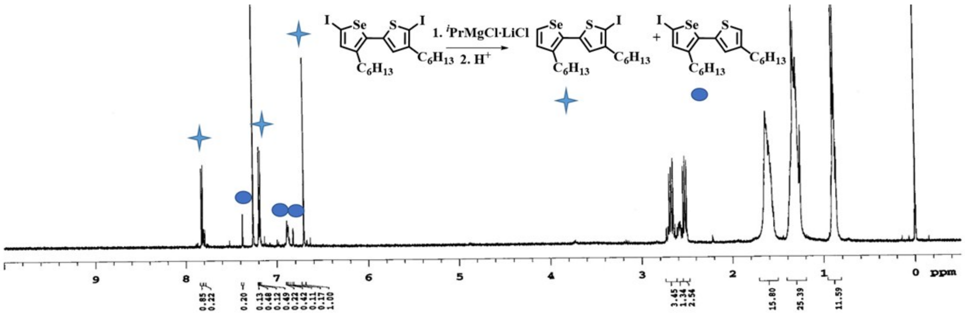

Figure S2. Crude ^{1}H-NMR spectrum for Grignard metathesis of **SSeI$_2$** followed by acid quenching.

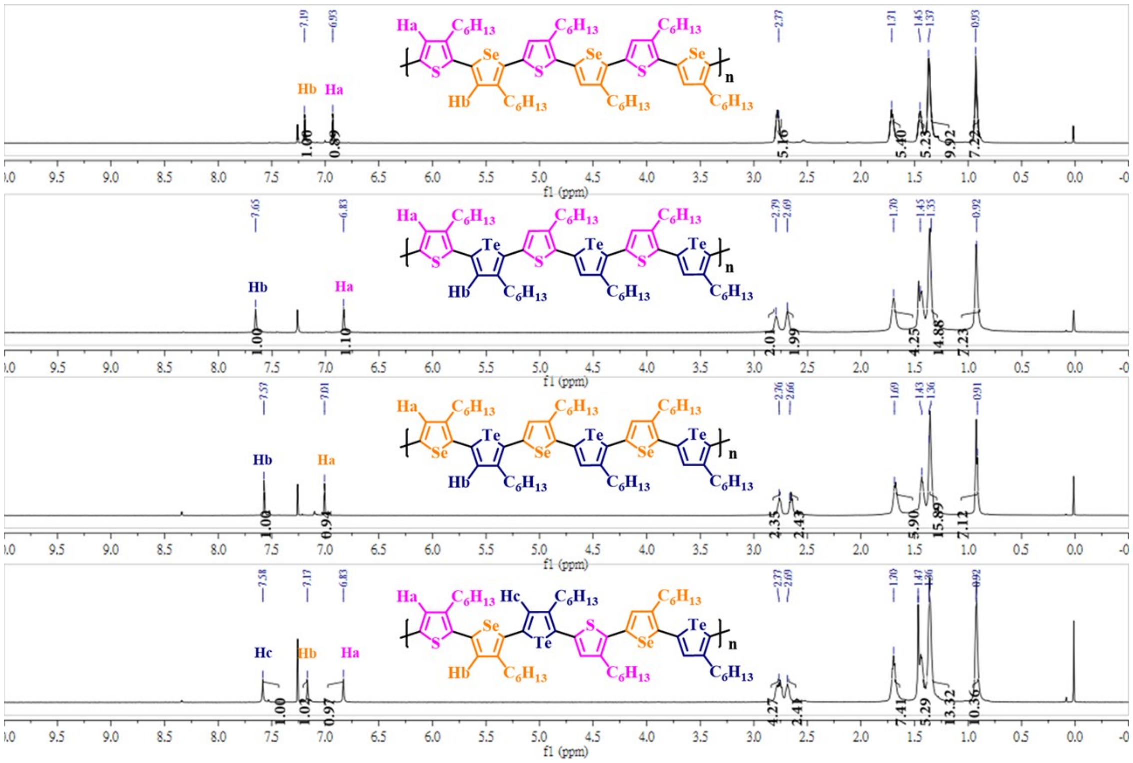

Figure S3. ^{1}H NMR spectra of **PSSe**, **PSTe**, **PSeTe**, and **PSSeTe**.

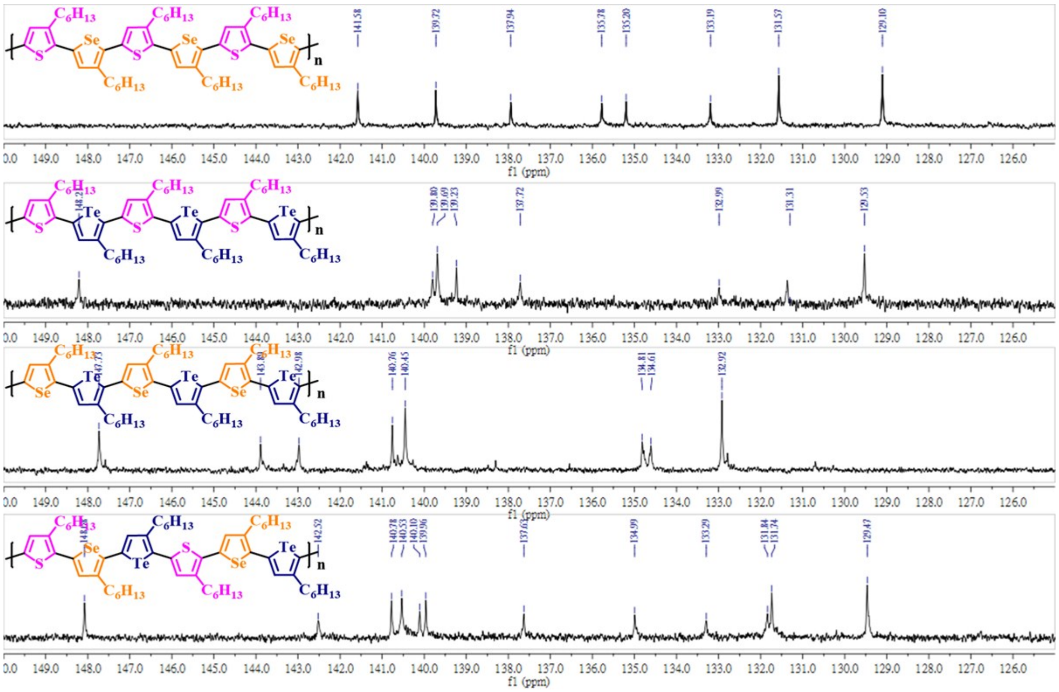

Figure S4. ^{13}C NMR spectra (aromatic region) of **PSSe**, **PSTe**, **PSeTe**, and **PSSeTe**.

Procedure for M_n versus Monomer Conversion Plot

To a solution of isopropylmagnesium chloride lithium chloride complex (1.3 M, 0.169 mL, 0.22 mmol) diluted by dried THF (6 mL) was added dropwisely a solution of **STeI$_2$** (150 mg, 0.22 mmol) with nonadecane (59 mg, 0.22 mmol) in dried THF (6 mL) as the internal standard. An aliquot was subjected to GC-MS analysis to determine the initial ratio of monomer to the internal standard (nonadecane). [1,3-bis(diphenylphosphino)propane]dichloronickel(II) (1.2 mg, 0.0022 mmol) was quickly added to the solution in one portion. Aliquots (1 mL) were taken periodically over a period of 5 min to determine the monomer conversion (GC-MS) and number average molecular weight (GPC).

Table S1. M_n and PDI versus monomer conversion of **PSTe**.

Monomer conversion (%)	M_n	PDI
8.3	552	1.20
70.8	10267	1.35
95.9	14931	1.19
97.3	15663	1.20
98.3	16162	1.19

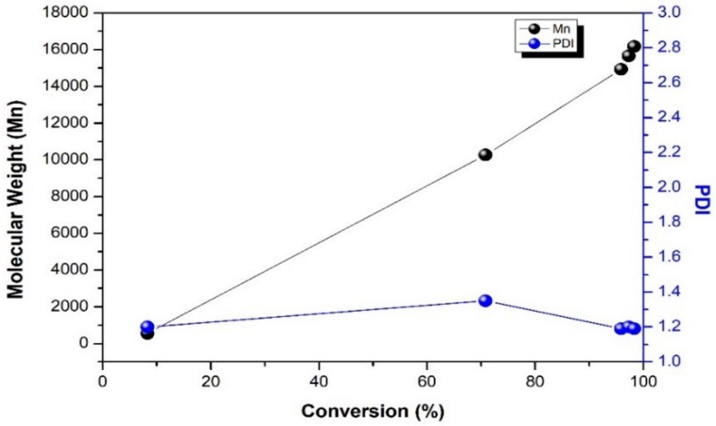

Figure S5. Dependence of M_n and PDI on monomer conversion for **PSTe**.

MALDI-TOF Mass Spectrometry of PSSeTe

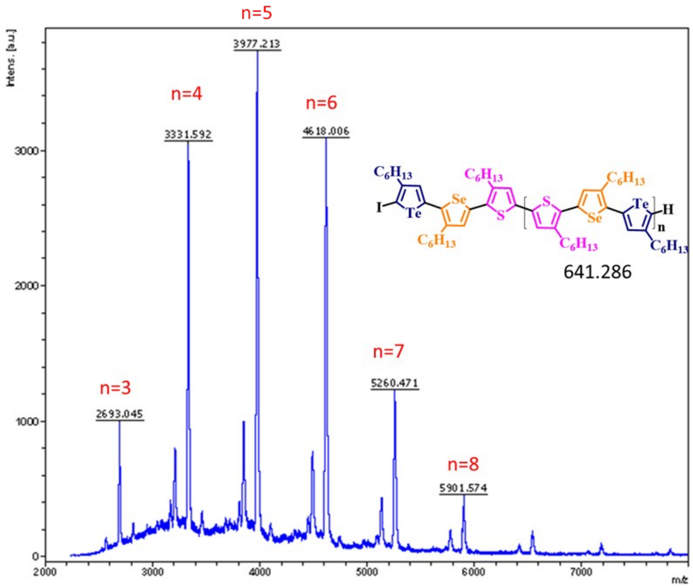

Figure S6. MALDI-TOF-MS of **PSSeTe**.

Characteristics of OFETs

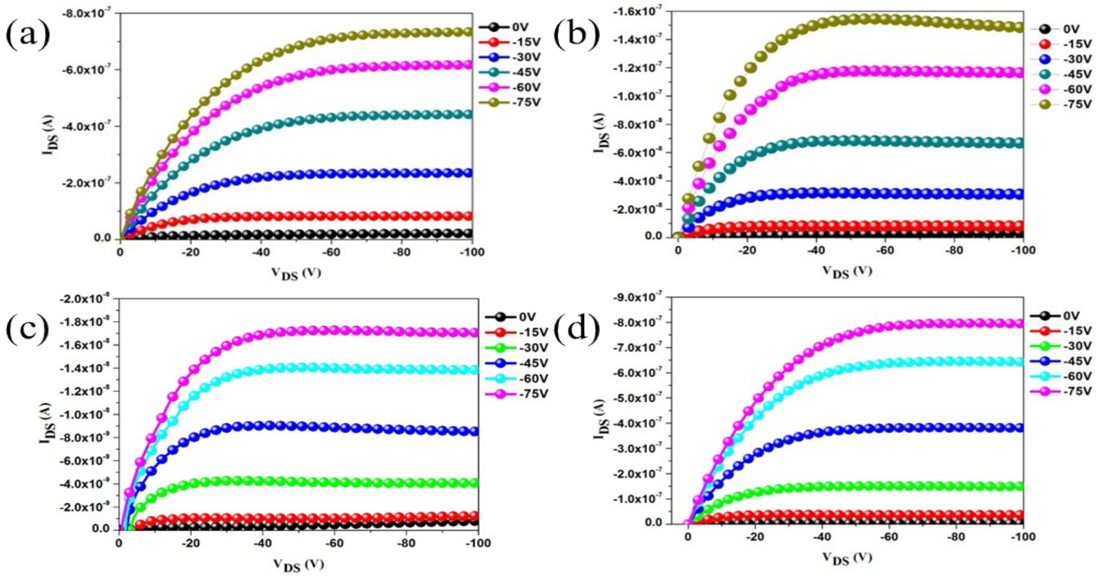

Figure S7. Output characteristics of OFETs based on (a) **PSSe**, (b) **PSTe**, (c) **PSeTe**, and (d) **PSSeTe**.

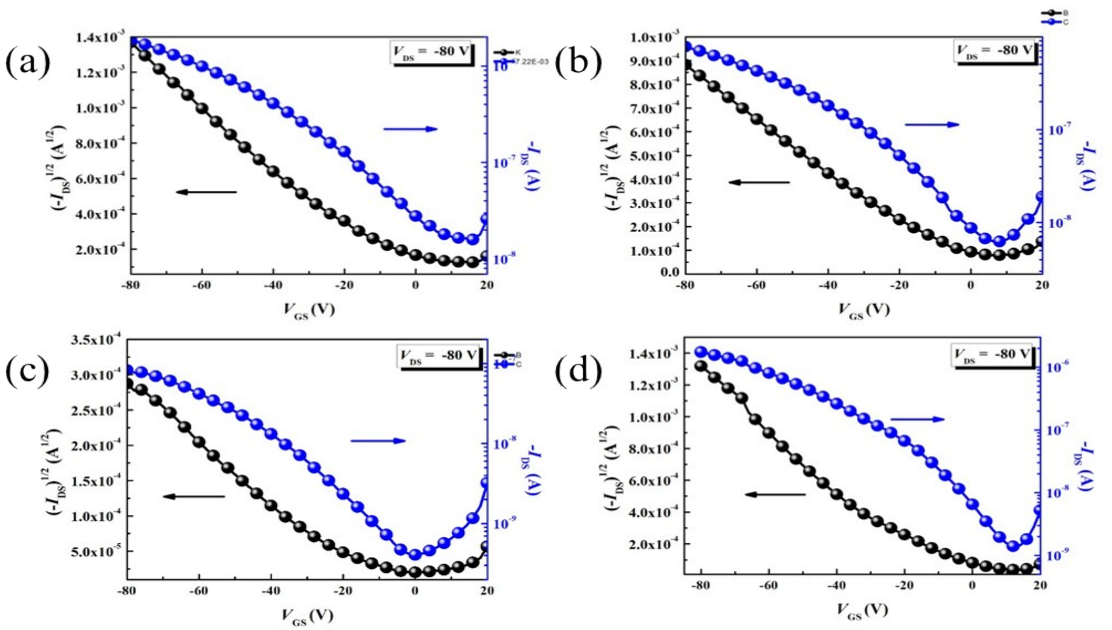

Figure S8. Transfer characteristics of OFETs based on (a) **PSSe**, (b) **PSTe**, (c) **PSeTe**, and (d) **PSSeTe**.

NMR Spectra

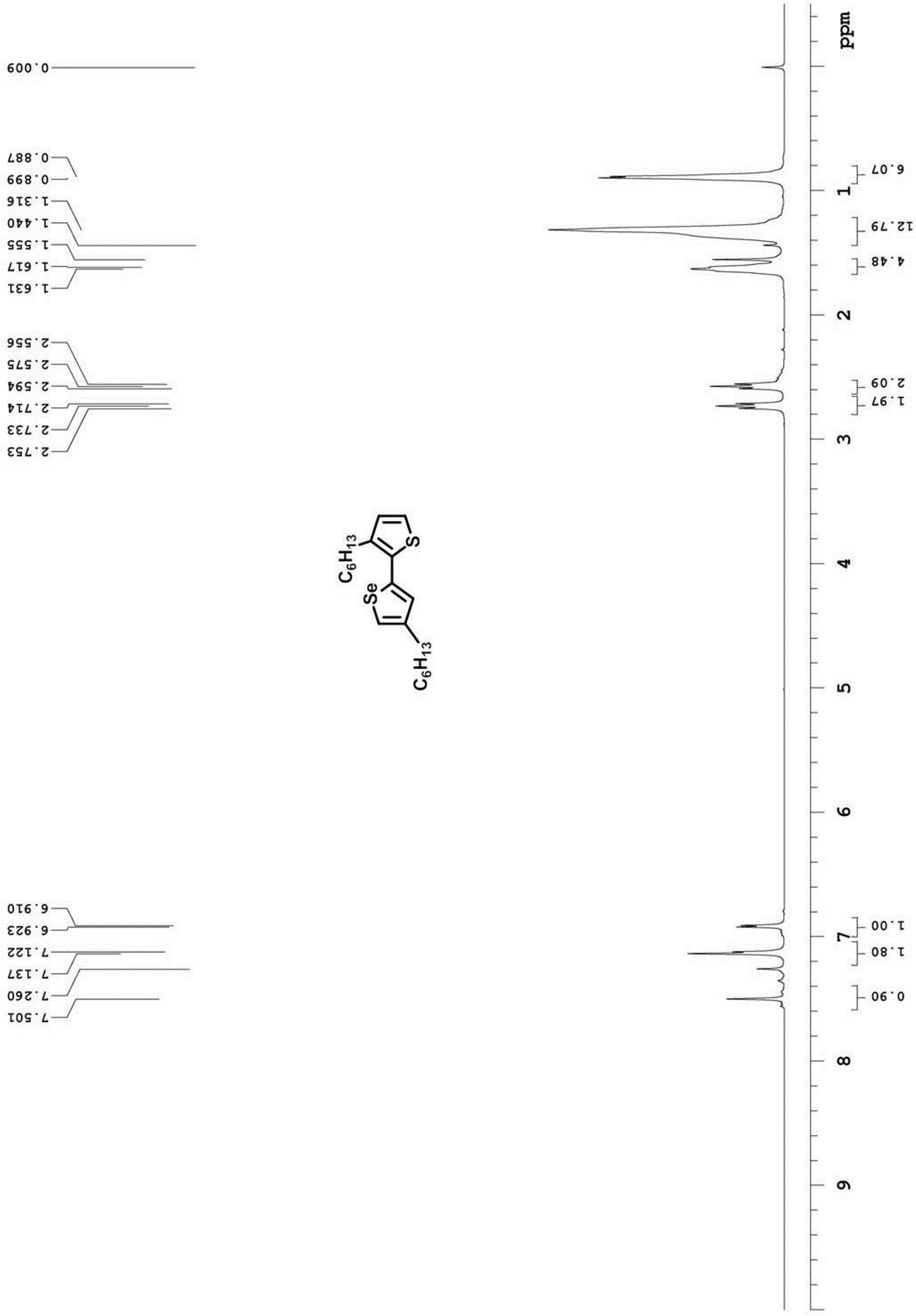

Figure S9. ^{1}H NMR spectrum of **SSe**.

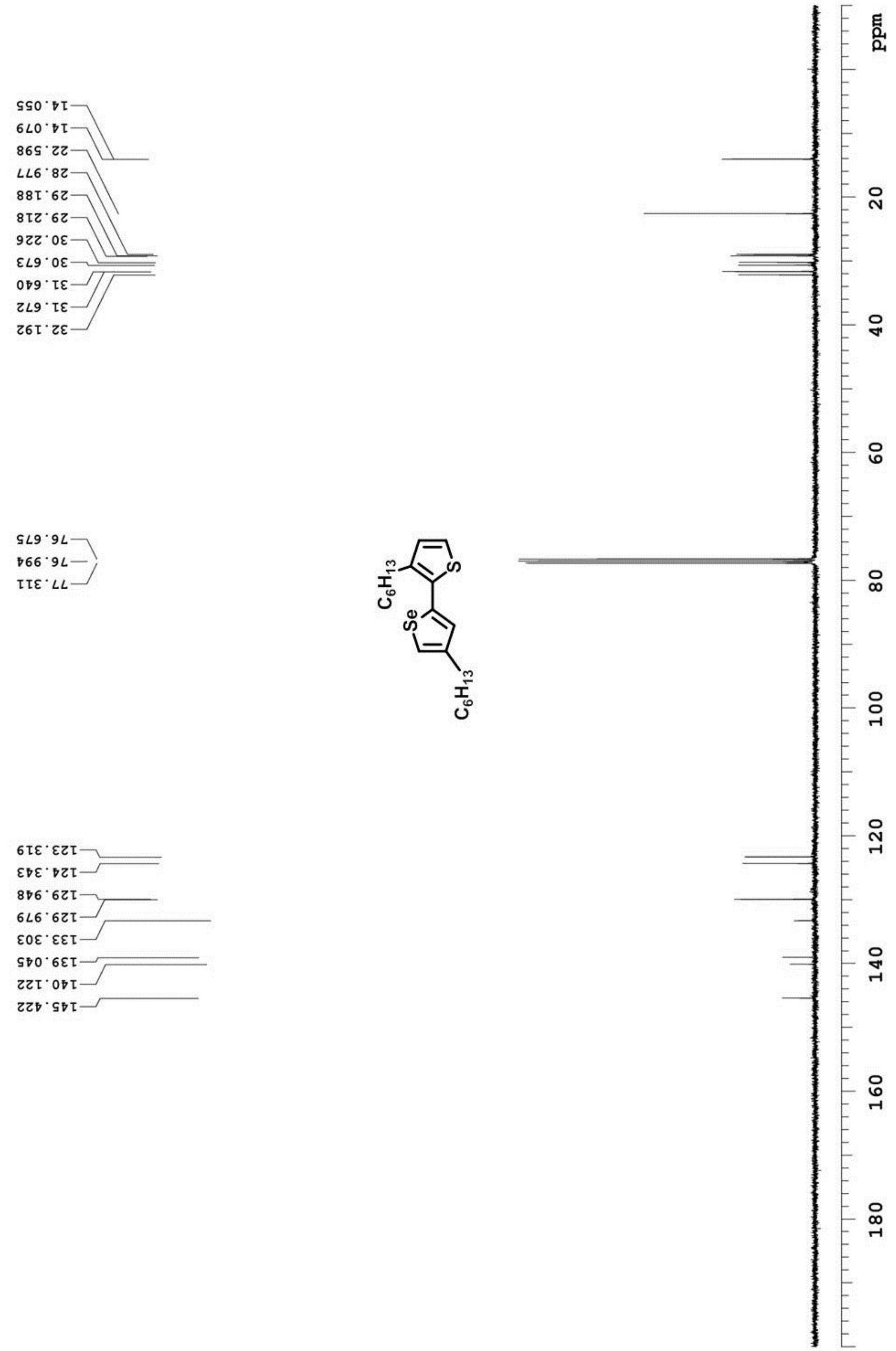

Figure S10. ^{13}C NMR spectrum of **SSe**.

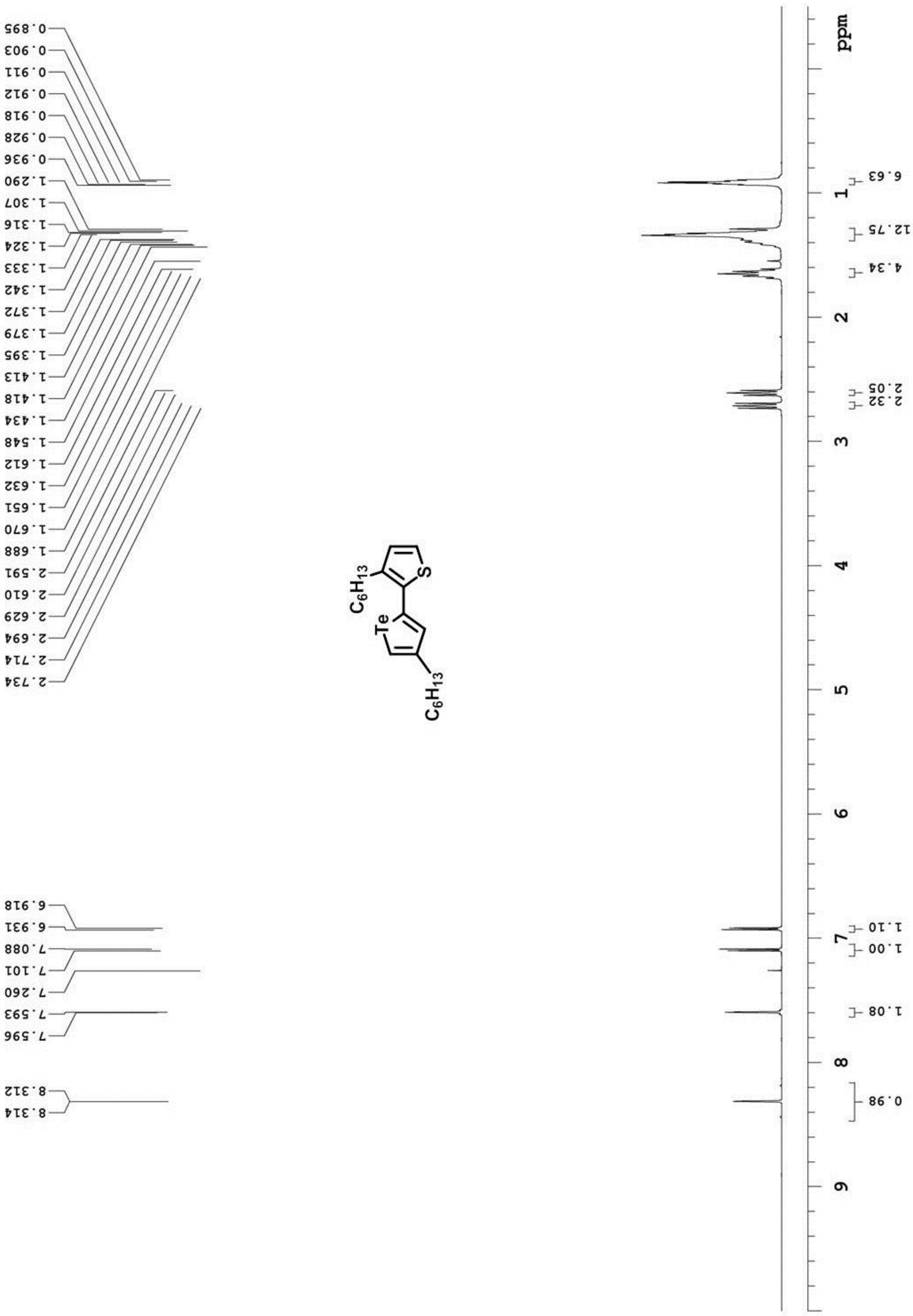

Figure S11. ^{1}H NMR spectrum of **STe**.

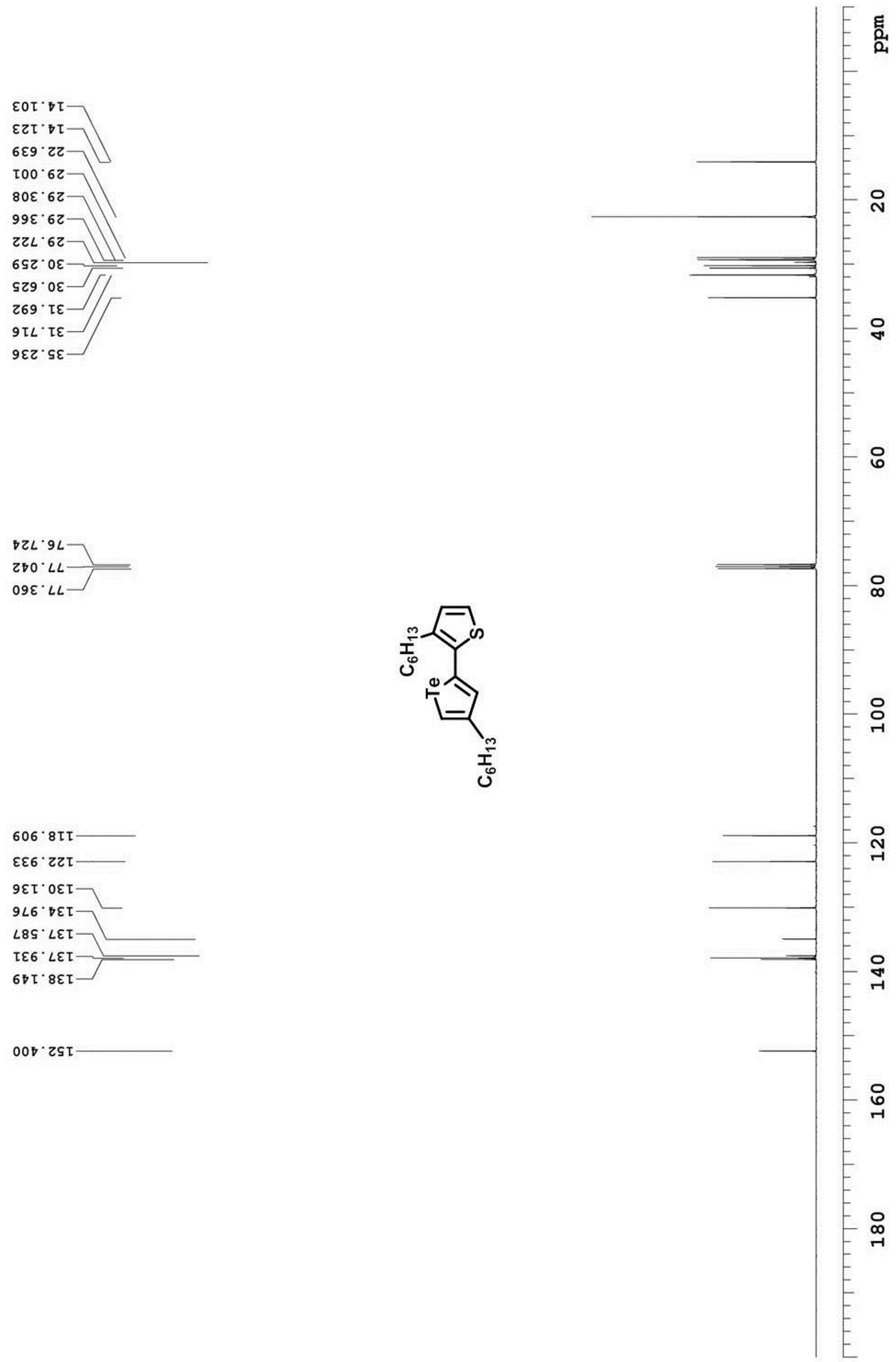

Figure S12. ^{13}C NMR spectrum of **STe**.

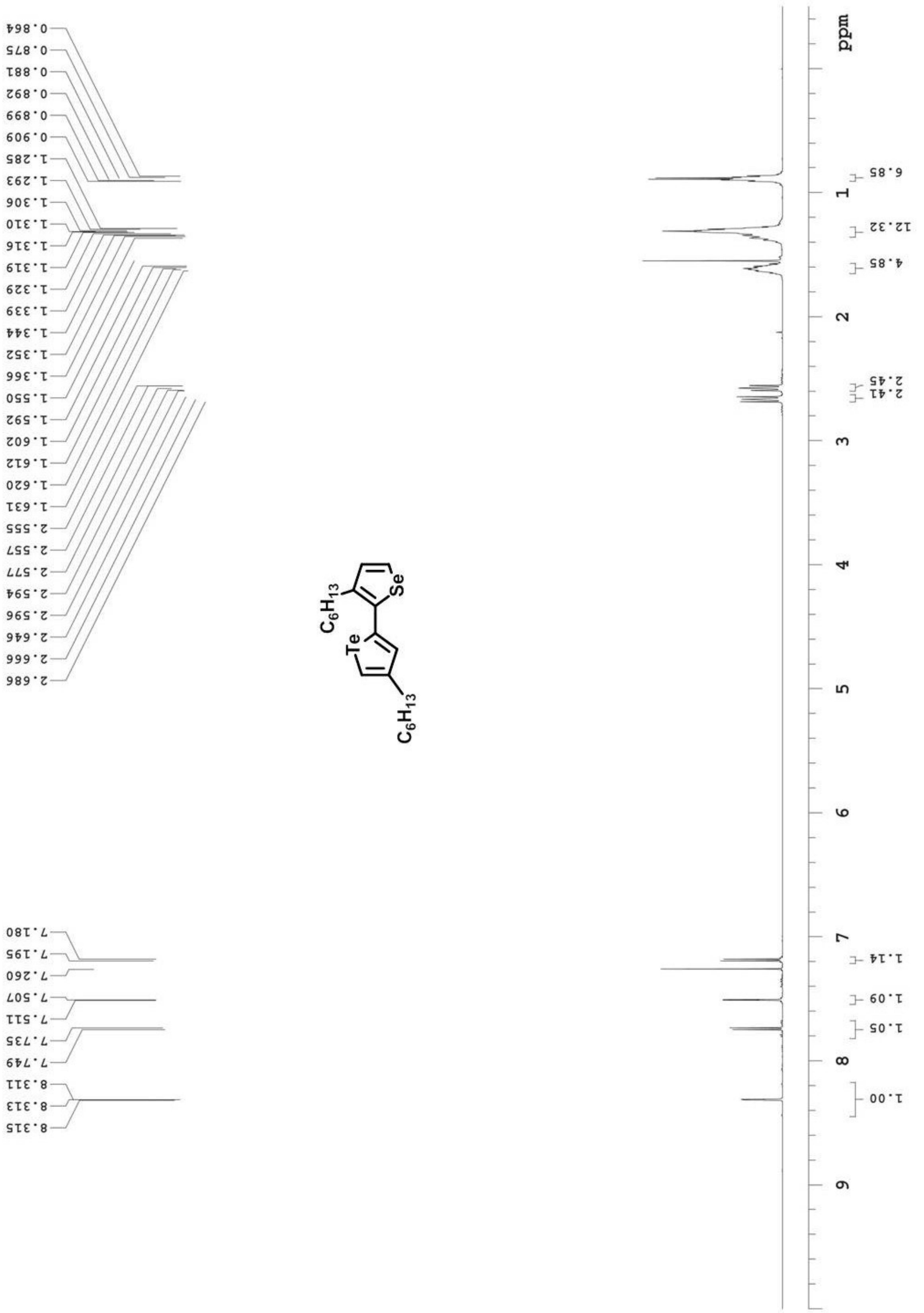

Figure S13. ^{1}H NMR spectrum of **SeTe**.

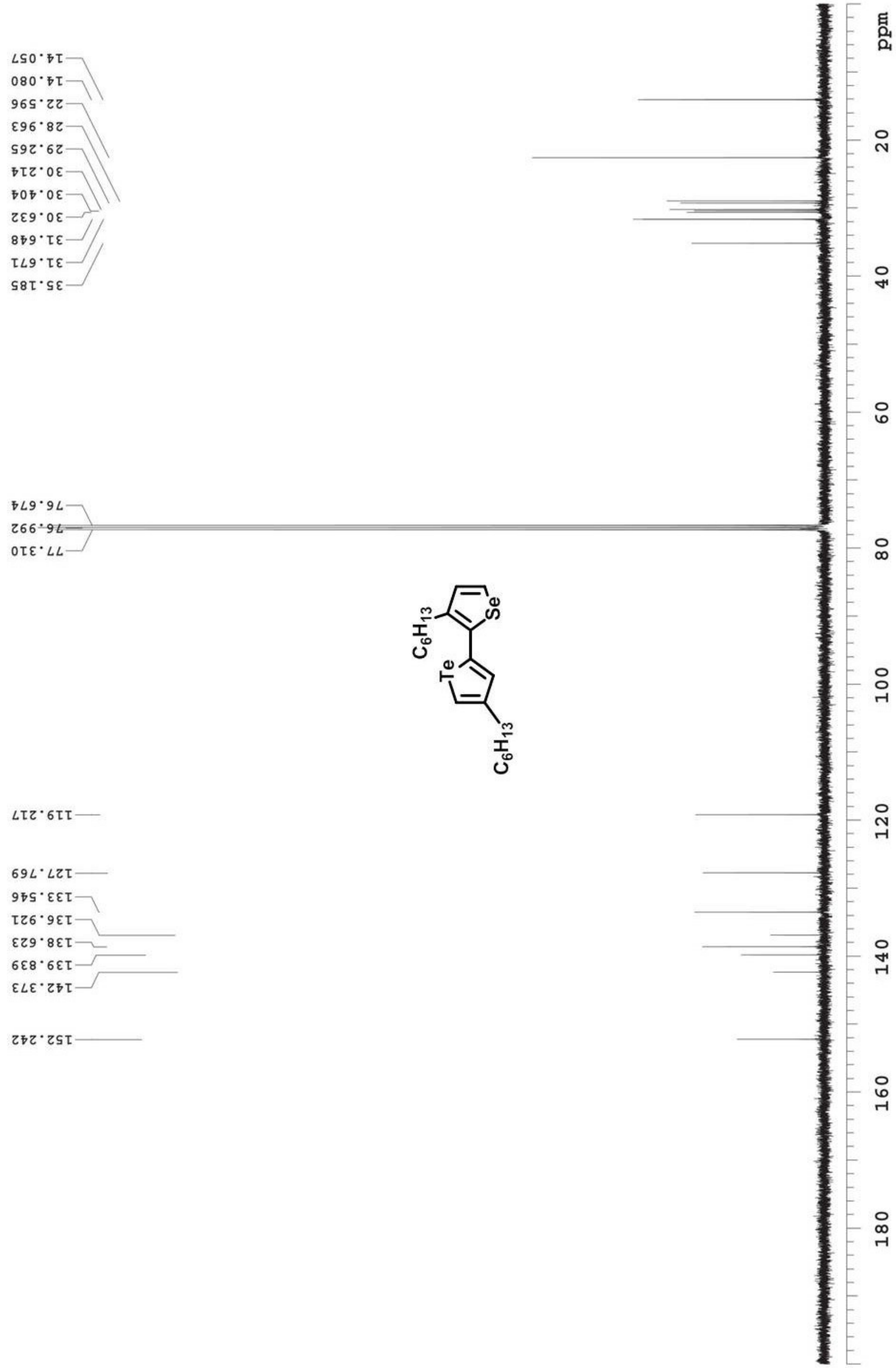

Figure S14. ^{13}C NMR spectrum of **SeTe**.

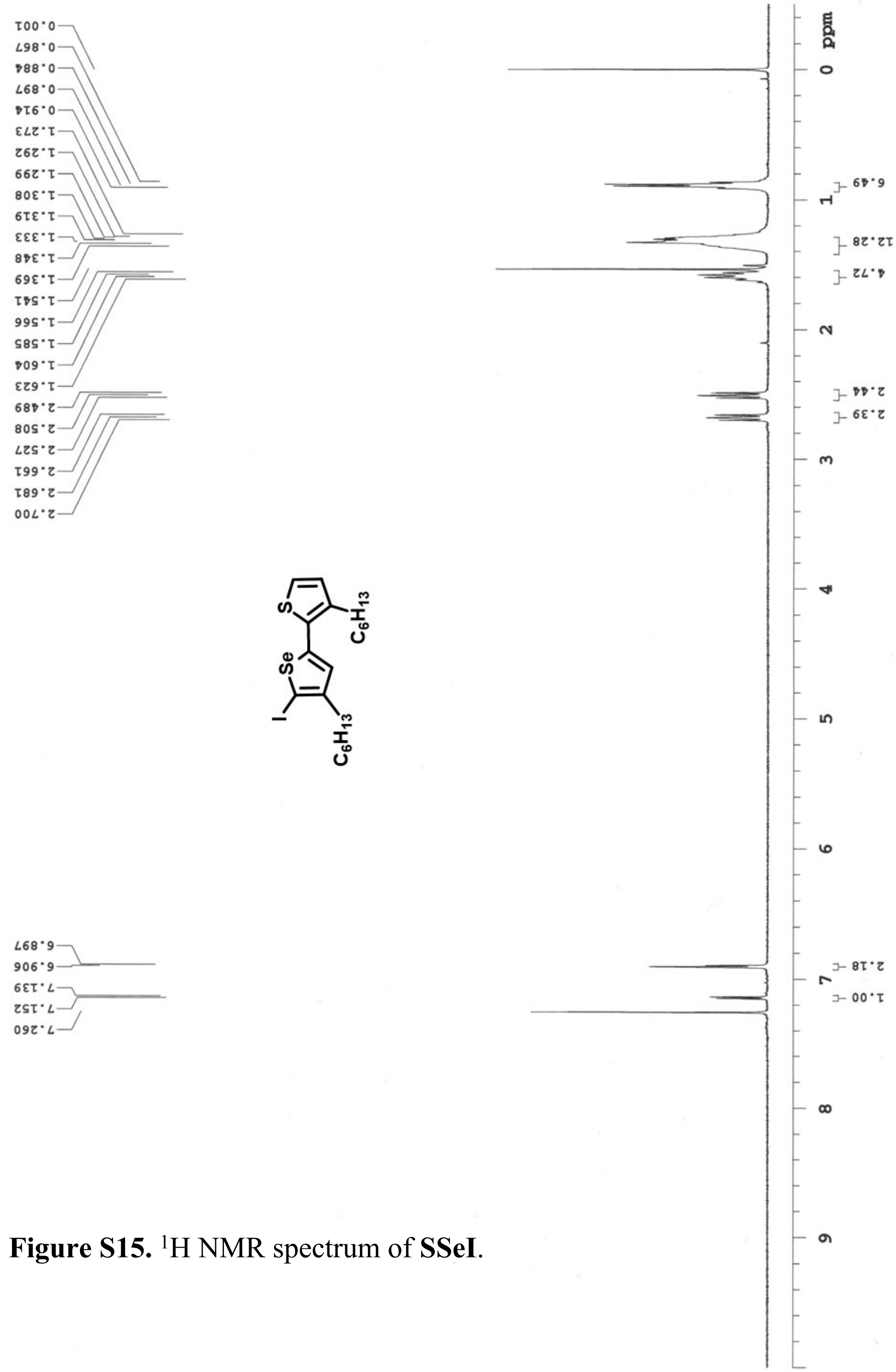

Figure S15. ^{1}H NMR spectrum of **SSeI**.

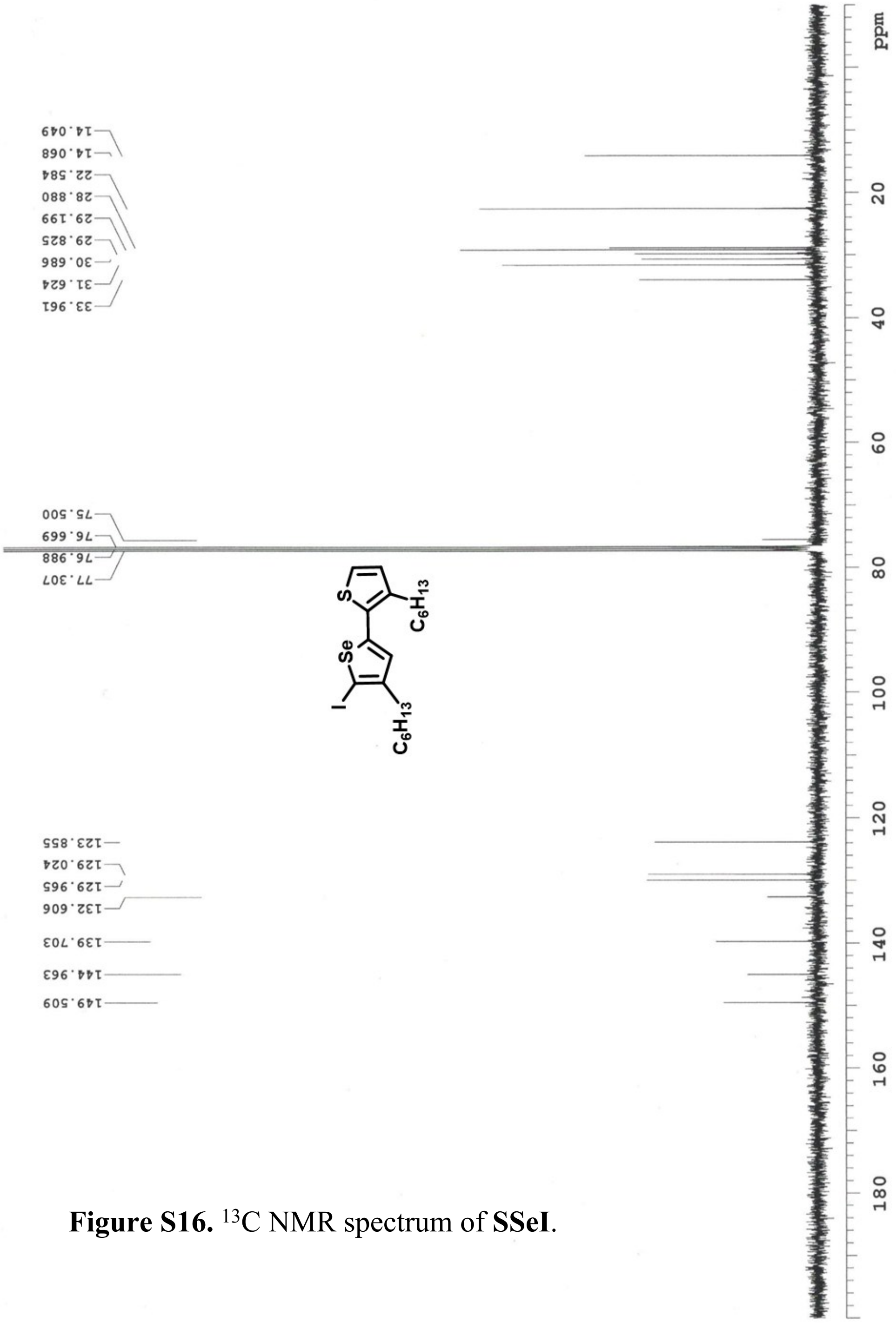

Figure S16. ^{13}C NMR spectrum of **SSeI**.

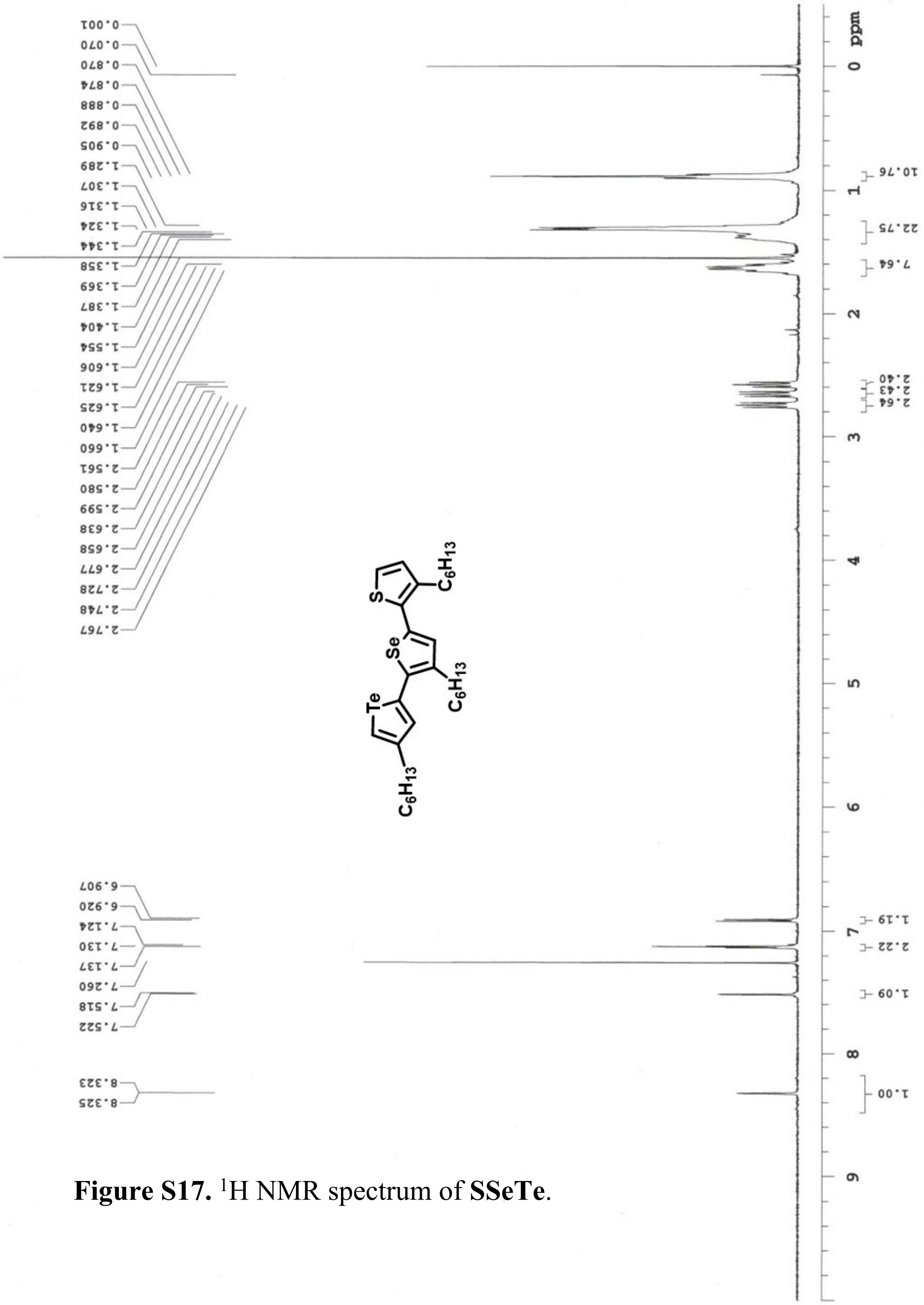

Figure S17. ^{1}H NMR spectrum of **SSeTe**.

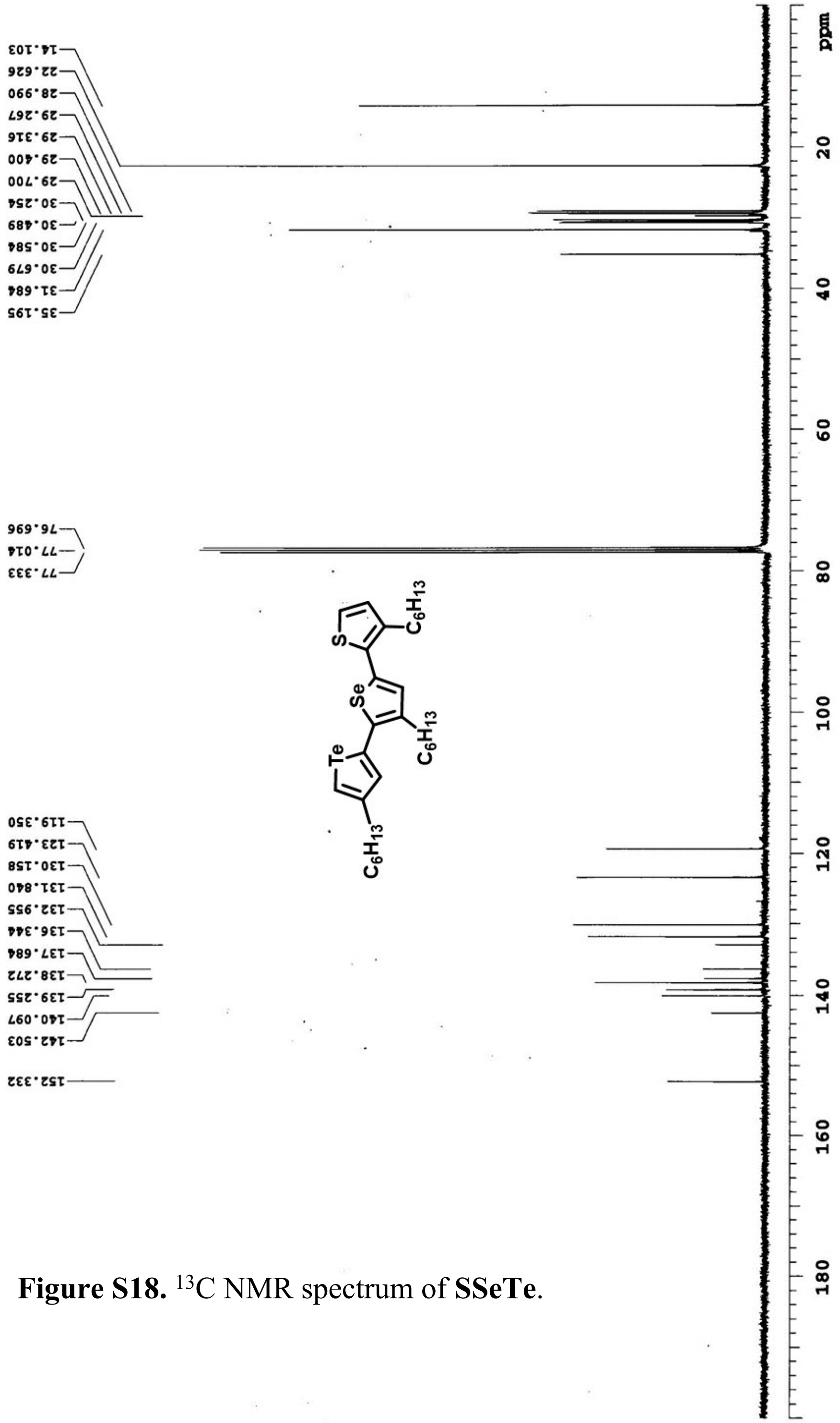

Figure S18. ^{13}C NMR spectrum of **SSeTe**.

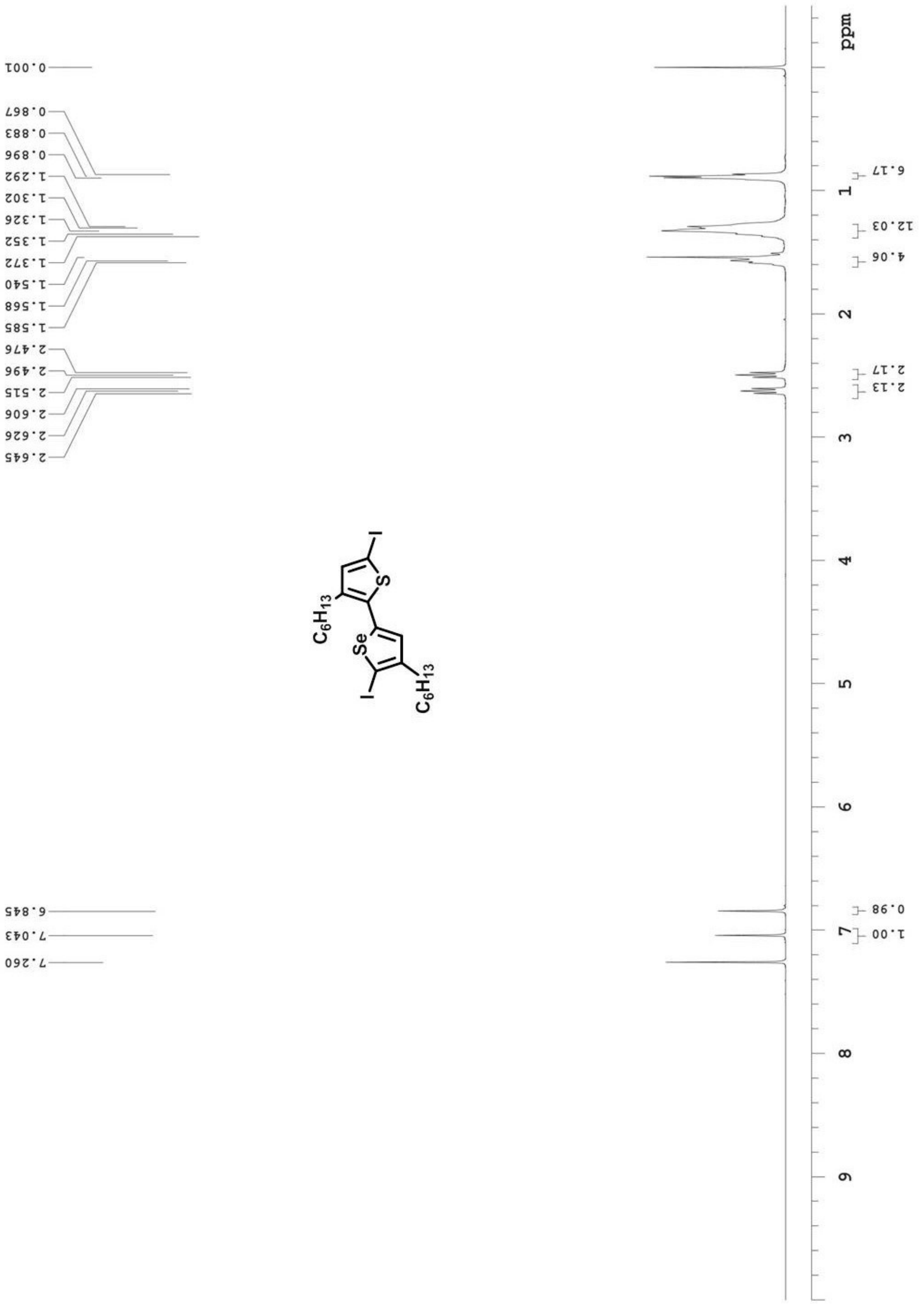

Figure S19. ^{1}H NMR spectrum of **$SSeI_2$**.

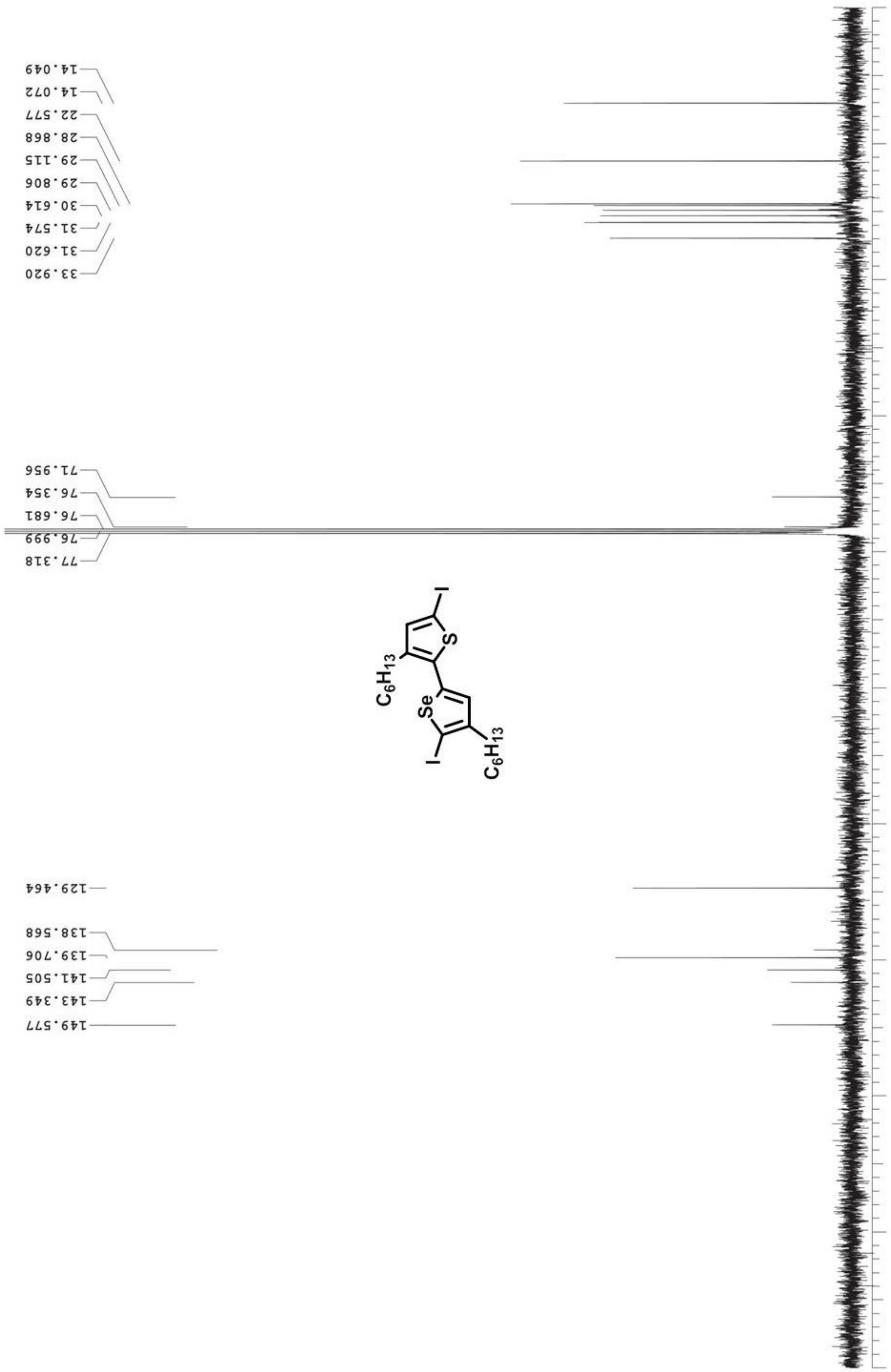

Figure S20. ^{13}C NMR spectrum of **$SSeI_2$**.

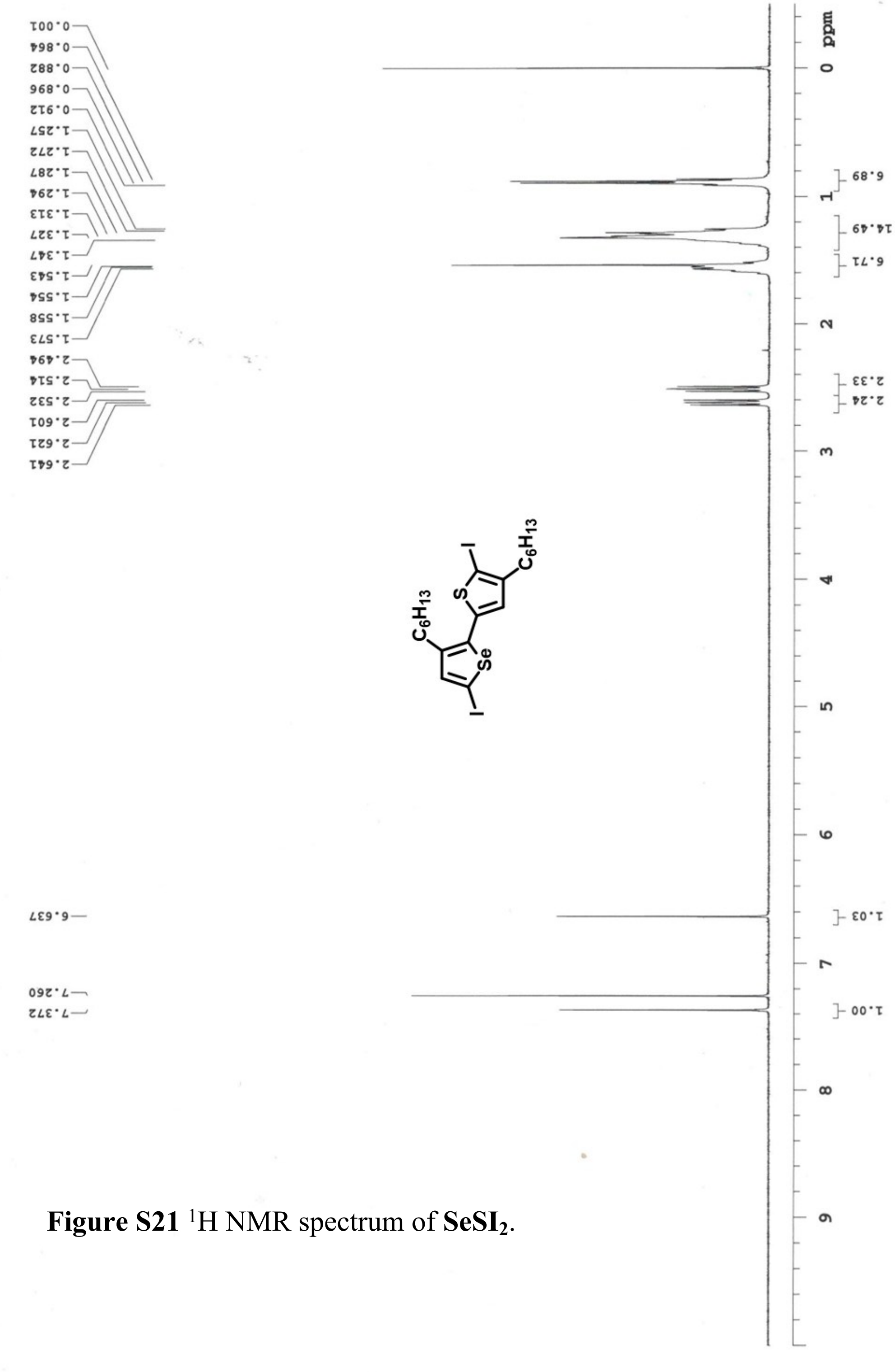

Figure S21 ^{1}H NMR spectrum of **SeSI$_2$**.

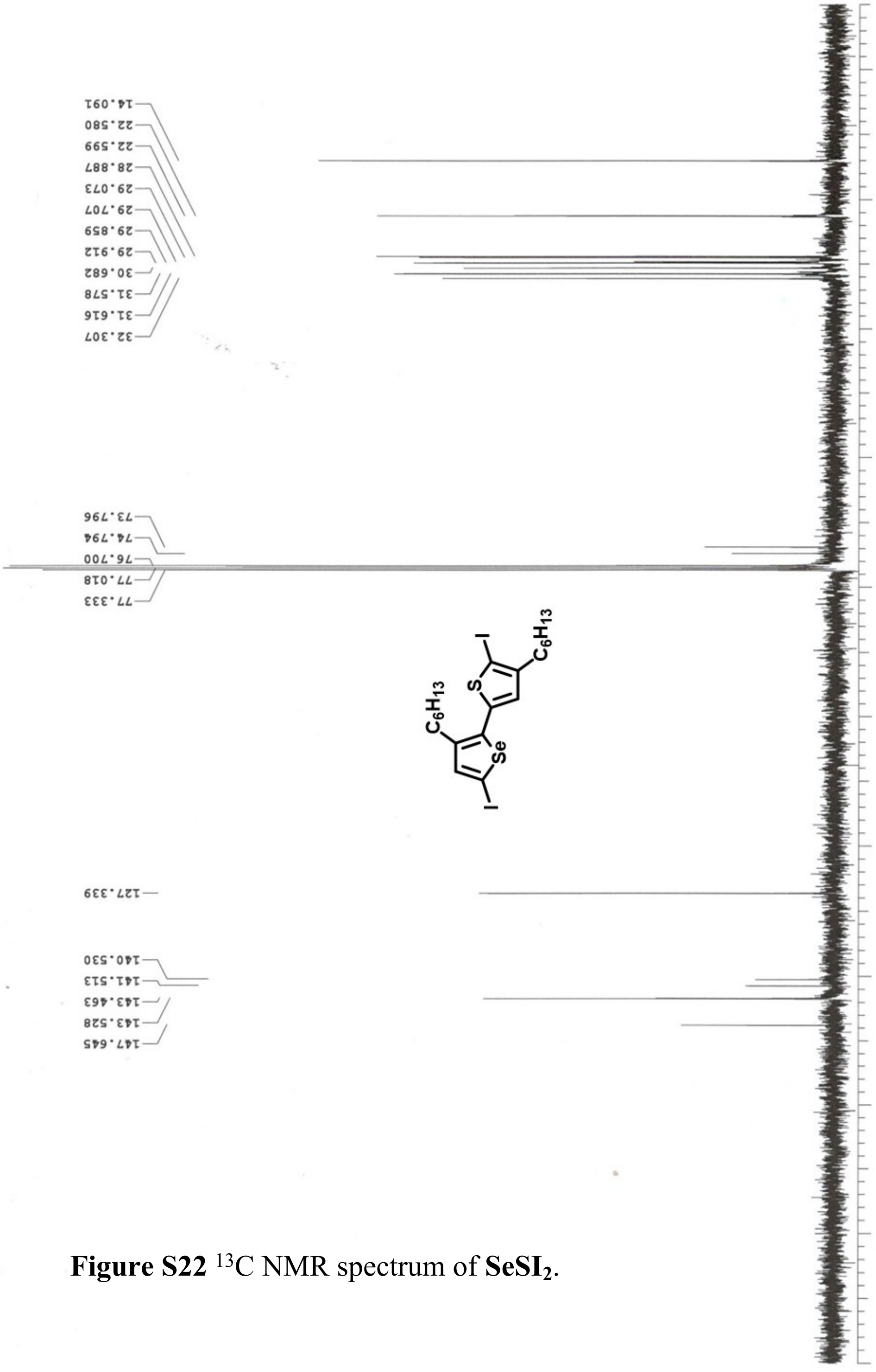

Figure S22 ^{13}C NMR spectrum of **SeSI$_2$**.

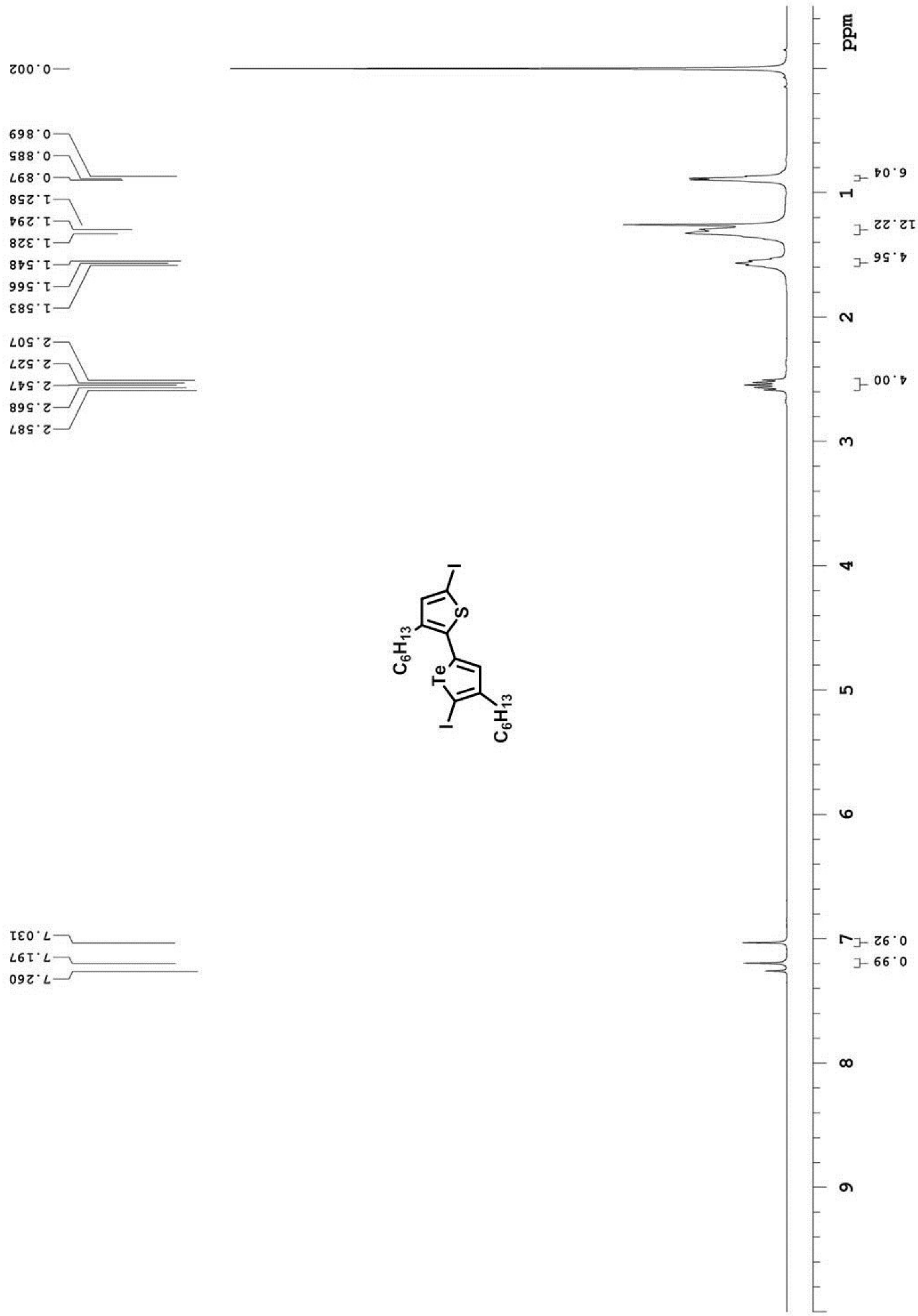

Figure S23. ^{1}H NMR spectrum of **STeI$_2$**.

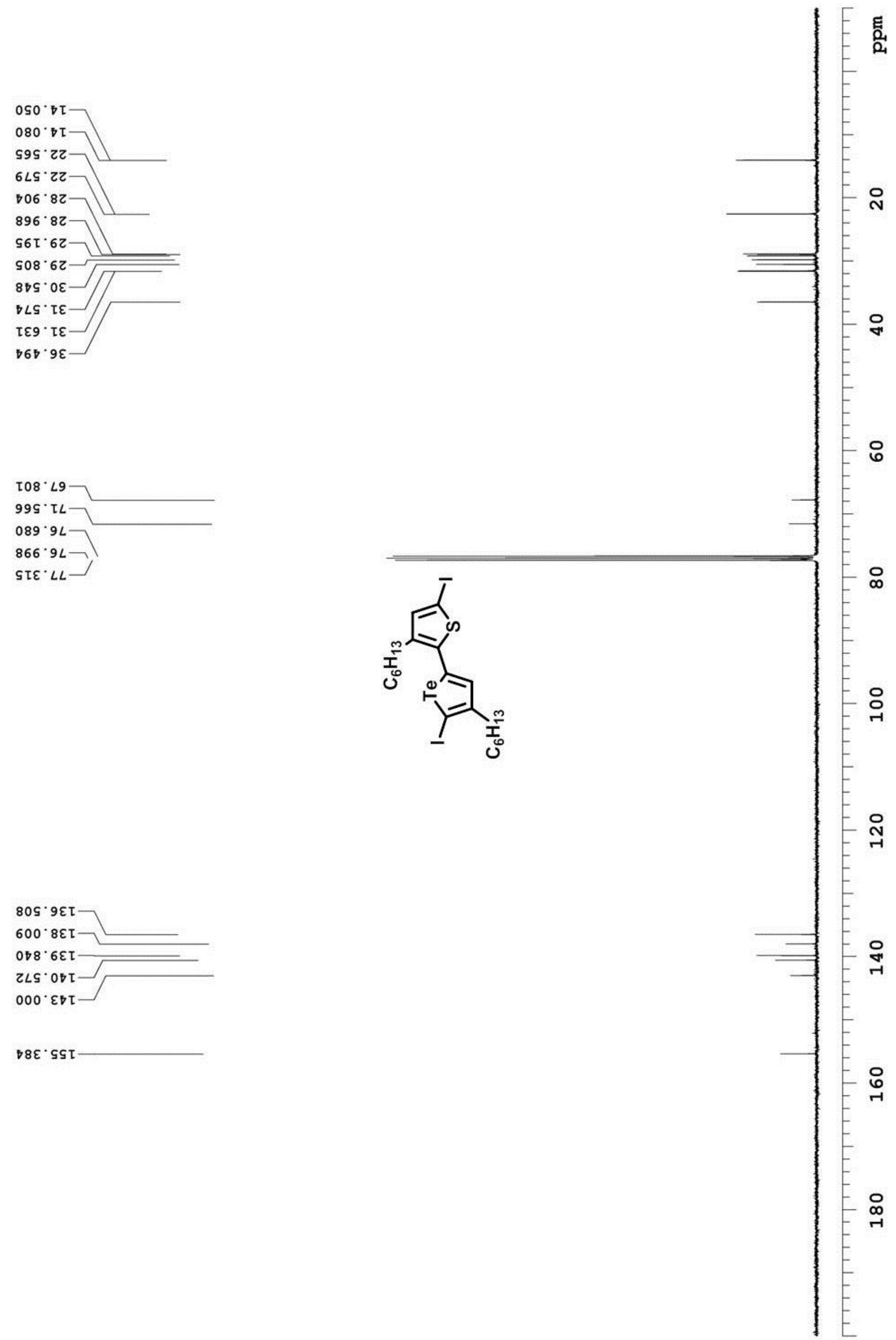

Figure S24. ^{13}C NMR spectrum of **$STeI_2$**.

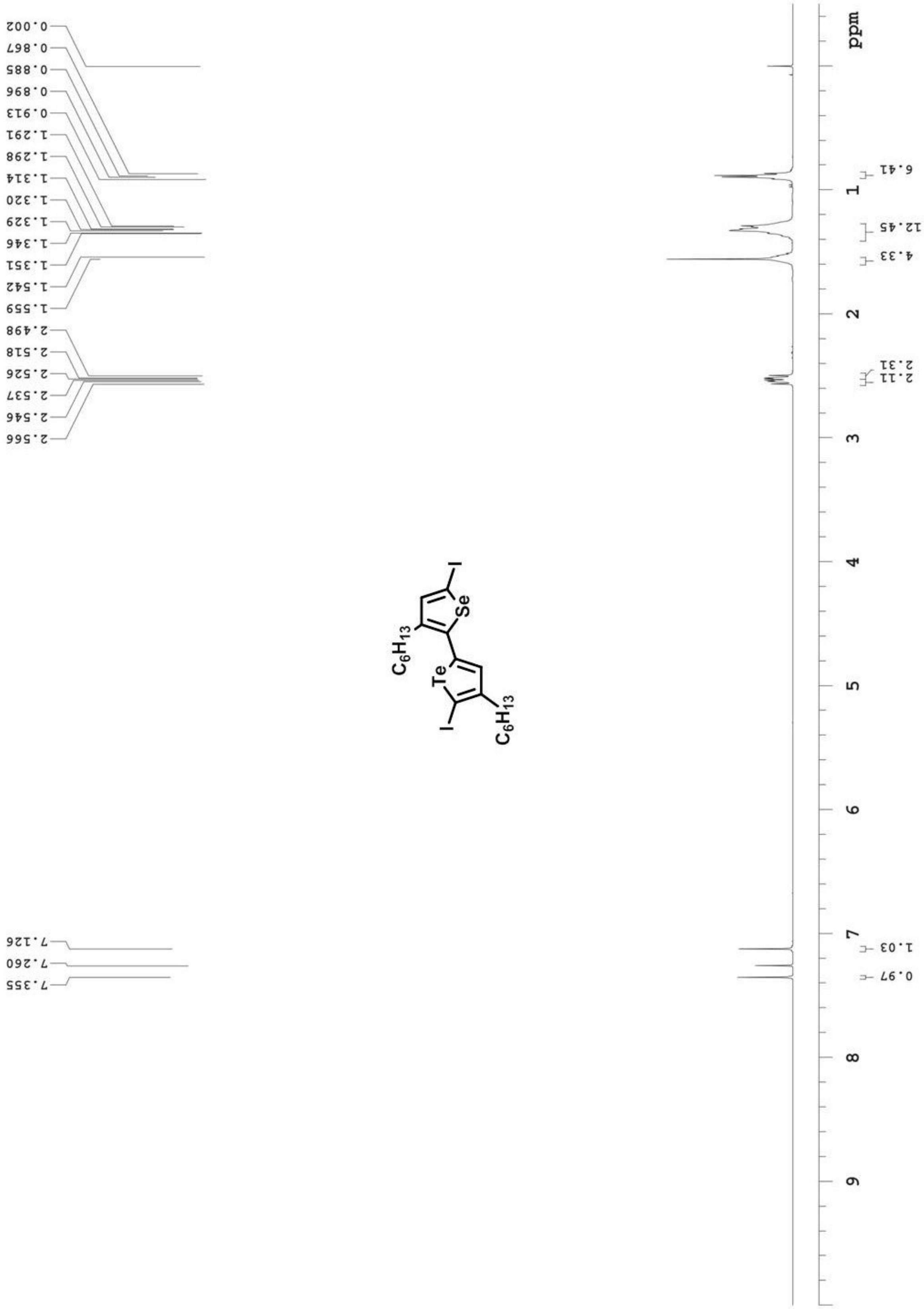

Figure S25. ^{1}H NMR spectrum of **SeTeI$_2$**.

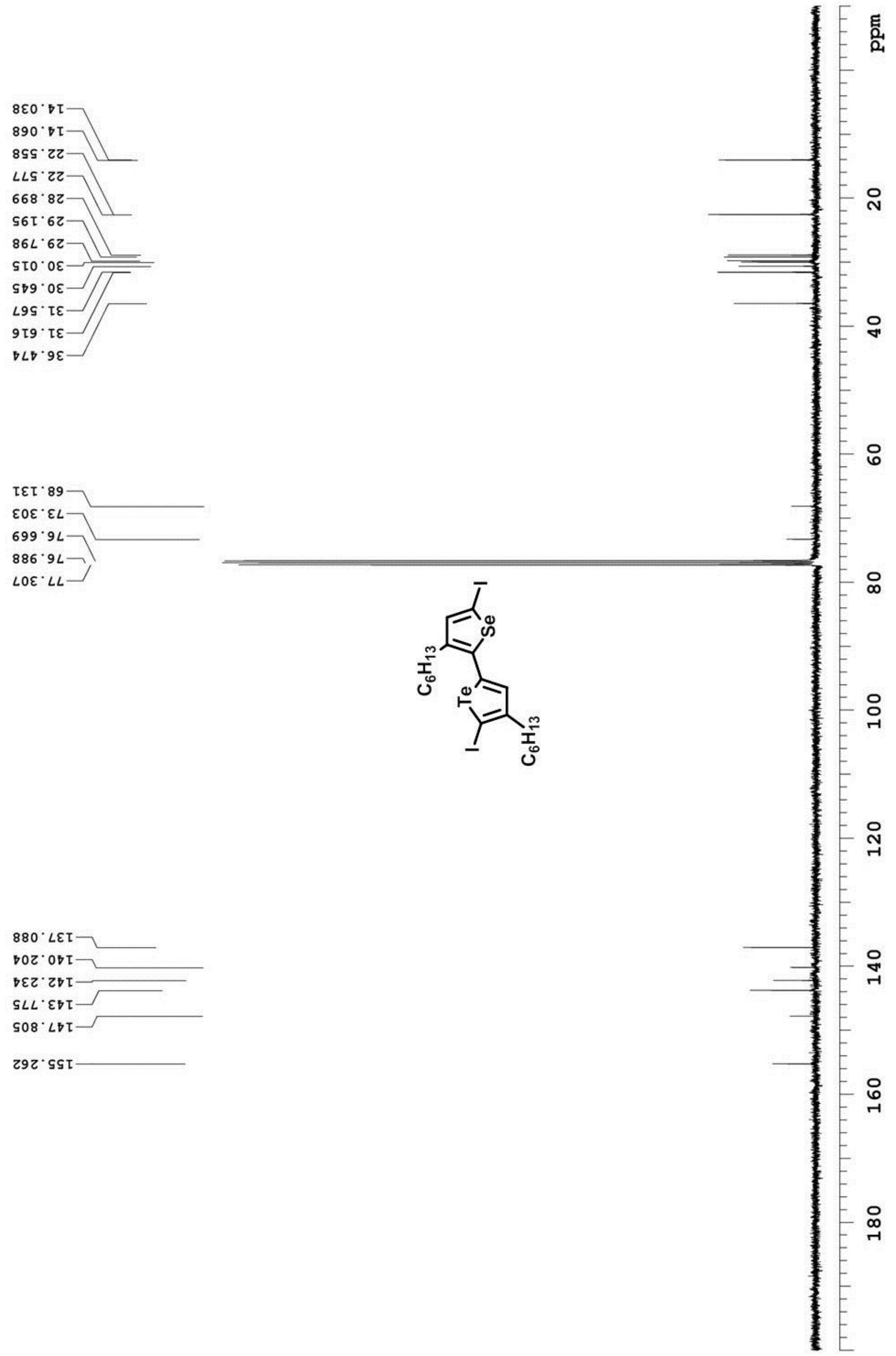

Figure S26. ^{13}C NMR spectrum of **$SeTeI_2$**.

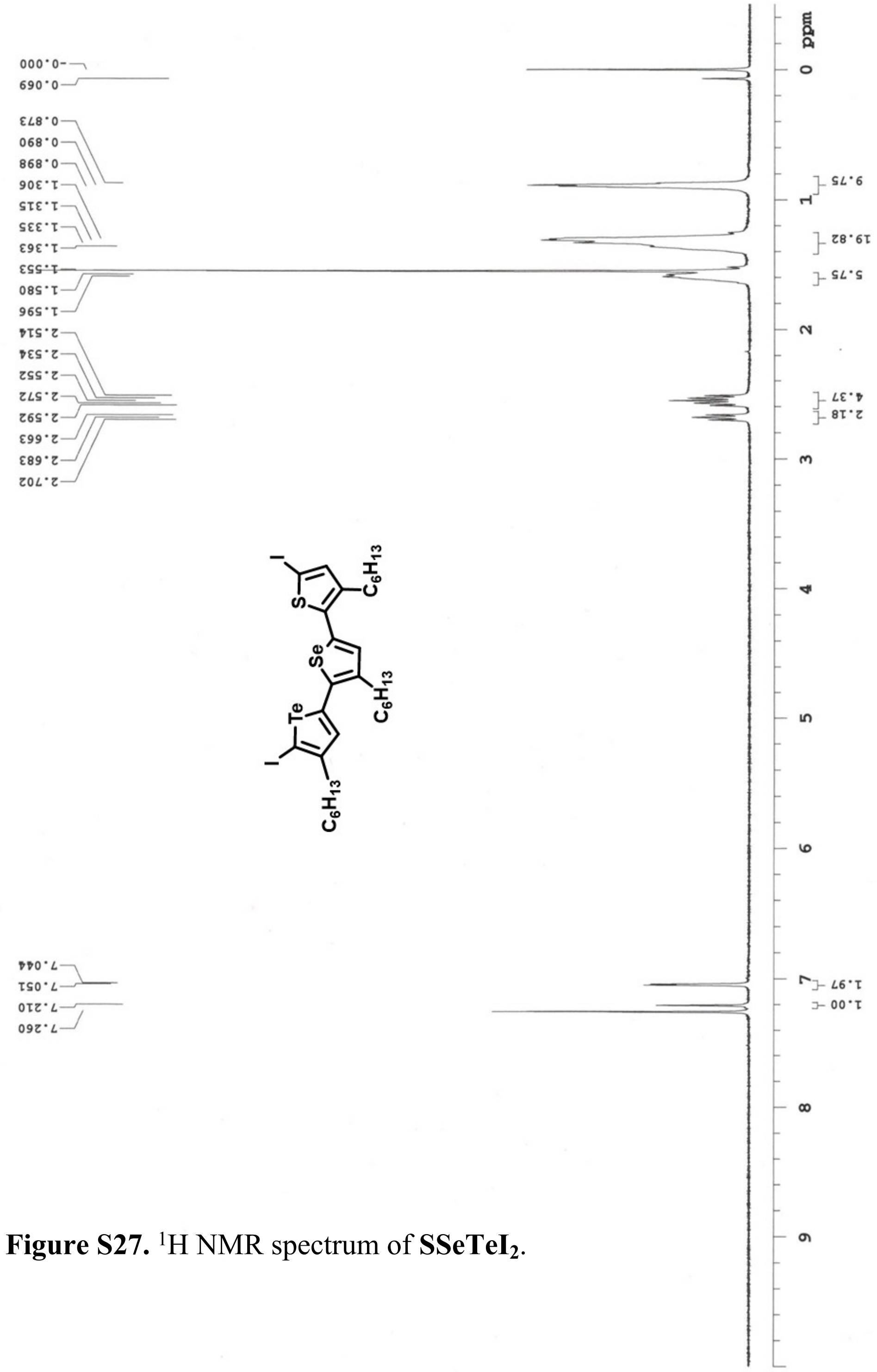

Figure S27. ^{1}H NMR spectrum of **SSeTeI$_2$**.

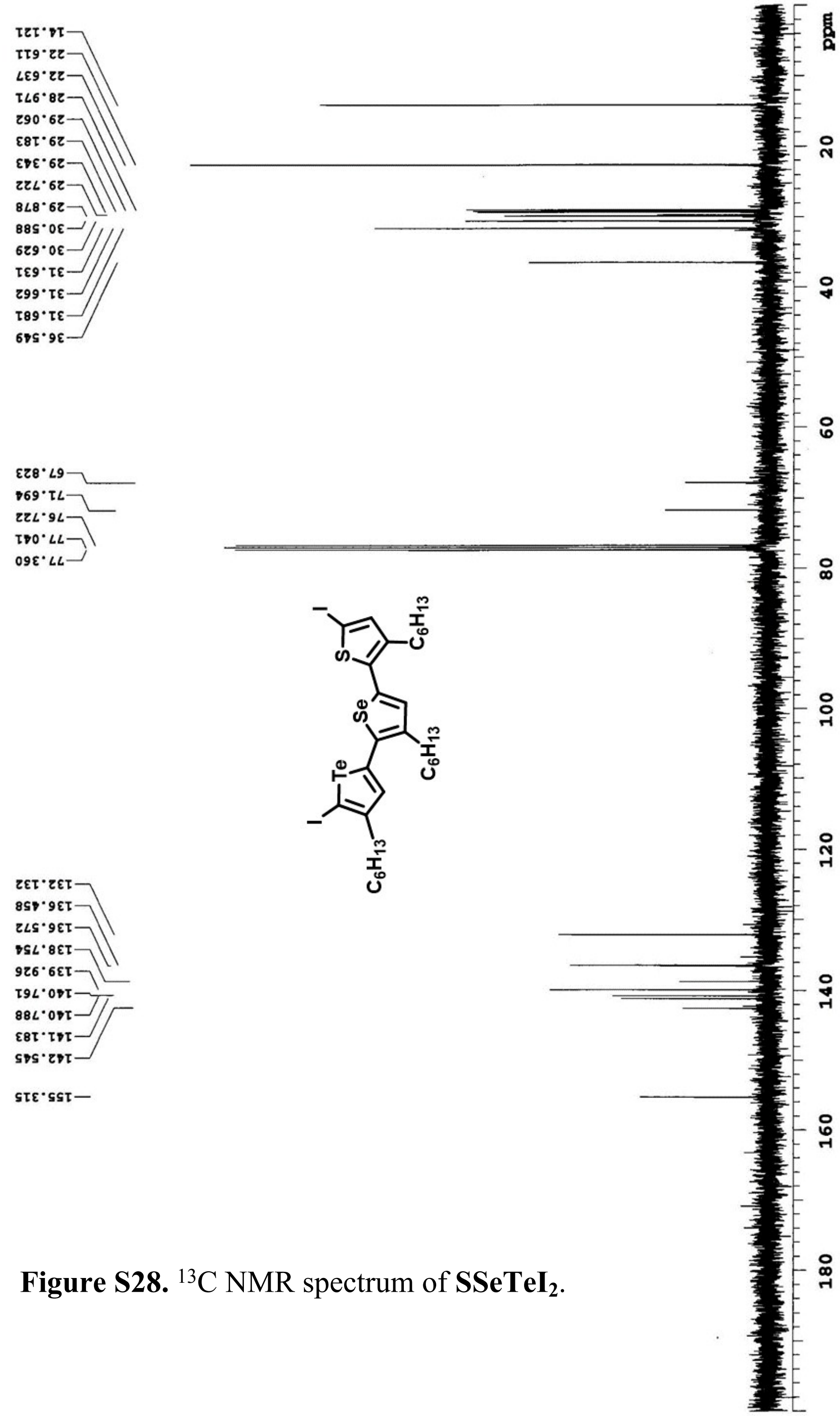

Figure S28. ^{13}C NMR spectrum of **$SSeTeI_2$**.

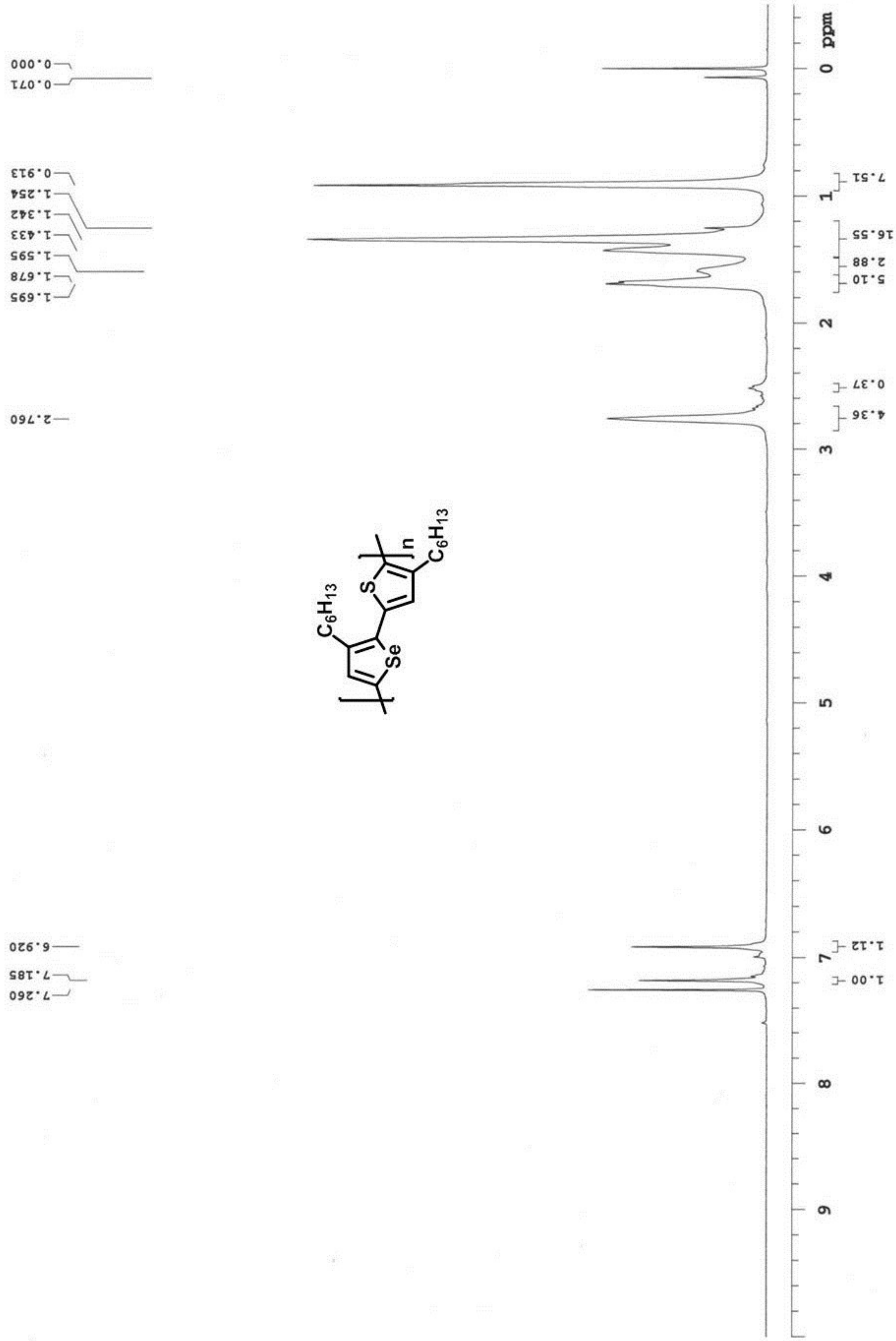

Figure S29. ^{1}H NMR spectrum of **PSSe**.

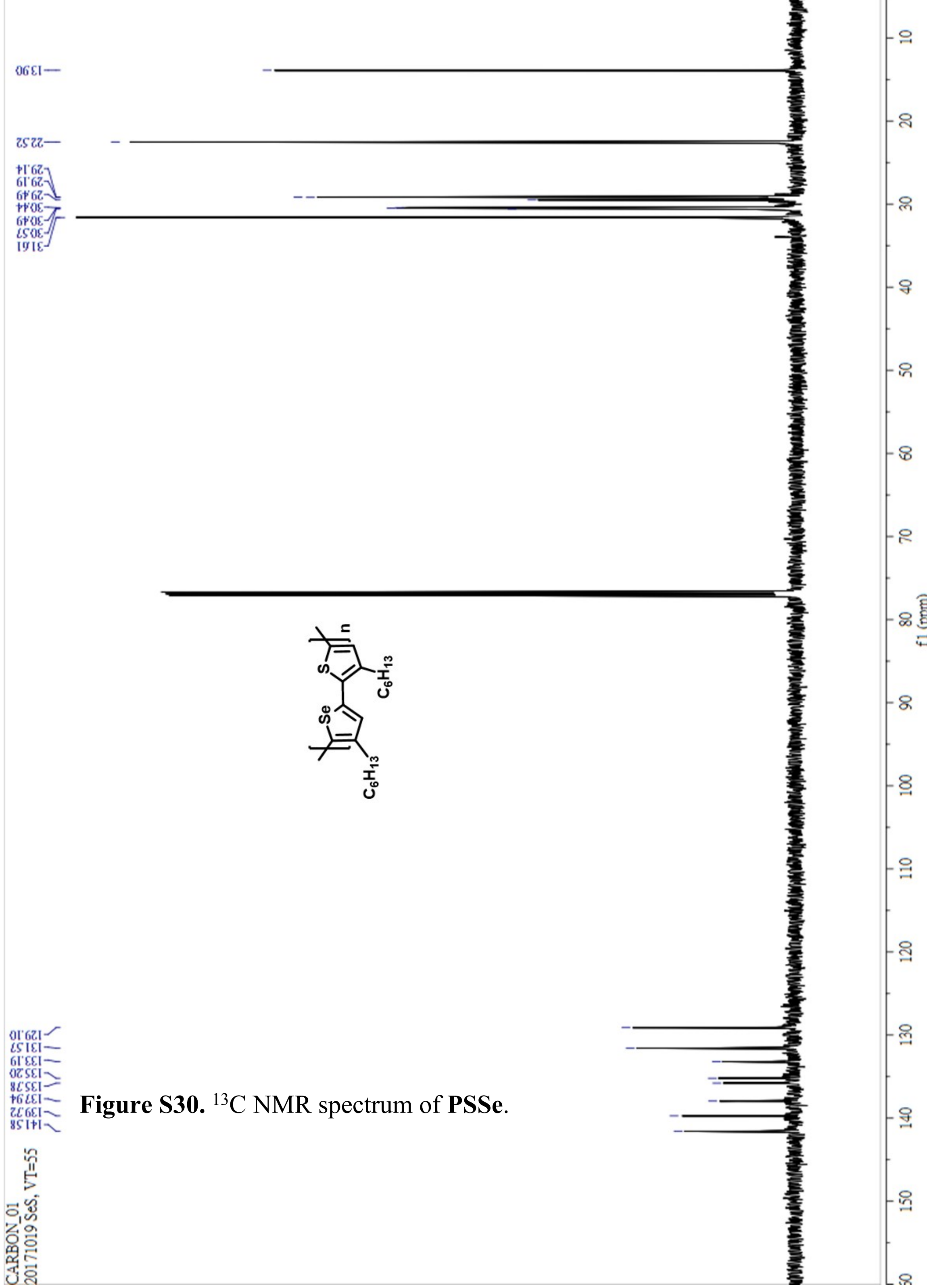

Figure S30. ^{13}C NMR spectrum of **PSSe**.

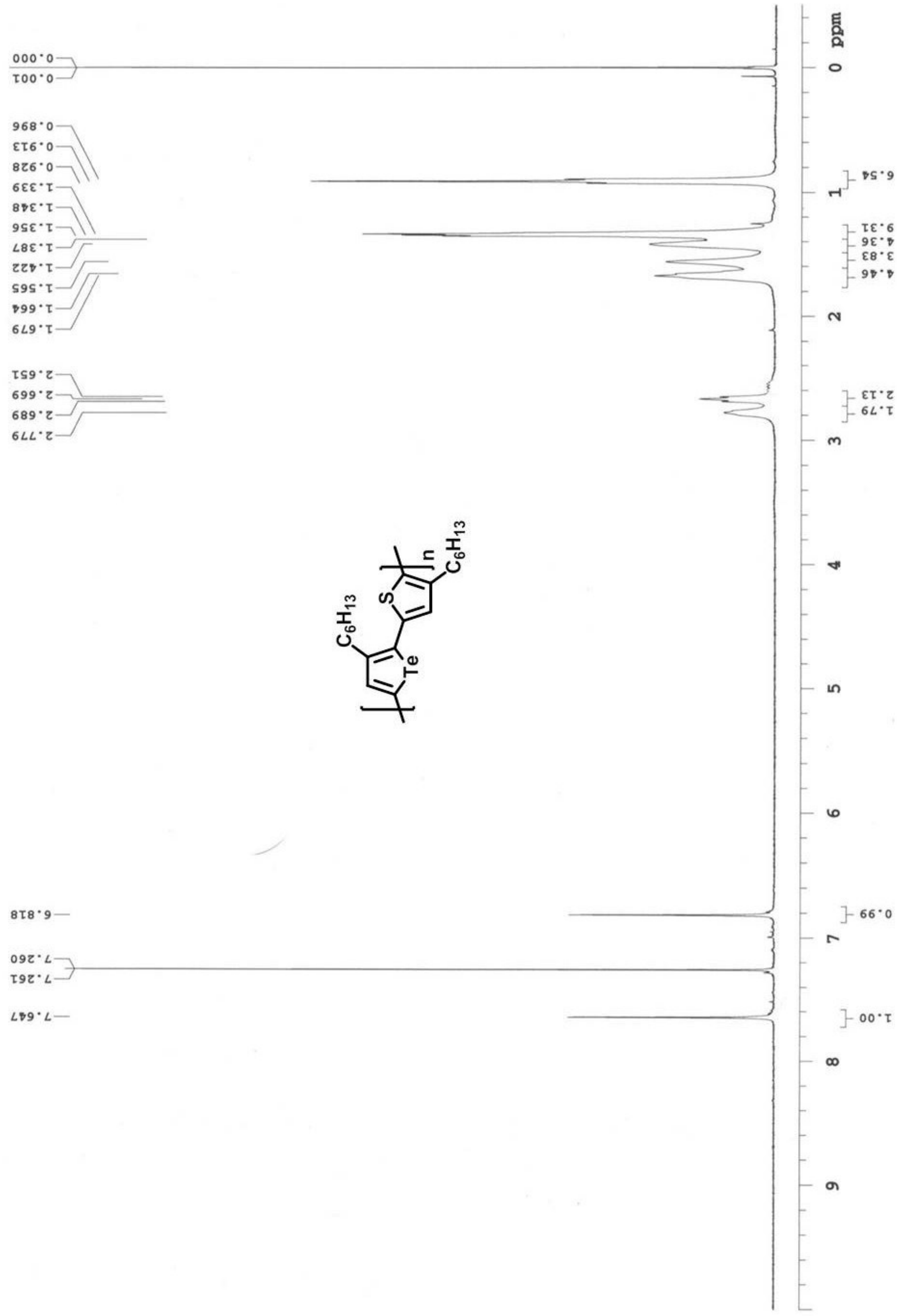

Figure S31. ^{1}H NMR spectrum of **PSTe**.

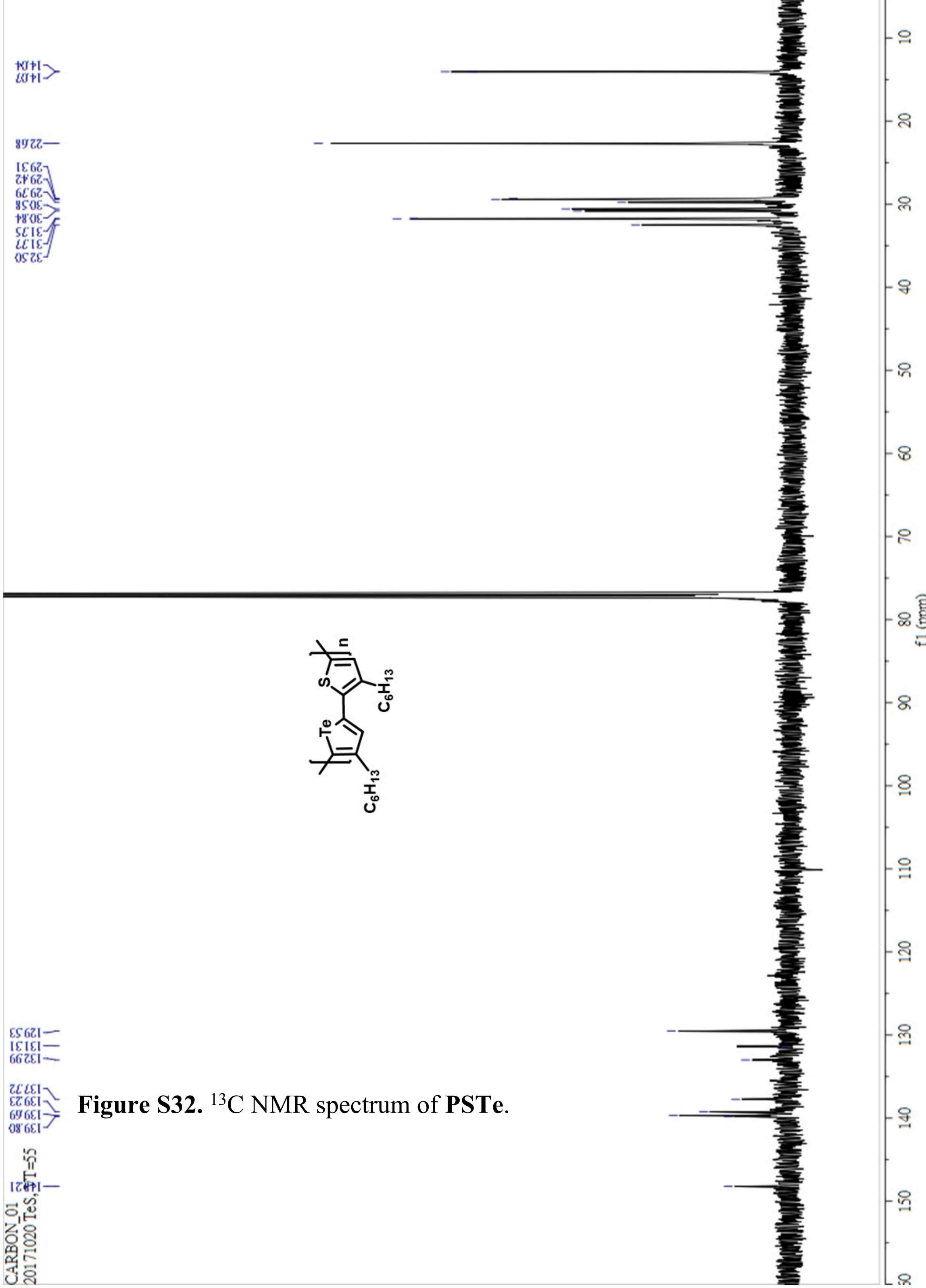

Figure S32. ^{13}C NMR spectrum of **PSTe**.

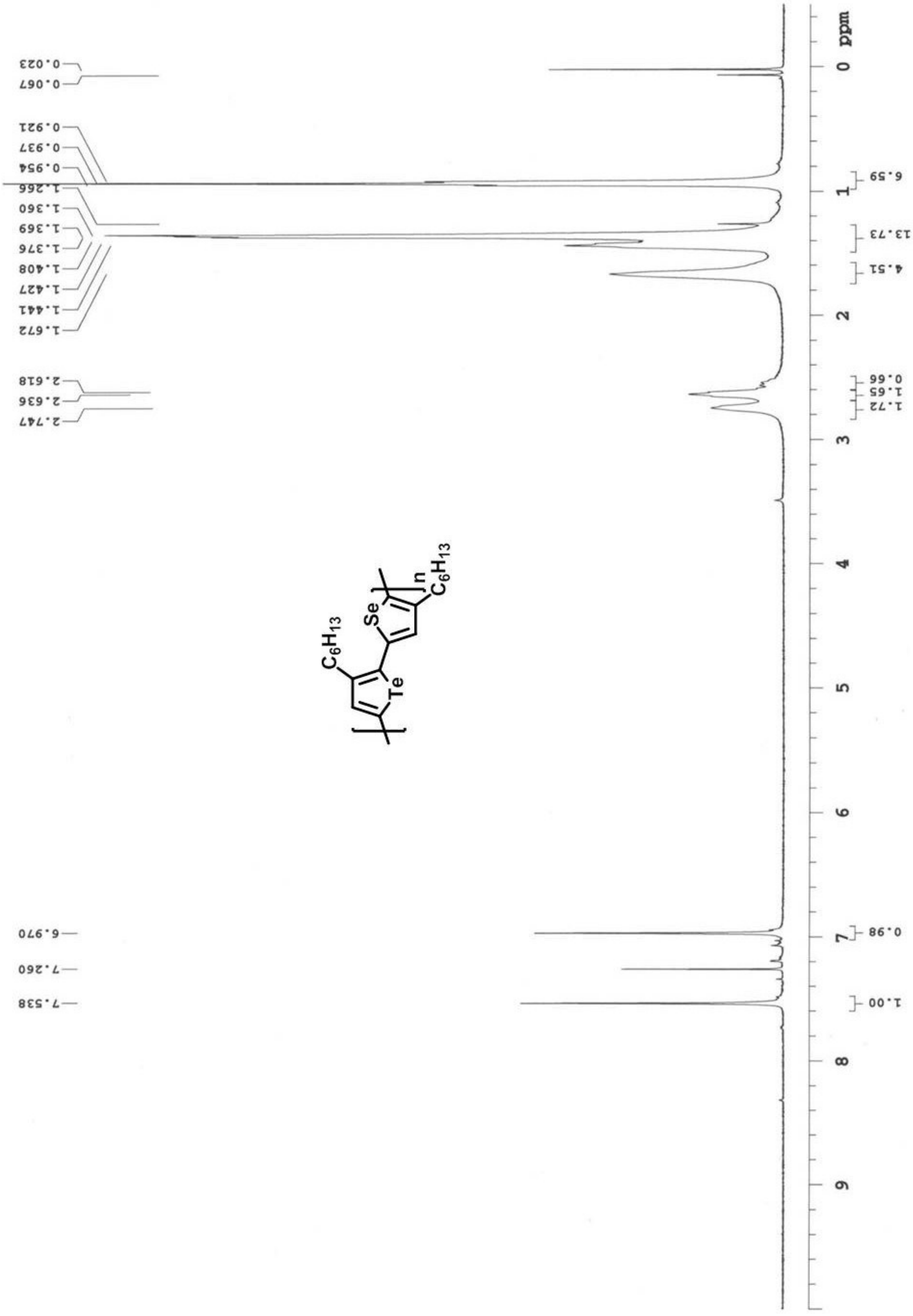

Figure S33. ^{1}H NMR spectrum of **PSeTe**.

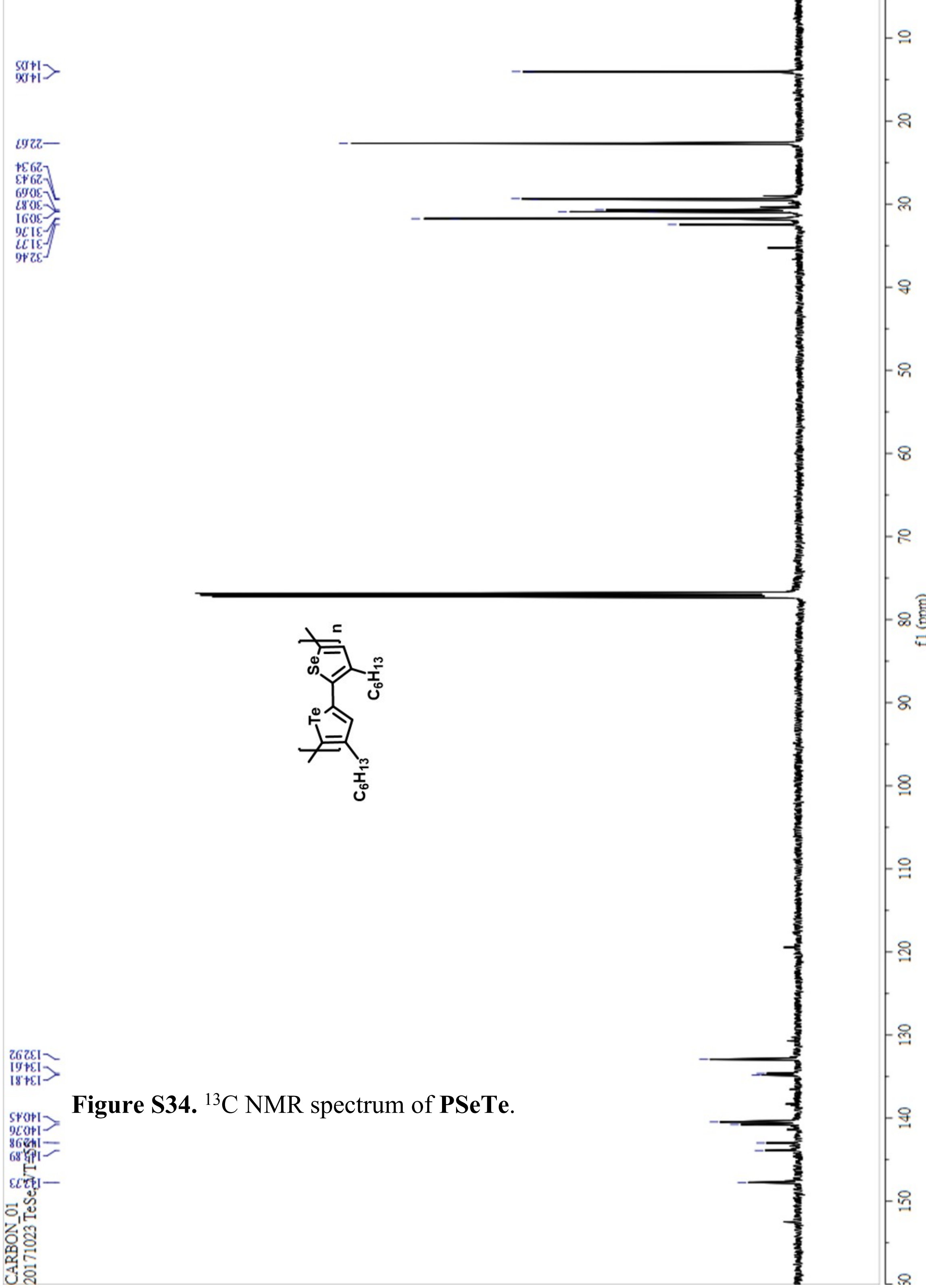

Figure S34. ^{13}C NMR spectrum of **PSeTe**.

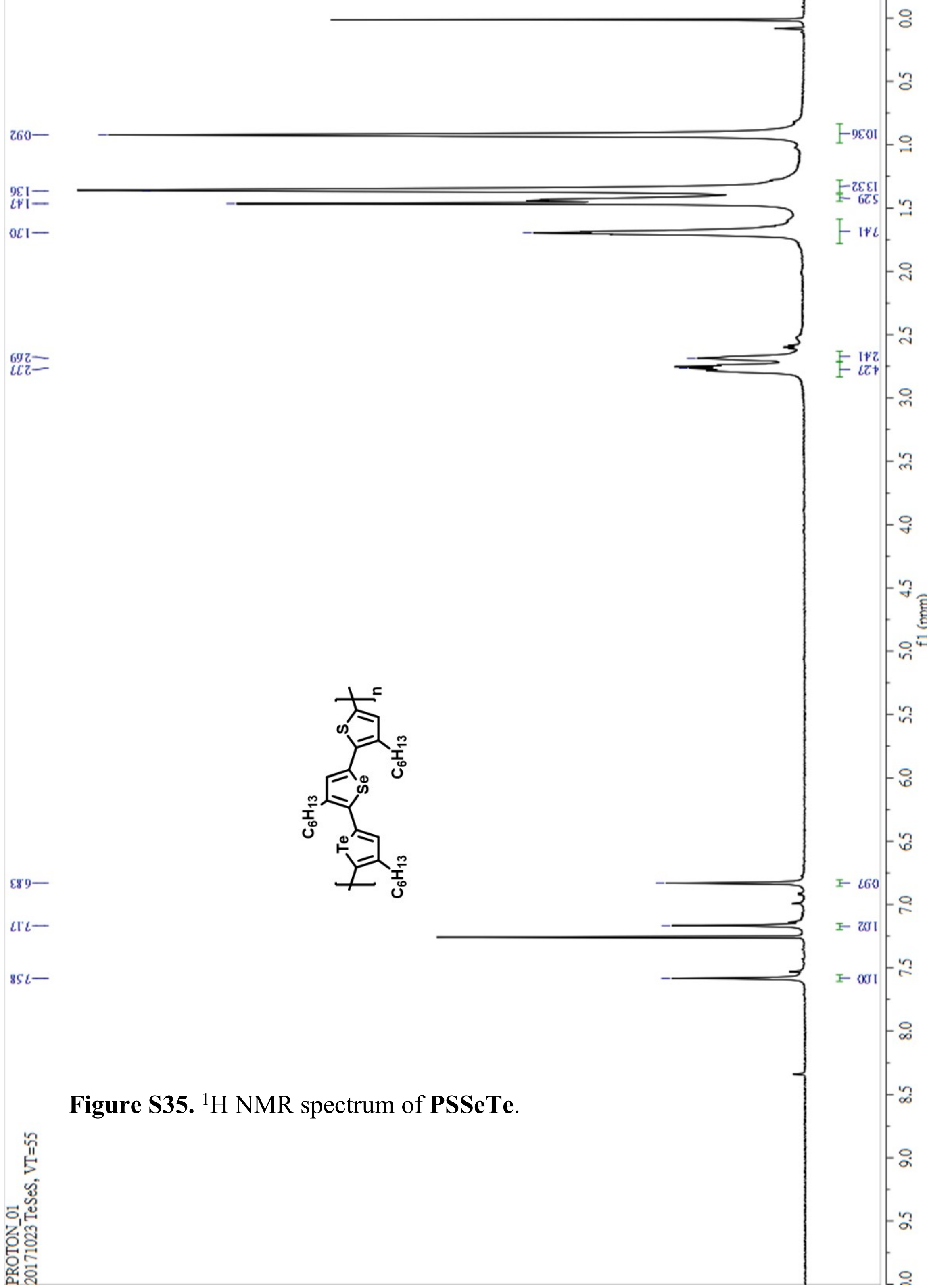

Figure S35. ^{1}H NMR spectrum of **PSSeTe**.

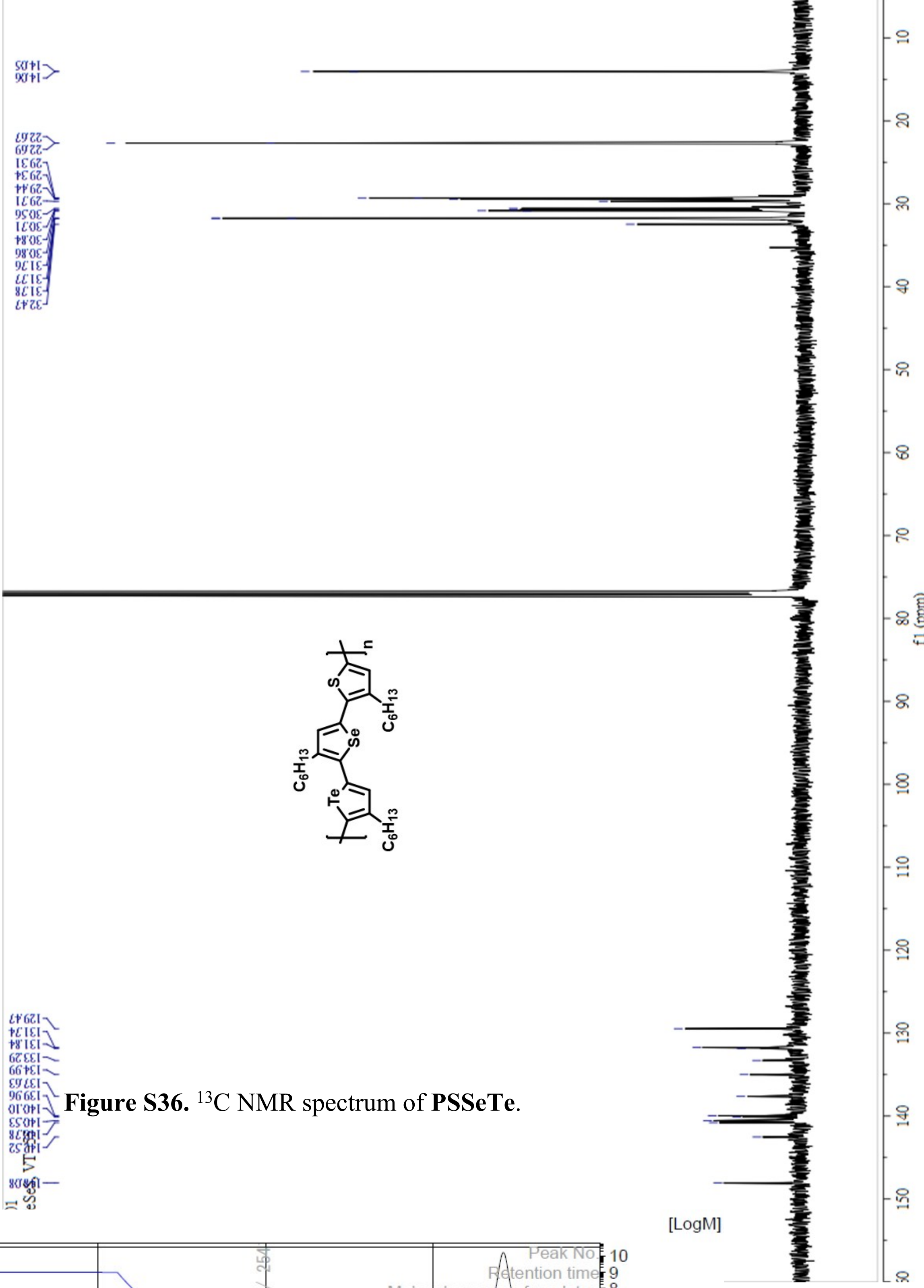

Figure S36. ^{13}C NMR spectrum of **PSSeTe**.

GPC Traces

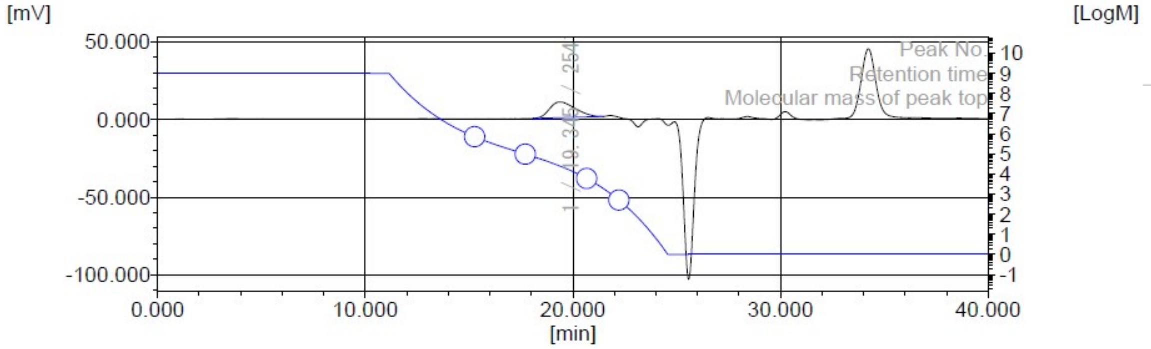

	[min]	[mV]	[mol]		
Peak start	18.110	0.613	71,329	Mn	16,492
Peak top	19.345	11.143	25,419	Mw	22,743
Peak end	21.460	1.817	1,900	Mz	28,327
				Mz+1	32,981
				Mv	22,743
Height [mV]			10.086	Mp	27,123
Area [mV*s]			859.934	Mz/Mw	1.246
Height% [%]			100.000	Mw/Mn	1.379
[eta]			22743.15735	Mz+1/Mw	1.450

Figure S37. GPC chromatogram and analysis of **PSSe** (M/Cat. = 50).

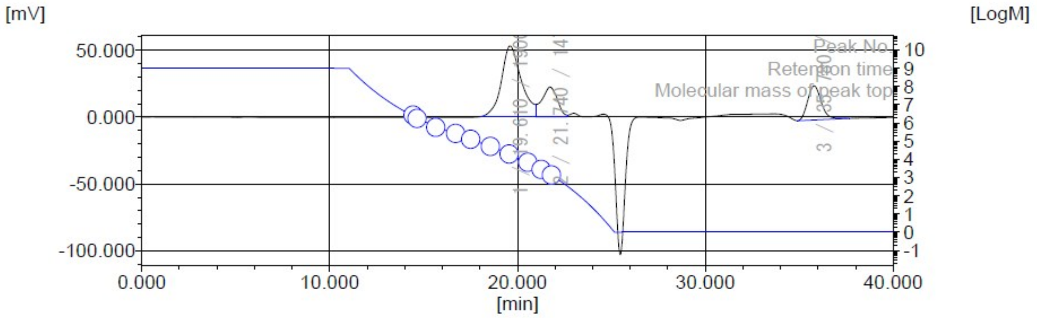

	[min]	[mV]	[mol]		
Peak start	18.055	0.365	77,232	Mn	14,422
Peak top	19.610	53.136	19,007	Mw	19,109
Peak end	20.980	9.391	4,156	Mz	24,236
				Mz+1	29,744
				Mv	19,109
Height [mV]			52.827	Mp	19,577
Area [mV*s]			3887.503	Mz/Mw	1.268
Area% [%]			60.301	Mw/Mn	1.325
[eta]			19109.05046	Mz+1/Mw	1.557

Figure S38. GPC chromatogram and analysis of **PSTe** (M/Cat. = 50).

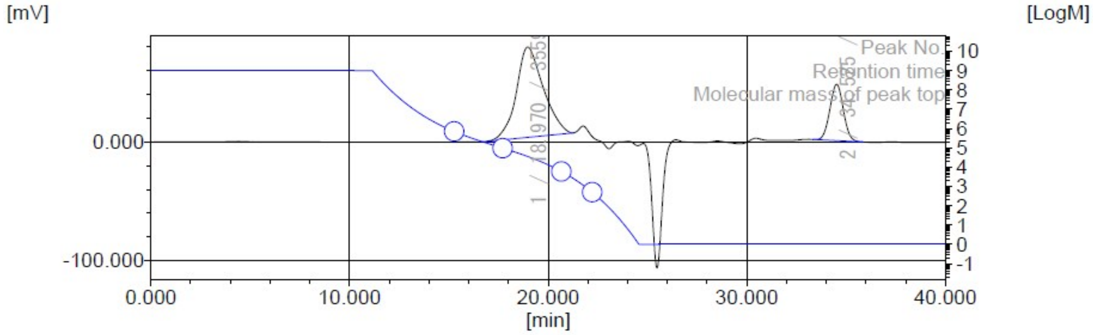

	[min]	[mV]	[mol]		
Peak start	16.940	0.560	170,904	Mn	23,907
Peak top	18.970	80.033	35,597	Mw	35,231
Peak end	21.275	7.588	2,536	Mz	47,010
				Mz+1	59,318
				Mv	35,231
Height [mV]			76.182	Mp	37,483
Area [mV*s]			7206.998	Mz/Mw	1.334
Height% [%]			61.552	Mw/Mn	1.474
[eta]			35231.25895	Mz+1/Mw	1.684

Figure S39. GPC chromatogram and analysis of **PSeTe** (M/Cat. = 50).

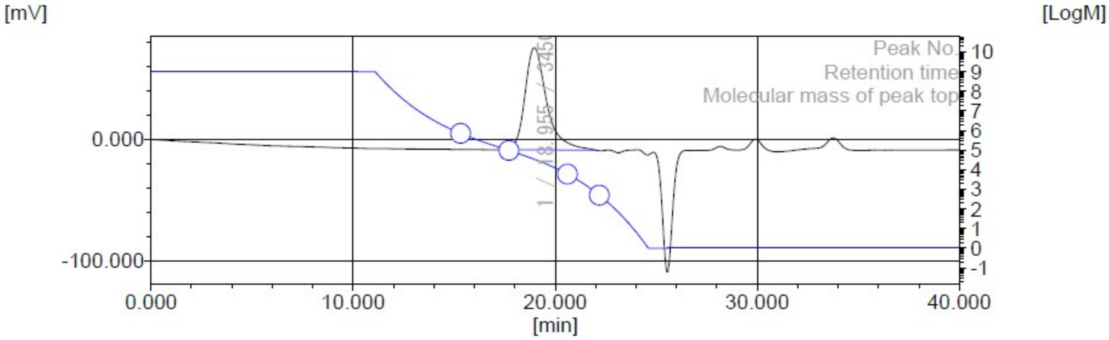

	[min]	[mV]	[mol]		
Peak start	17.220	-8.596	140,314	Mn	20,592
Peak top	18.955	75.662	34,504	Mw	32,327
Peak end	21.985	-8.913	724	Mz	39,818
				Mz+1	46,365
				Mv	32,327
Height [mV]			84.373	Mp	35,438
Area [mV*s]			6313.524	Mz/Mw	1.232
Area% [%]			100.000	Mw/Mn	1.570
[eta]			32326.57493	Mz+1/Mw	1.434

Figure S40. GPC chromatogram and analysis of **PSSeTe** (M/Cat. = 50).

DFT Computation and Analysis

Computational details.

Quantum-chemical calculations were performed with the Gaussian09 suite employing the cam-B3LYP density functional in combination with the LANL2DZ(d,P) basis set for the chalcogens, phosphine, and iodine, the LANL2DZ basis set for nickel, and the 6-311G(d,p) basis set for the remaining atoms. Geometry optimizations were performed with tight SCF and convergence criteria and an ultrafine integration grid, applying the GEDIIS optimization algorithm. The nature of each stationary point was confirmed by a frequency analysis.

▪ 3-Hexyl-2-(4-hexyl-5-iodo-selenophen-2-yl)-5-iodo-thiophene

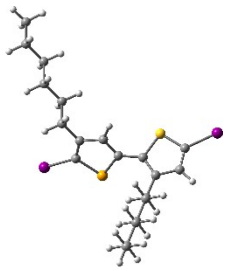

C	2.245111877258	1.014266772530	-0.847914146666
C	2.164105067687	0.359065122688	0.340945918701
C	0.861144734280	-0.191349123933	0.584402291982
C	-0.089494763217	0.017151855755	-0.361924668779
H	0.640366469535	-0.735840763442	1.495249250091
C	-1.462595036652	-0.482448210242	-0.359709826870
C	-2.649336795618	0.196069071859	-0.426670762731
C	-3.776515847383	-0.681905304343	-0.391457767545
C	-3.438018342415	-1.992331300236	-0.300937693647
I	3.916656667053	1.985101234792	-1.620327103300
I	-4.738196791028	-3.606683779194	-0.217147908423
C	3.288077783629	0.170587082632	1.320375615693
H	3.995486948975	0.998654874408	1.236345992087
H	2.881460109646	0.209410388717	2.335561190996
C	4.031943269486	-1.154846301063	1.122346659616
H	4.436962913011	-1.186591243538	0.105827672992
H	3.320086417979	-1.984164768518	1.194483903218
H	-4.799829399433	-0.334159440130	-0.429216148277
C	-2.804650623827	1.691132533370	-0.452772437162
H	-1.859240484269	2.160828677891	-0.729173470192
H	-3.527062427079	1.962470139150	-1.229745155240
C	-3.270803278443	2.261797535225	0.891613441318
H	-2.540432405336	1.993732239817	1.661889102278
H	-4.212767850172	1.785897356740	1.184684158810
Se	0.634837006907	0.986267660903	-1.807212911481
C	5.158080290178	-1.357534313159	2.130836726351
H	5.865614775559	-0.523506478269	2.055303749994
H	4.747759594097	-1.317563881571	3.146961882944
C	5.907294953074	-2.672841580012	1.941407974251
H	5.199963998008	-3.507325434060	2.016099090452
H	6.317206838473	-2.712946512941	0.925377810434
C	7.036142519325	-2.879998413161	2.947093427489
H	6.626049173873	-2.839830457717	3.962085966232
H	7.742597447038	-2.046253685308	2.871781204986
C	7.778599074956	-4.197047579936	2.748860612972
H	7.102141510234	-5.049714562662	2.851521872724
H	8.226666373376	-4.250274591627	1.753109142483
C	-3.452626531820	3.775881922375	0.859487750079
H	-2.507803722774	4.247464447825	0.565237373914
H	-4.178813557151	4.037786571778	0.080721617702
C	-3.912324376921	4.357069018722	2.192971915888
H	-4.857323160716	3.885590257341	2.488046846206
H	-3.186571276522	4.094928543788	2.971850532115
C	-4.094218493870	5.871925374891	2.165482537448
H	-4.819148886588	6.133382295613	1.386978641950
H	-3.149796768709	6.342314232834	1.871253595287
C	-4.553676986395	6.442939244036	3.502639372265
H	-4.675637234408	7.527035554763	3.454797871283
H	-5.512513973010	6.013847279235	3.805499077493
H	-3.830775653861	6.224628289139	4.293154130262
H	8.580080911221	-4.319388011710	3.480380345916
S	-1.720408656148	-2.212869320444	-0.262827034119

3-Hexyl-2-(4-hexyl-5-iodo-tellurophen-2-yl)-5-iodo-thiophene

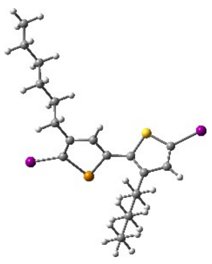

C	2.589986013958	0.924787331284	-0.666955046670
C	2.329648340393	0.272140754498	0.494142085521
C	0.952571634589	-0.116167265007	0.685468121416
C	0.017719656443	0.190550098552	-0.246134421665
H	0.667255814951	-0.642667219155	1.591058375976
C	-1.390989795672	-0.195377155111	-0.199311874568
C	-2.522637284713	0.574927090491	-0.218149520871
C	-3.714694280267	-0.210167820587	-0.147430012779
C	-3.480005634730	-1.544431292122	-0.079760882414
I	4.459909551600	1.640266208108	-1.262631090524
I	-4.902142785927	-3.050502215348	0.034916961433
C	3.351003808015	-0.085815174921	1.540464638211
H	4.170288596572	0.635820963215	1.522700478663
H	2.886063592809	-0.006468304426	2.527942841673
C	3.914950293295	-1.499242713485	1.357812651043
H	4.378935452419	-1.571382560595	0.368994799353
H	3.092057669143	-2.222181496578	1.363802434147
H	-4.707238066201	0.219439058041	-0.144684031077
C	-2.559402984571	2.077638759654	-0.224058625632
H	-1.607957226602	2.475587933930	-0.581813140224
H	-3.325684370590	2.414808899367	-0.929882911740
C	-2.852898070242	2.668206173415	1.160096419690
H	-2.077676143719	2.334901502532	1.857522770830
H	-3.798189957875	2.262759436919	1.536594742045
Te	0.912569253833	1.168170369801	-1.845010123792
C	4.931980708415	-1.872719709297	2.431396413388
H	5.751295262105	-1.144361482131	2.422066840791
H	4.463777734435	-1.791690897709	3.419672066981
C	5.503579733056	-3.276509050873	2.258304690138
H	4.684521785049	-4.005435926993	2.266793073103
H	5.971413145839	-3.357631734952	1.270121396035
C	6.522900931458	-3.654108423686	3.329271985735
H	6.054929443072	-3.572848941246	4.316388415125
H	7.340927506213	-2.925665322184	3.320109842694
C	7.088511693989	-5.058430847041	3.147160710061
H	6.295656563507	-5.810185629898	3.184921907718
H	7.591038778548	-5.158159473993	2.181419812133
C	-2.917398813433	4.192059486188	1.151722916932
H	-1.969878319088	4.592070896267	0.772368321937
H	-3.690257190282	4.518951324485	0.445795832941
C	-3.203598980407	4.791503440974	2.525078800172
H	-4.150940968440	4.391371083507	2.905616123130
H	-2.431031659063	4.464615398361	3.231002852302
C	-3.267838555641	6.316122327090	2.521592716804
H	-4.039900162536	6.642310483760	1.816254393204
H	-2.321187917410	6.715123527205	2.141556811760
C	-3.553471807059	6.905306740916	3.898704345338
H	-3.593943252437	7.996094801292	3.867398569098
H	-4.510312654063	6.548468715453	4.289013114337
H	-2.778874968698	6.622191053261	4.616335416970
H	7.814371542392	-5.302305424939	3.925707260672
S	-1.784942656436	-1.901150776289	-0.104492267539

3-Hexyl-2-(4-hexyl-5-iodo-tellurophen-2-yl)-5-iodo-selenophene

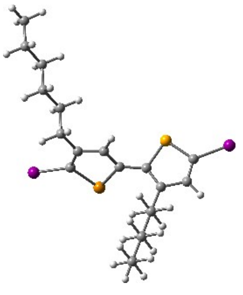

C	-3.053546601961	-0.129508424419	-0.143181949108
C	-2.448415145328	-0.940832817302	-1.047271096265
C	-1.013773970596	-0.802344867113	-1.134377441584
C	-0.363726917257	0.102402843235	-0.363689271577
H	-0.458854288807	-1.419099117212	-1.834799725496
C	1.081636386579	0.312956954233	-0.317675410306
C	1.811110795420	1.408963630685	-0.676255704831
C	3.227502596474	1.263804784324	-0.494327357235
C	3.636557324402	0.075103804229	0.005021651315
I	-5.106089290918	-0.060112992527	0.239810551620
I	5.605108634572	-0.467301201139	0.388438213397
C	-3.146376289008	-1.959267397754	-1.908607884146

H	-4.173996234621	-1.643255128397	-2.099413051307
H	-2.646311568006	-1.999814619204	-2.881183556754
C	-3.153253248644	-3.358664781118	-1.283561849310
H	-3.654006664730	-3.310869757005	-0.311338368441
H	-2.122942645341	-3.671886357016	-1.082833548424
H	3.918934129980	2.058315433968	-0.745098617433
C	1.237242946367	2.658202019755	-1.288611861848
H	0.170774244348	2.727713099957	-1.066584857205
H	1.711774756400	3.532490341595	-0.830641138690
C	1.432744275700	2.715709625576	-2.808112173594
H	0.946667271453	1.844669963830	-3.259251066518
H	2.498877184235	2.627253048576	-3.043426278659
Te	-1.721813700851	1.083291212692	0.865598049007
Se	2.195171878189	-1.071735367718	0.327135983848
C	-3.840643913177	-4.396223034855	-2.165292461174
H	-4.870158690018	-4.076209484182	-2.364103544095
H	-3.339929388456	-4.434082592896	-3.140132285942
C	-3.856536269395	-5.793160865291	-1.552449246357
H	-2.827081258765	-6.113792690750	-1.353140517651
H	-4.356425274575	-5.755128002332	-0.577450506703
C	-4.545579390245	-6.834179499862	-2.430010636711
H	-4.045816053430	-6.871676326509	-3.404088297878
H	-5.573975297388	-6.513442866322	-2.628476810353
C	-4.556590655974	-8.226623536265	-1.808600199509
H	-3.539730801145	-8.585978933403	-1.629576763490
H	-5.079058205663	-8.224961602711	-0.848311159327
C	0.877261383619	3.993791576364	-3.428196251779
H	-0.188924733150	4.078521705090	-3.187861266110
H	1.363136535036	4.862008204751	-2.967122812368
C	1.059664148134	4.059147319044	-4.941382218487
H	2.125920176861	3.973787795644	-5.182622436901
H	0.574090494012	3.190990914435	-5.402483753647
C	0.504211811174	5.335722572233	-5.566253434364
H	0.989658280818	6.202853215550	-5.105388249919
H	-0.560911474960	5.420253363014	-5.325110503669
C	0.690792434063	5.390901746524	-7.078631962465
H	0.285021154871	6.313372834353	-7.499157409314
H	1.749457032185	5.341065465080	-7.346899029615
H	0.187222983485	4.553094809302	-7.568186097371
H	-5.055156575969	-8.950707620738	-2.456368905260

3-Hexyl-5-(3-hexyl-5-iodo-selenophen-2-yl)-2-iodo-thiophene

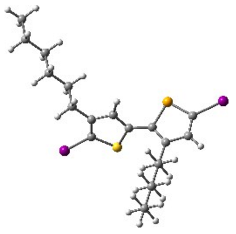

C	2.096932573068	0.912704465657	-0.827197553938
C	2.068105494431	0.292326307600	0.386638532727
C	0.779285241790	-0.271815135093	0.638242262151
C	-0.137161844175	-0.073113923086	-0.348979074673
H	0.539924513791	-0.801334064479	1.551826696656
C	-1.516781793931	-0.552302233269	-0.410537246009
C	-2.677163396293	0.159230339020	-0.500990253127
C	-3.862336219519	-0.649520295137	-0.540789904151
C	-3.663059724767	-1.986292409481	-0.483870091885
I	3.700078541814	1.910437030952	-1.695091353173
I	-5.150022298955	-3.436762500590	-0.509439829720
C	3.214849304713	0.164062544948	1.346194494431
H	3.893839146064	1.011442434817	1.224834268741
H	2.828229123648	0.218330685053	2.368396182426
C	3.996967009754	-1.142113249708	1.168296128681
H	4.380271967472	-1.190329864593	0.143992867113
H	3.313758833488	-1.990908332878	1.280078682096
H	-4.849983763862	-0.211368023520	-0.608235687413
C	-2.772231705827	1.661461087562	-0.486469812435
H	-1.800413348301	2.100607783944	-0.715984928319
H	-3.454775475358	1.983145129259	-1.280126641919
C	-3.265259287477	2.209728304974	0.857538169401
H	-2.573799394538	1.891129635207	1.644361933697

H	-4.234672063972	1.763137648281	1.103516223136
C	5.151103645399	-1.280098892352	2.155810581383
H	5.829988037812	-0.427111041404	2.039954975860
H	4.762462503606	-1.222902272997	3.179608605077
C	5.937247909136	-2.576506731921	1.986973557318
H	5.258735484746	-3.430014996039	2.102888659587
H	6.324827205514	-2.634233070036	0.963052887691
C	7.094937864231	-2.718303186190	2.971005730148
H	6.707253998779	-2.660316182059	3.993910712512
H	7.772709807623	-1.865736120845	2.854338487390
C	7.873950575247	-4.017089893642	2.793753727608
H	7.227388322158	-4.886839759563	2.937530028890
H	8.300521345006	-4.086313429115	1.789582540194
C	-3.388044167187	3.730135357786	0.865320226514
H	-2.416488965522	4.172652863116	0.616454467984
H	-4.076787967724	4.042621889629	0.071379085088
C	-3.870101164197	4.290204354908	2.199922697584
H	-4.841770571705	3.847698946545	2.449801672481
H	-3.181540266358	3.977676417679	2.993860587372
C	-3.993156161836	5.811173920417	2.211960916061
H	-4.681448926661	6.122918575481	1.418865464230
H	-3.022288207211	6.252549469000	1.962173017128
C	-4.474440424582	6.361235494186	3.550214905346
H	-4.553333123344	7.450198674668	3.530900107648
H	-5.458428555717	5.961479447081	3.809417100182
H	-3.786914093826	6.092308639981	4.356543509376
H	8.695485111631	-4.092513565173	3.509179898646
S	0.582117202707	0.813494992005	-1.672675748608
Se	-1.845909850785	-2.411243266586	-0.376607465182

3-Hexyl-5-(3-hexyl-5-iodo-tellurophen-2-yl)-2-iodo-thiophene

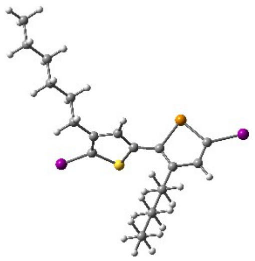

C	1.968293064440	0.734916510093	-0.780423377180
C	1.863319677758	0.137102705835	0.440155512963
C	0.548626621381	-0.389813930605	0.637093983337
C	-0.313874321204	-0.184408488132	-0.395822096879
H	0.251889454265	-0.899393691532	1.545288239753
C	-1.706434559150	-0.616472682029	-0.520064139837
C	-2.822601204765	0.162022357009	-0.546118597776
C	-4.088680040561	-0.517548306270	-0.683098712919
C	-4.090736628400	-1.864215493811	-0.777434398154
I	3.641311290632	1.667121607365	-1.587991213402
I	-5.813806858436	-3.019146319091	-0.973429636813
C	2.957930208581	-0.004933269453	1.456975818509
H	3.669057548328	0.817668247230	1.350301516626
H	2.524584537189	0.085564294871	2.457700298308
C	3.705403570133	-1.338555897866	1.347050870193
H	4.135238191346	-1.423746468521	0.343832026831
H	2.990928649758	-2.162986733164	1.445609703321
H	-5.009934397788	0.053553981436	-0.706555888439
C	-2.810098293916	1.659837895333	-0.374011201365
H	-1.810894350244	2.050694697085	-0.570363756368
H	-3.474426599486	2.109450252047	-1.119591923888
C	-3.253784909224	2.095646378562	1.027115649345
H	-2.578537280960	1.650984122699	1.765368890588
H	-4.249133990704	1.691330874830	1.240722724230
C	4.806303480724	-1.487515667871	2.392055659678
H	5.516726007260	-0.658941665776	2.289020893976
H	4.371419175644	-1.392627866011	3.394223167832
C	5.558087039118	-2.811189710718	2.292261916779
H	4.848205758743	-3.640462141207	2.395895363066
H	5.991757493792	-2.906744901370	1.289872961965
C	6.662804188720	-2.962994297984	3.333983440105

H	6.229144901854	-2.866946840239	4.335328953957
H	7.371970430595	-2.134749012994	3.229470193889
C	7.407858300881	-4.289024976113	3.225925548578
H	6.728040143675	-5.134723656323	3.359495568141
H	7.879115580221	-4.397007065619	2.245424815777
C	-3.273038432598	3.611651916836	1.195344216554
H	-2.275945018868	4.012174087654	0.978498234785
H	-3.946060686565	4.050999670005	0.449466374558
C	-3.704207715646	4.058958550970	2.588649700920
H	-4.701359717763	3.658131418236	2.806348196053
H	-3.031415775789	3.619507634205	3.334574957960
C	-3.723814649058	5.574999052742	2.761502464437
H	-4.396316254326	6.013567067278	2.016304207465
H	-2.727507821213	5.974752678128	2.543867035586
C	-4.155047472664	6.011845470308	4.157342460328
H	-4.160047177189	7.099613113929	4.253216376301
H	-5.161597373428	5.653374252941	4.388791044558
H	-3.480384891746	5.614302358237	4.920225967104
H	8.191811664951	-4.371003755390	3.981654807618
S	0.492317701181	0.663859283852	-1.695818763238
Te	-2.180770259479	-2.631493641625	-0.704452055716

3-Hexyl-5-(3-hexyl-5-iodo-tellurophen-2-yl)-2-iodo-selenophene

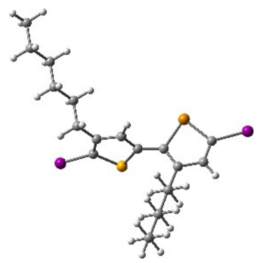

C	2.341720467166	0.750267112518	-1.333394179000
C	2.168012090309	0.460482062904	-0.016581674099
C	0.826172720367	0.050340038739	0.291076078545
C	-0.066547028718	0.019802058406	-0.730268606721
H	0.533885780524	-0.211026073903	1.301753865534
C	-1.472956630473	-0.378912998255	-0.682860236278
C	-2.568152863204	0.424779897890	-0.768593810671
C	-3.858470575775	-0.218074176319	-0.697215051583
C	-3.902771797117	-1.559855928282	-0.555749966379
I	4.103966584454	1.383773249918	-2.243846083863
I	-5.665336839849	-2.664290139255	-0.432437181566
C	3.232072892929	0.509984093056	1.043436927021
H	3.990993413951	1.247176505677	0.771935781358
H	2.781857264209	0.855870044608	1.978983637782
C	3.904095319634	-0.847704871313	1.276194295128
H	4.352625149232	-1.187879683397	0.337303459904
H	3.141685834470	-1.589299020067	1.538353410601
H	-4.763466345894	0.375954176229	-0.757212052223
C	-2.498846731740	1.927360227359	-0.867787859919
H	-1.514040349642	2.231707590299	-1.225815651162
H	-3.224414395315	2.269421867095	-1.613363724679
C	-2.782996912975	2.621005617248	0.469350219664
H	-2.045753853417	2.283685222944	1.205098833740
H	-3.760574248877	2.303397193151	0.848041041689
C	4.968864318598	-0.801210725564	2.367265601829
H	5.727354332637	-0.056027132559	2.100367346298
H	4.515553512693	-0.452447801984	3.302861717353
C	5.646704492136	-2.146743942644	2.606680268144
H	4.888553721404	-2.892732025561	2.873296669853
H	6.099613401462	-2.495636404915	1.671195734853
C	6.714141059529	-2.104428193763	3.696360166949
H	6.261154343228	-1.755654642246	4.630782274514
H	7.471360974446	-1.359335747766	3.429349819963
C	7.385704076442	-3.453743765585	3.927650455603
H	6.656016167578	-4.210497035887	4.227798643970
H	7.875202028291	-3.810901685943	3.017729266966
C	-2.746296842447	4.142373136617	0.364992239780
H	-1.768035762507	4.454748081819	-0.018510506063
H	-3.483395753211	4.473138214540	-0.376370477884
C	-3.015776095819	4.845353248443	1.691819416297
H	-3.993657961085	4.532323505621	2.076712503394
H	-2.278433846190	4.515055503220	2.433043277362
C	-2.979558492937	6.367610957607	1.591601214110

H	-3.716926450708	6.697231558983	0.851628605337
H	-2.002723325656	6.679563938724	1.206539053264
C	-3.248195909241	7.060792540189	2.922947997067
H	-3.216446756093	8.147695648887	2.822526716880
H	-4.232617971769	6.792089855779	3.315295451741
H	-2.505643802237	6.774714721648	3.672602367755
H	8.144059245940	-3.394947568637	4.711154517592
Te	-2.013135830570	-2.376451531584	-0.478343333210
Se	0.776975351839	0.522324605311	-2.341996102542

Ni(dppp)

Ni	-0.354550527871	-0.146600764045	-1.879340929270
P	1.596347167042	-0.276761084107	-1.066220068334
P	-1.878179947338	-0.004473392122	-0.415625742574
C	1.198770621540	-0.951852126218	0.641432438762
C	-0.889836806282	0.687116495428	1.024781387191
H	2.120920239500	-1.201829819714	1.174545124703
H	0.718233642844	-1.906196621507	0.412748710148
H	-1.556447723215	0.944245289311	1.853378214530
H	-0.526699887704	1.638174407574	0.627785176841
C	2.902259302511	-1.505138524126	-1.569970276294
C	4.246387607831	-1.418615427330	-1.212147581398
C	2.483827513539	-2.587476445783	-2.342827710665
C	5.146958087300	-2.396373758769	-1.611573961202
H	4.600354196942	-0.577249965365	-0.628938703403
C	3.380370138857	-3.571800952947	-2.732476108095
H	1.444108417908	-2.647063623290	-2.646310127439
C	4.715806766525	-3.476928529550	-2.368239727976
H	6.190714336687	-2.311966489334	-1.331918059535
H	3.037311903712	-4.407779038359	-3.330726318644
H	5.420686724756	-4.238789125045	-2.679559609383
C	2.581065062881	1.257323107449	-0.724535177778
C	2.625675870687	2.231781591015	-1.721141483605
C	3.257652121004	1.497293395613	0.471064377186
C	3.342091244092	3.405186258798	-1.538020986008
H	2.085096736272	2.064812773198	-2.646097966020
C	3.966150544105	2.676169153910	0.661498952710
H	3.237182488742	0.761239360574	1.265744175205
C	4.012743205726	3.630787686680	-0.344015670286
H	3.369113232663	4.148961110811	-2.325606255157
H	4.483679332001	2.848099014466	1.598077263074
H	4.565937271487	4.550537835383	-0.195503812971
C	-2.669146511158	-1.533122267932	0.275342779339
C	-3.070198772432	-2.517536281650	-0.627358603797
C	-2.865802859668	-1.759669930517	1.637259320362
C	-3.669281395243	-3.687646059167	-0.185085221118
H	-2.902042429259	-2.360983329706	-1.687011150877
C	-3.454844800302	-2.935191871290	2.083534067609
H	-2.560007454159	-1.015646415070	2.362951245075
C	-3.861010898615	-3.899853349299	1.173048242793
H	-3.978596934901	-4.439305548707	-0.901711798257
H	-3.597296575493	-3.096631987090	3.145710817506
H	-4.321000393373	-4.816986404310	1.521324542106
C	-3.280186234699	1.221245992139	-0.424091310243
C	-4.402983621716	1.140936287821	0.397541598662
C	-3.172446707495	2.294892303193	-1.306998404468
C	-5.388932355668	2.116327311379	0.341887048157
H	-4.519851684848	0.306204100976	1.077898094297
C	-4.151109103606	3.276940443545	-1.355067449980
H	-2.313597662820	2.349403112509	-1.967358058681
C	-5.263465226982	3.188330198581	-0.530523980541
H	-6.260135069580	2.036764023319	0.981757836489
H	-4.049850360230	4.106153834683	-2.045432143864
H	-6.034688710094	3.948351354945	-0.572826924693
C	0.294883193585	-0.128451319836	1.581397895801
H	0.920182784271	0.578875885438	2.133087657052
H	-0.086535030260	-0.828974896550	2.329368356957

S

C	0.884574669962	-3.285055145343	-1.926223842531
C	1.622017233896	-2.166834463187	-2.160553113166
C	0.788334051582	-1.022199923616	-2.371338426615
C	-0.536866590392	-1.298261531513	-2.291928765698
S	-0.819783838325	-2.972795702564	-1.956580530937
H	-1.366765954365	-0.618767672564	-2.405028960690
H	1.175347993165	-0.030757036574	-2.568325341319
C	3.123795796664	-2.127629693978	-2.217122279561
H	3.522322121045	-3.064294401284	-1.820072892968
H	3.487360272388	-1.333938053207	-1.556837289179
C	3.664348334080	-1.898762824932	-3.630639591809
H	3.347226057721	-2.700001194845	-4.301091755239
H	3.299854066727	-0.956760831533	-4.045978217859
H	4.756018883686	-1.865167446939	-3.631393041232
H	1.247980492167	-4.282733197921	-1.733251471197

Se

C	0.915396177174	-3.324159999280	-1.921425147738
C	1.617912699602	-2.188408964821	-2.155676085885
C	0.788420031617	-1.033847095054	-2.368541798208
C	-0.544240459813	-1.251473265687	-2.304032362140
Se	-0.931147059030	-3.042392108998	-1.947681540571
H	-1.330780746088	-0.523397436518	-2.427420461280
H	1.205705568538	-0.052455476230	-2.560879291700
C	3.120582750711	-2.124288505098	-2.214539474318
H	3.535725472006	-3.051366236717	-1.812368502553
H	3.470881919017	-1.319779474237	-1.559970641442
C	3.654510873528	-1.895196007883	-3.630540864221
H	3.350433755831	-2.706617215465	-4.294764897514
H	3.272985840185	-0.962717390862	-4.052068798193
H	4.745441224633	-1.842354982598	-3.633486544941
H	1.323935542088	-4.305504960553	-1.732969109298

Te

C	0.953958774611	-3.376009588693	-1.915955420552
C	1.612109910603	-2.216735311620	-2.149366100874
C	0.787919964236	-1.049412904592	-2.364021107633
C	-0.552954067835	-1.191716316254	-2.319396120970
Te	-1.088337888742	-3.139128531079	-1.937738134723
H	-1.270206986731	-0.396227713093	-2.456059522602
H	1.244708980242	-0.082259566053	-2.548579330211
C	3.115359015157	-2.118555140031	-2.210921588181
H	3.552365903650	-3.031599814556	-1.800362696017
H	3.447095975551	-1.298724147808	-1.565429010024
C	3.640957997778	-1.891555945778	-3.630423123812
H	3.355846936750	-2.717781782678	-4.284749465407
H	3.235998788529	-0.973631593921	-4.061970297585
H	4.730288780331	-1.811890896065	-3.636129577132
H	1.430651505870	-4.328729867777	-1.735264024275

complex 1

Ni	3.797812605974	-0.017469533908	0.532404320786
P	4.060999073649	1.743568023722	-0.784247063315
P	4.847766473136	-1.491591362681	-0.741749010642
C	4.417642982867	1.183331190371	-2.530328364243
C	6.183531570170	-0.601830830625	-1.700028860650
H	4.363349416600	2.023812893599	-3.226138243006
H	3.559353270067	0.546747763434	-2.762571610277
H	6.880587960225	-1.314322736513	-2.146800182249
H	6.743903525600	-0.081841254022	-0.918389796052
C	2.545998254082	2.761640936914	-1.101831872156
C	2.563745327778	4.123667023803	-1.394238009585
C	1.320338964481	2.096865950916	-1.054171974578
C	1.379826394113	4.804293311628	-1.643912853839
H	3.502823417793	4.662867356487	-1.416860137273
C	0.139631396357	2.775163703565	-1.317383971788
H	1.291954290488	1.046563857102	-0.786266453168
C	0.166936561848	4.131115988967	-1.611811878716
H	1.406508152430	5.865172899861	-1.863724816828
H	-0.804695555744	2.245253791309	-1.277851619901
H	-0.755902318809	4.664182691214	-1.807613428239
C	5.377797441168	2.995297507828	-0.434922456723
C	5.700605760852	3.237689228465	0.899918085030
C	6.066264623794	3.693882838753	-1.426567028409
C	6.674653515801	4.166150218896	1.237372803689
H	5.181837383027	2.686454333602	1.675326971410
C	7.048219206626	4.615753154776	-1.090919558004
H	5.837785601458	3.524726270976	-2.471918573731
C	7.351997259671	4.855595448829	0.241691480582
H	6.910072262210	4.345754272437	2.279639409215
H	7.576039055554	5.148922243613	-1.872877228412
H	8.118126981160	5.575750411906	0.503106288461
C	3.941127687621	-2.464376919253	-2.024450530099
C	2.604301552468	-2.771520920580	-1.771105387808
C	4.514958247737	-2.888482147825	-3.223224802962

C	1.861942447318	-3.497695555042	-2.692109626140
H	2.145288215107	-2.437655658022	-0.846751905214
C	3.769186645008	-3.605872124297	-4.147726094513
H	5.552669320669	-2.664392868382	-3.440567467280
C	2.441740962791	-3.913395387049	-3.881972577734
H	0.825826032649	-3.733090295446	-2.479955941578
H	4.226144106654	-3.927195011898	-5.076353716352
H	1.860108569470	-4.474631881643	-4.603683381475
C	5.877005778468	-2.760187084479	0.134542014767
C	6.037603623427	-4.074270572115	-0.297529741424
C	6.521525949855	-2.354251419703	1.304160492145
C	6.835028421950	-4.958099191453	0.418166109379
H	5.531893952534	-4.417592799602	-1.191260511813
C	7.329067578872	-3.232396079384	2.010174630755
H	6.375575229105	-1.342978900281	1.668573108496
C	7.486525534303	-4.539081236897	1.568387159606
H	6.944944521841	-5.979718369451	0.073699603408
H	7.825274142008	-2.899924721977	2.914314620033
H	8.108120640674	-5.230541824725	2.124633038438
C	0.775984293028	-0.042952630724	2.107826722944
C	1.678086865531	0.928308810891	2.333954390789
C	3.055303184471	0.440479315235	2.336162151896
C	3.142049247994	-0.962695598063	2.114675704575
S	1.491351331040	-1.641764475351	1.923153766587
H	3.863638543938	-1.622124471160	2.579924514807
H	3.814638230203	0.971312994762	2.902653540815
C	1.331629793057	2.368641180270	2.578451342001
H	0.272322535183	2.526365562566	2.360968688531
H	1.887406318453	2.996249227311	1.874341366027
C	1.629295237574	2.824915310327	4.009426549638
H	1.062527215815	2.233086203239	4.731153859573
H	2.688655349640	2.715343638791	4.251613497279
H	1.364310953723	3.875932333770	4.146620355390
C	5.721659135175	0.404390542023	-2.770456724904
H	5.604386177738	-0.121945977800	-3.721127153583
H	6.539106565618	1.114479951266	-2.919908545819
H	-0.293698553141	0.075486416929	2.012262993430

complex 2

Ni	3.779774897074	-0.037812352798	0.531154940541
P	4.031192071105	1.722326095034	-0.792559412611
P	4.841246416448	-1.505766883567	-0.745870078808
C	4.427271418075	1.170990900503	-2.531973942632
C	6.191720832649	-0.598033657441	-1.666627607959
H	4.380320288483	2.012982475223	-3.226522819135
H	3.578239009961	0.529063825481	-2.782632773984
H	6.907218329123	-1.301741226886	-2.097634797747
H	6.726846968829	-0.071011612567	-0.872189860109
C	2.504484991916	2.715550189943	-1.130137076295
C	2.515671498552	4.052721732418	-1.522416168104
C	1.281186821503	2.058265245880	-1.002659126780
C	1.326677249238	4.715781534492	-1.791440525169
H	3.454236041268	4.586898102984	-1.607358162924
C	0.093994466662	2.719058436312	-1.283648794372
H	1.260173639660	1.030209630718	-0.658980323865
C	0.114538506233	4.049079813938	-1.677921601028
H	1.347763179169	5.757473590942	-2.089593877399
H	-0.849188303434	2.195718356531	-1.180678819423
H	-0.812794515689	4.568570835633	-1.888579542142
C	5.319006645908	2.997071194365	-0.417558657215
C	5.582116100467	3.274622935809	0.923746853278
C	6.040021663269	3.682033223509	-1.395303316916
C	6.528620464671	4.224076873411	1.279686987558
H	5.038666342289	2.734505478413	1.689901528816
C	6.995072555698	4.625024791205	-1.040426475940
H	5.859330702671	3.485608489514	-2.445123161200
C	7.238835774441	4.899930676241	0.297473574504
H	6.717007441993	4.430953742933	2.326450100293
H	7.548718926400	5.147034096256	-1.811988230569
H	7.983773822615	5.636511114141	0.573908332751
C	3.964646635507	-2.456575120085	-2.065573531880
C	2.607728620446	-2.718254937198	-1.879318759524
C	4.584057595841	-2.905195600136	-3.232653554069
C	1.888556045511	-3.423269135900	-2.834948562893
H	2.114124117052	-2.365882035902	-0.980013136306
C	3.862694538664	-3.601855811070	-4.191269725804
H	5.638494771660	-2.716648536128	-3.397346037994
C	2.513592138318	-3.863546207623	-3.992293258959
H	0.835678934500	-3.622713294729	-2.674951208470
H	4.354998253305	-3.942845237463	-5.094452630148
H	1.950849387130	-4.408486333126	-4.740963237608
C	5.852847031239	-2.786321770660	0.132676556052
C	5.967624026314	-4.112248981052	-0.276759648094
C	6.525140298381	-2.379299064607	1.286533603501
C	6.748265737046	-5.006817371448	0.444545499518
H	5.439992235353	-4.456333108281	-1.157274664092
C	7.315958993011	-3.268376514071	1.997281343094
H	6.414046436561	-1.358477502912	1.636212729544
C	7.428333486564	-4.587134703347	1.577548251348
H	6.822410969878	-6.037314479110	0.117431471586
H	7.834308002817	-2.934738115364	2.888466067977
H	8.036894758947	-5.286754556054	2.137952986572
C	0.747982180418	0.075144666891	2.186115953231
C	1.727529221736	0.971195640982	2.387605647661
C	3.085908318333	0.426600860363	2.350669723435
C	3.167474449826	-0.974822729366	2.120208754478
Se	1.383777368982	-1.694613871032	1.939797296931
H	3.893929758083	-1.629916462189	2.584420725014
H	3.867945515831	0.939046193036	2.904841369110
C	1.478748790589	2.431289675630	2.647889601072
H	0.429140182159	2.659463824103	2.447474316376
H	2.064283821170	3.025868490238	1.938889666868
C	1.826010203955	2.856816597027	4.077132848861
H	1.231928801591	2.297640533714	4.802862980317
H	2.879092829245	2.677196564688	4.304318885573
H	1.631189359775	3.921687426840	4.224798403119
C	5.741286893095	0.401372483673	-2.747989749462
H	5.644110557230	-0.129495355142	-3.698375406878
H	6.556366783211	1.116458268846	-2.885650150460
H	-0.309669856521	0.289782919395	2.129652447989

complex 3

Ni	3.750596300596	-0.060084313034	0.533774161347
P	3.983662725692	1.699328501879	-0.798662285663
P	4.830073194134	-1.517857129601	-0.746295614989
C	4.408864589379	1.152723992161	-2.532021120947
C	6.186874518422	-0.582473466149	-1.630382285102
H	4.361028043082	1.993335183237	-3.228129245898
H	3.572083137006	0.499596416328	-2.794235653886
H	6.926695625993	-1.271657376546	-2.043088102807
H	6.691363876782	-0.039700388007	-0.826739075385
C	2.449794698380	2.676802809232	-1.148367622417
C	2.456451387967	3.992203669455	-1.609503289289
C	1.228285410227	2.031588468911	-0.961004789676
C	1.264358877299	4.645069746246	-1.887388668150
H	3.394379789755	4.518257297688	-1.740678770308
C	0.036941989817	2.682475452773	-1.249477438324
H	1.211618767710	1.022658865749	-0.564793727305
C	0.052876916208	3.989713822194	-1.712869402736
H	1.281795061985	5.669805945920	-2.239599287409
H	-0.905144600737	2.168918099075	-1.098603242234
H	-0.877281683470	4.501436524776	-1.930080873377
C	5.255709204058	2.986735919730	-0.410124293875
C	5.505060067573	3.266311746210	0.933379152349
C	5.978107387986	3.679695681446	-1.381308403574
C	6.438416183202	4.225607787386	1.297596331258
H	4.961764595228	2.719741261398	1.694787943262
C	6.919826424542	4.632851019225	-1.018127391244
H	5.809791312894	3.481264684293	-2.432761266072
C	7.149355677080	4.909853731683	0.321813914465
H	6.615738207214	4.433680372283	2.346050530959
H	7.474370323557	5.160918630546	-1.784908567847
H	7.883874344675	5.654371833005	0.604785243838
C	3.997809072953	-2.453923443186	-2.105768814615
C	2.622779846510	-2.659743670201	-2.008112024842
C	4.674718470711	-2.941283121170	-3.224857881607
C	1.939927029982	-3.346487460652	-3.003447161922
H	2.085438166510	-2.279447044368	-1.146243885735
C	3.991267091520	-3.620356318067	-4.222452697544
H	5.744716417173	-2.796971541835	-3.319840525260
C	2.621925183584	-3.825220984424	-4.111848046102
H	0.871529981015	-3.501632418617	-2.911966768603
H	4.528220738091	-3.992052286790	-5.087245585412
H	2.088596636785	-4.356372656438	-4.891328123903
C	5.831479278001	-2.803321953768	0.135996686517
C	5.879365923707	-4.146209252523	-0.228491457110
C	6.554561536422	-2.385870344900	1.255427090324
C	6.644973279677	-5.047503672450	0.501100817866
H	5.312741804507	-4.498177821386	-1.081061776264
C	7.330135125371	-3.282121454312	1.973278146784
H	6.496078815328	-1.350825476306	1.574545949220
C	7.376360021946	-4.618287818375	1.597541281469
H	6.666997196750	-6.090662304806	0.208269290834
H	7.888773912256	-2.939867526386	2.836401053210
H	7.973502276142	-5.322787944336	2.164031745145
C	0.710570797921	0.197309798353	2.280636387068

C	1.766456937196	1.011650179779	2.438851376876
C	3.106153644935	0.415947894574	2.367648436279
C	3.201614720649	-0.986037095308	2.141118794978
Te	1.250405529409	-1.792432175710	1.987178680754
H	3.958722994246	-1.610955301693	2.598937726500
H	3.905669994482	0.924899285721	2.901992099909
C	1.619710217125	2.488853936779	2.698628027622
H	0.586540166310	2.787231252832	2.506480072239
H	2.238395249267	3.040625011101	1.983148352892
C	2.006305383119	2.892106878539	4.124236147218
H	1.379736055027	2.376809936751	4.855057518650
H	3.045776943904	2.640586134033	4.345668265718
H	1.886077205817	3.968180461053	4.270579378094
C	5.736523879918	0.400373491828	-2.726503605427
H	5.658779833767	-0.141282835650	-3.672557319915
H	6.543111557327	1.125680465539	-2.860359103537
H	-0.319758829598	0.525729367282	2.270285072670

References

1. A. A. Jahnke, B. Djukic, T. M. McCormick, E. Buchaca Domingo, C. Hellmann, Y. Lee and D. S. Seferos, *J. Am. Chem. Soc.*, 2013, **135**, 951-954.
2. D. J. Turner, R. Anemian, P. R. Mackie, D. C. Cupertino, S. G. Yeates, M. L. Turner and A. C. Spivey, *Org. Biomol. Chem.*, 2007, **5**, 1752-1763.
3. W.-H. Lee, S. K. Son, K. Kim, S. K. Lee, W. S. Shin, S.-J. Moon and I.-N. Kang, *Macromolecules*, 2012, **45**, 1303-1312.
4. C.-H. Tsai, A. Fortney, Y. Qiu, R. R. Gil, D. Yaron, T. Kowalewski and K. J. T. Noonan, *J. Am. Chem. Soc.*, 2016, **138**, 6798-6804.
5. Y.-Y. Lai, T.-C. Tung, W.-W. Liang and Y.-J. Cheng, *Macromolecules*, 2015, **48**, 2978-2988.
